# Does the Configuration at the Metal Matter in Noyori–Ikariya Type Asymmetric Transfer Hydrogenation Catalysts?

**DOI:** 10.1021/acscatal.1c03636

**Published:** 2021-10-26

**Authors:** Andrew M. R. Hall, Daniel B. G. Berry, Jaime N. Crossley, Anna Codina, Ian Clegg, John P. Lowe, Antoine Buchard, Ulrich Hintermair

**Affiliations:** † Centre for Sustainable & Circular Technologies, University of Bath, Claverton Down, Bath BA2 7AY, United Kingdom; ‡ Dynamic Reaction Monitoring Facility, University of Bath, Claverton Down, Bath BA2 7AY, United Kingdom; § Department of Chemistry, University of Bath, Claverton Down, Bath BA2 7AY, United Kingdom; ∥ Bruker UK Ltd., Banner Lane, Coventry CV4 9GH, United Kingdom

**Keywords:** asymmetric transfer hydrogenation, reaction monitoring, NMR spectroscopy, catalytic intermediates, stereochemistry

## Abstract

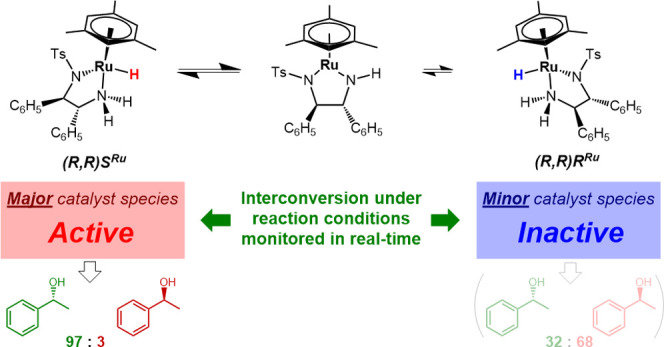

Noyori–Ikariya type [(arene)RuCl(TsDPEN)] (TsDPEN, sulfonated diphenyl ethylenediamine) complexes are widely used C=O and C=N reduction catalysts that produce chiral alcohols and amines via a key ruthenium–hydride intermediate that determines the stereochemistry of the product. Whereas many details about the interactions of the pro-chiral substrate with the hydride complex and the nature of the hydrogen transfer from the latter to the former have been investigated over the past 25 years, the role of the stereochemical configuration at the stereogenic ruthenium center in the catalysis has not been elucidated so far. Using *operando* FlowNMR spectroscopy and nuclear Overhauser effect spectroscopy, we show the existence of two diastereomeric hydride complexes under reaction conditions, assign their absolute configurations in solution, and monitor their interconversion during transfer hydrogenation catalysis. Configurational analysis and multifunctional density functional theory (DFT) calculations show the λ-(*R*,*R*)*S*^Ru^ configured [(mesitylene)RuH(TsDPEN)] complex to be both thermodynamically and kinetically favored over its λ-(*R*,*R*)*R*^Ru^ isomer with the opposite configuration at the metal. Computational analysis of both diastereomeric catalytic manifolds show the major λ-(*R*,*R*)*S*^Ru^ configured [(mesitylene)RuH(TsDPEN)] complex to dominate asymmetric ketone reduction catalysis with the minor λ-(*R*,*R*)*R*^Ru^ [(mesitylene)RuH(TsDPEN)] stereoisomer being both less active and less enantioselective. These findings also hold true for a tethered catalyst derivative with a propyl linker between the arene and TsDPEN ligands and thus show enantioselective transfer hydrogenation catalysis with Noyori–Ikariya complexes to proceed via a lock-and-key mechanism.

## Introduction

Asymmetric transfer hydrogenation catalysts are widely used in industry and synthetic laboratories as a safe, selective, and high yielding means of producing valuable chiral alcohols and amines from simple ketones and imines.^1−4^ The class of arene–ruthenium complexes with chiral sulfonated diphenyl ethylenediamine (TsDPEN) ligands developed in the mid 1990s by Noyori, Ikariya and co-workers have proved exceptionally effective in the asymmetric transfer hydrogenation reaction, offering excellent chemo- and stereoselectivity at high reaction rates, and have remained the gold standard in the field to date.^5,6^ The mechanism of these relatively simple yet highly effective catalysts has been studied in great detail, and their selectivity to preferentially reduce polar C=O and C=N bonds in the presence of C=C units is understood to be the result of metal–ligand bifunctional hydrogen transfer that preserves the polarization of H^–^ and H^+^ during turnover akin to classical Meerwein–Ponndorf–Verley (MPV) reductions.^2,7,8^ The high enantioselectivity of Noyori–Ikariya catalysts (typically greater than 90% enantiomeric excess (*ee*) in the alcohol product)^9,10^ is induced by the chiral diamine ligand with the stereochemistry of the alcohol produced predictably correlating with the stereochemistry of the ligand.

**Scheme 1:**
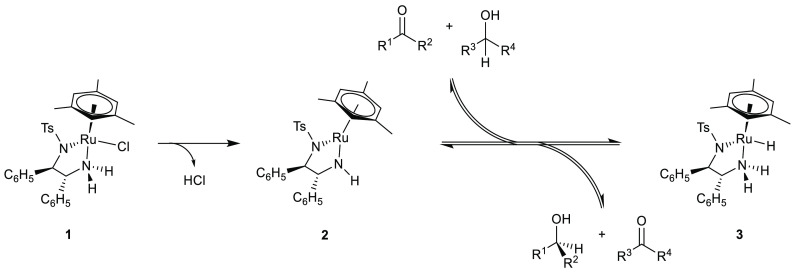
Simplified Schematic of the Noyori–Ikariya Asymmetric Transfer Hydrogenation Reaction Showing Catalyst Precursor **1** and Major in-Cycle Intermediates **2** and **3** Typically R^3^=R^4^=CH_3_.

The *in situ* generation of the active hydride complex from fairly air-stable chloride precursors such as [(mesitylene)Ru(*R*,*R*-TsDPEN)Cl] **1** has been shown to proceed in two steps. The addition of a moderately strong base (p*K*_A_ > 10) leads to rapid loss of the inner-sphere chloride and deprotonation of one of the amine protons, resulting in an unsaturated 16-electron bis-amido complex **2** (Scheme 1).^5^ Upon reaction with a primary or secondary alcohol such as propan-2-ol (typically used as the reaction solvent), the corresponding 18-electron hydride complex [(mesitylene)Ru(*R*,*R*-TsDPEN)H] **3** is formed by dehydrogenation of the alcohol to the corresponding carbonyl compound. Catalytic transfer hydrogenation of the substrate then occurs by repeated shuttling between intermediates **2** and **3** until the mixture reaches equilibrium. Due to the fully reversible nature of the interconversion of **2** and **3** via reaction with the 1-phenylethanol product, the high (but imperfect) initial enantioselectivity of the catalyst gradually diminishes the enantiomeric excess in the product over time. The replacement of propan-2-ol with formic acid results in the formation of carbon dioxide as a byproduct, which outgases from the reaction, suppressing the reverse reaction and preventing erosion of the initial high enantioselectivity.^10,11^ Excess base can exert a competitive inhibition effect on intermediate **2**, and irreversible deactivation processes occurring from intermediate **3** lead to a gradual reduction in reaction rate over time (not shown in the simplified reaction, Scheme 1).^12^

**Scheme 2:**
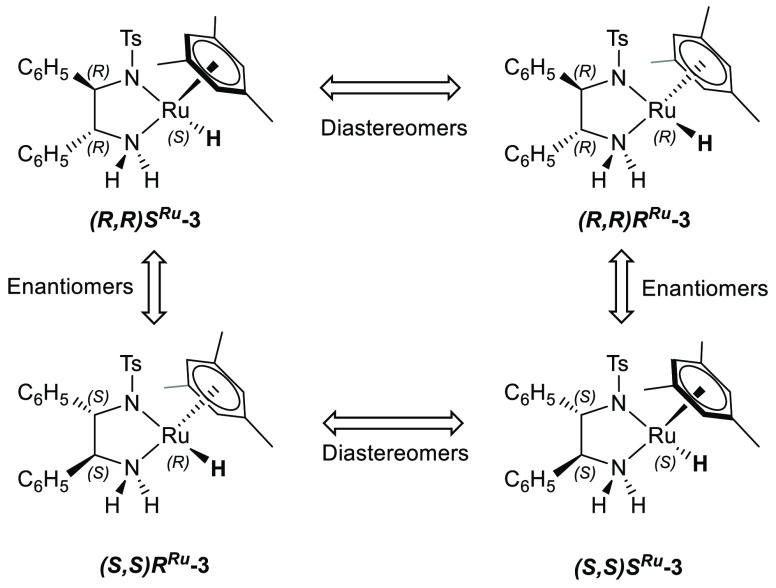
Structures and Relationships between Possible Stereoisomers of [(Mesitylene)RuH(TsDPEN)] Transfer Hydrogenation Catalysts

While the relationship between the stereochemistry of the chiral diamine ligand and the product is regularly correlated, the importance of the configuration at the metal center has received less consideration so far. For each of the enantiomers of the TsDPEN ligand, there exists a diastereotopic pair of Ru–H complexes **3** with different configurations at ruthenium (Scheme 2).^13−15^

**Scheme 3:**
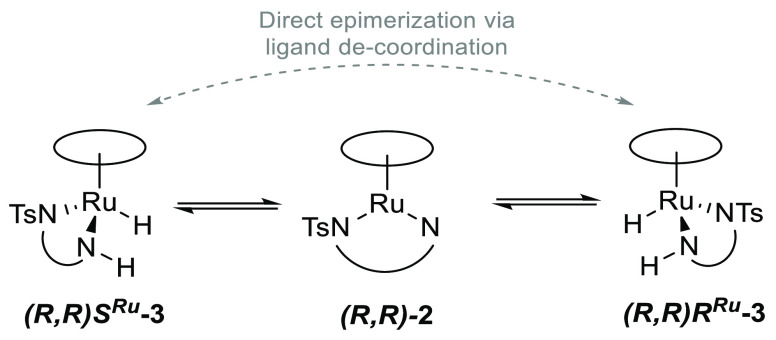
Simplified Depiction of the Interconversion of Diastereomeric Hydride Complexes (*R*,*R*)*S*^Ru^-**3** and (*R*,*R*)*R*^Ru^-**3** via the Achiral-at-Metal Amido Intermediate (*R*,*R*)-**2**

The experimental X-ray crystal structure for 16-electron amido complex **2** showed a distorted trigonal bipyramidal (dTBP) ligand field in which π contributions from both nitrogen lone pairs result in a coplanar 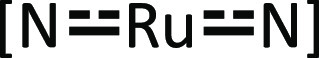 arrangement perpendicular to the arene in which the Ru atom is achiral.^5^ As the H^+^/H^–^ addition from isopropanol (or any other suitable reductant) across the Ru=NH moiety in **2** can occur from either diastereotopic face, the unsaturated amido intermediate **2** therefore acts as a natural crossing point in the catalytic cycle where the configuration at ruthenium may be inverted during the reaction in addition to direct epimerization pathways by temporary ligand decoordination (Scheme 3). While the absolute configuration at Ru in isolated samples of **1** and **3** has been established by X-ray crystallography,^5^ the possible interconversion between different diastereomers of **3** under the reaction conditions and the implication of this process for the rate and selectivity of the catalysis have not been clarified so far.

Evidence for the possible formation of a diastereomeric Ru–H pair in solution has been featured in several reports.^5,7,16−18^ Noyori and co-workers first observed a minor second peak ∼1% of the size of the major hydride in the ^1^H NMR spectrum of complex **3** in toluene at room temperature.^5^ On the basis of the configuration derived from XRD analysis of a sample crystallized from that mixture, they assigned the major diastereomer with a chemical shift of −5.47 ppm in toluene-*d*_8_ as (*S*,*S*)*R*^Ru^-**3**.^5^ In subsequent computational studies by Noyori and co-workers and later Dub and Gordon, only the (*S*,*S*)*R*^Ru^-**3** configured Ru–H complex has been considered relevant to catalysis,^19,20^ and the assumption that (*S*,*S*)*R*^Ru^-**3** forms from (*S*,*S*)-**2** and alcohol with high diastereoselectivity has become accepted in the field ever since.

Similar hydride diastereomers have been observed for tethered derivatives of Noyori’s catalyst,^21−25^ and for related rhodium and iridium complexes.^3,26−28^ However, the reported ratios of the two stereoisomers varied considerably with the concentration of minor hydride ranging from <1% up to 50% of the major hydride depending on reaction conditions and catalyst structure. Importantly, no configurational assignment of any of these hydride complexes in solution has been carried out so far, leaving an important gap in our understanding of the functioning of these widely used catalysts.

## Results and Discussion

### Conformational Effects

In addition to stereogenic centers, the conformation of the ligand bound to the metal center and the orientation of the N–H relative to the Ru–H have important implications for catalysis with these complexes. In the outer-sphere mechanism proposed by Noyori and others,^2,19,20^ both hydrogen atoms are transferred simultaneously via a 6-membered pericyclic transition state with the ketone substrate that requires a *syn*-coplanar H–Ru–N–H arrangement. Conformers with an *anti* (gauche) H–Ru–N–H arrangement are therefore expected to have a higher reaction barrier than those where the two hydrogen atoms are coplanar. Recent computational studies have found a stepwise H^–^/H^+^ transfer with proton shuttling by the solvent to be a plausible alternative.^29^ However, the same configurational requirements as in the concerted hydrogen transfer pathway prevail in this scenario. Multiple interactions between the prochiral substrate and the chiral pocket around the active NH–RuH center contribute to asymmetric induction, including CH−π attraction with the bound arene^30^ and LP−π repulsion by the sulfonyl group,^29^ confirming the key role of this hydride intermediate for effective catalysis.

**Scheme 4:**
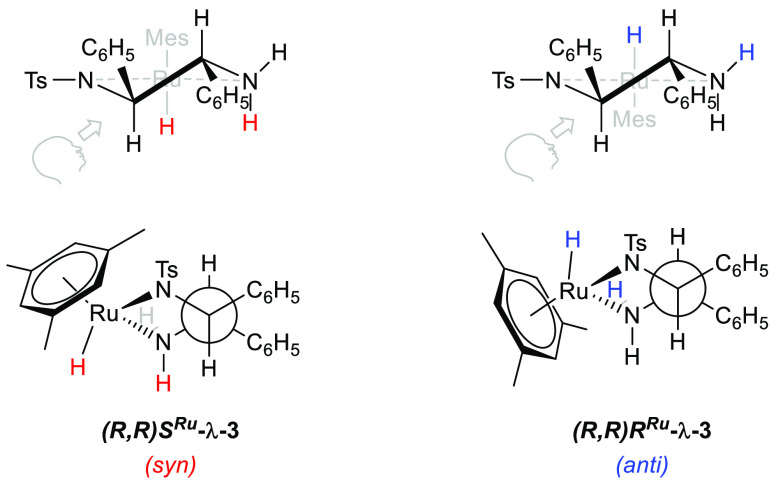
Expected Geometries for the Two Possible Diastereomers of [(Mesitylene)RuH(TsDPEN)] Resulting from the Inversion of Chirality at Ruthenium

For each of the two diastereomeric hydride complexes (*R*,*R*)*S*^Ru^-**3** and (*R*,*R*)*R*^Ru^-**3** there exist six different conformational isomers with different relative conformations around the C–C and C–N bonds in the ligand (Scheme S1). Conformation theory along with density functional theory (DFT) calculations suggest that the ligand backbone should adopt a gauche conformation (λ in the case of the *R*,*R* ligand) to optimize orbital overlap while minimizing steric clash between the two phenyl substituents and the π-bound arene (Scheme 4),^19,29,31^ just as observed in the reported crystal structure of (*S*,*S*)*R*^Ru^-**3**.^5,7^ This argument has been used to explain observed product enantioselectivities,^20^ but the confirmation of the absolute configuration of the major hydride complex **3** in solution, the possibility of interconversion during turnover by repeated passing through achiral-at-metal amido complex **2**, and the potential role of minor diastereomers of **3** in catalysis have been absent from mechanistic discussions so far.

### Hydride Speciation during Catalysis with [(Mesitylene)RuCl(TsDPEN)]

We have previously reported how online FlowNMR spectroscopy may be used to study the speciation and kinetics of Noyori’s catalyst during ketone transfer hydrogenation from isopropanol^12^ and from formic acid/triethylamine mixtures.^11^

**Scheme 5:**
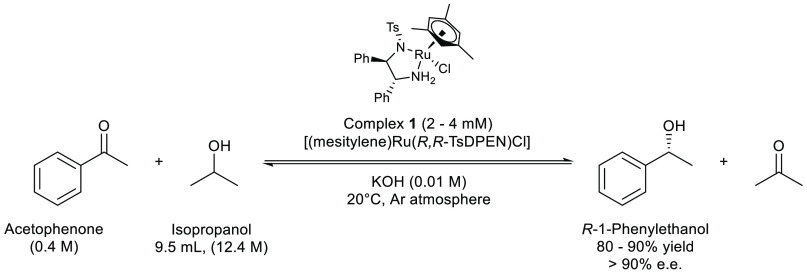
Standard Conditions Used for the Catalytic Asymmetric Transfer Hydrogenation of Acetophenone to (*R*)-1-Phenylethanol with [(Mesitylene)RuCl((*R*,*R*)-TsDPEN)] **1** Unless otherwise specified, catalysts with the (*R*,*R*)-TsDPEN ligand configuration were used in all experiments.

**Figure 1:**
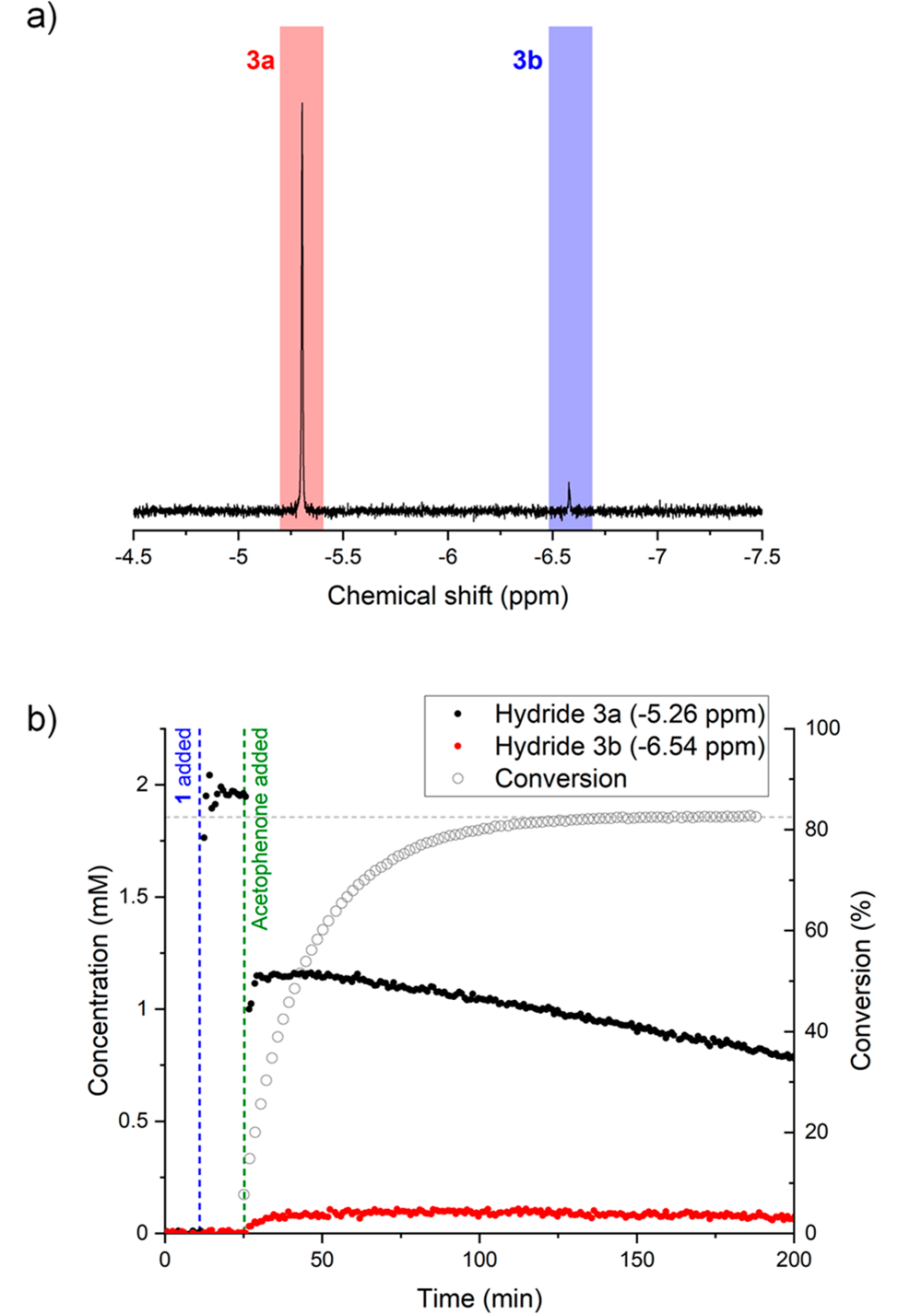
(a) ^1^H NMR spectrum of hydrides **3a** (−5.26 ppm) and **3b** (−6.54 ppm) and (b) product conversion and concentration profiles of hydrides **3a** and **3b** during the course of catalytic transfer hydrogenation of acetophenone to *R*-1-phenylethanol in flow at 4 mL/min (400 mM acetophenone, 10 mM KOH, 2 mM complex **1**, 9.5 mL of dry isopropanol, 20 °C). Selective excitation using a gradient spin echo pulse sequence with a shaped 180° pulse centered at −5.5 ppm (8 scans, 2 s acquisition time, 1 s delay time, 1600 μs Gaussian shaped pulse).

Using a gradient spin echo selective excitation pulse sequence, hydride complex **3** could be observed as a singlet at −5.26 ppm under turnover conditions in nondeuterated isopropanol. In addition to this resonance, a second, smaller singlet at −6.54 ppm was also detected during the course of the reaction (Figure 1a). Qualitatively, these chemical shifts are similar to those Noyori reported for the two diastereoisomers of **3** under similar conditions,^5^ although others have reported much smaller chemical shift differences (<0.1 ppm) in solvent mixtures containing formic acid.^18^ In order to probe the identity of these two species observed at −5.26 and −6.54 ppm during our *operando* FlowNMR studies, we measured their ^1^H spin–lattice relaxation times and molecular diffusion coefficients in solution. The similar *T*_1_ values (663 ± 8.2 and 469 ± 26.1 ms, respectively) and DOSY signatures (2.43 × 10^–10^ ± 1.04 × 10^–12^ and 2.40 × 10^–10^ ± 5.19 × 10^–12^ m^2^/s in isopropanol-*h*_8_)^32,33^ of these two species showed both to be monomeric Ru–H complexes with the same ligand set, ruling out association phenomena via hydrogen bonding or dimerization as a cause for the two different Ru–H environments (for details, see the Supporting Information). Furthermore, a control experiment replacing TsDPEN with the achiral *N*-tosyl-ethylene-1,2-diamine (TsEN) ligand produced the [(arene)Ru(TsEN)Cl] catalyst where the configuration at ruthenium leads to enantiomers rather than diastereomers. Subjecting this catalyst precursor to the same reaction conditions (Scheme 5) gave rise to racemic 1-phenylethanol production via catalytic hydrogen transfer from isopropanol but with a single hydride signal at −6.52 ppm throughout the reaction (Figure S1).

As can be seen from Figure 1b, hydride **3a** was observed to form immediately when the chloride complex **1** was dissolved in 0.01 M KOH solution in isopropanol. Although **3a** slowly deactivated,^12^ no other hydride species were observed until the addition of the acetophenone substrate at which point hydride **3b** started to form. Unlike **3a**, the formation of **3b** did not occur instantaneously but required around 30 min to reach equilibrium (*k*_obs_= 3.06 × 10^–2^ mM/min). This behavior supports the assignment of **3b** as a diastereomer of **3a** formed by an epimerization pathway that requires passing through the achiral intermediate **2** (Scheme 1), a process that is facilitated by the presence of substrate to turn over the catalyst.

**Figure 2:**
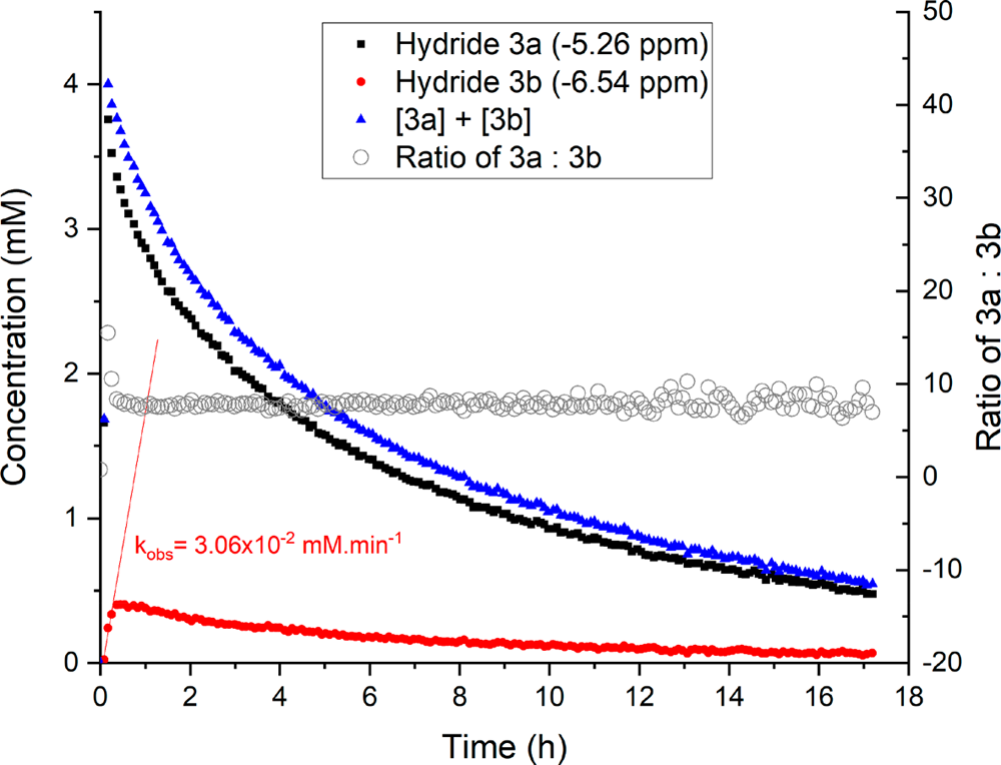
Concentration profiles of hydride peaks **3a** (−5.26 ppm) and **3b** (−6.54 ppm) in flow at 4 mL/min (40 mM acetophenone, 360 mM rac-1-phenylethanol, 360 mM acetone, 10 mM KOH, 4 mM (**1**), 9.5 mL of dry isopropanol, 20 °C). Selective excitation using a gradient spin echo pulse sequence with a shaped 180° pulse centered at −5.5 ppm (8 scans, 2 s acquisition time, 1 s delay time, 1600 μs Gaussian shaped pulse).

Both hydride species were observed to undergo slow deactivation over a period of several hours under reaction conditions (Figure 2). After formation of the initial equilibrium, the ratio between **3a** and **3b** remained unchanged at about 9:1 over more than 16 h, indicating either that the deactivation rates are identical for both diastereomers or that continued exchange between the two diastereomers maintains their relative ratio while one or both are deactivated.^34^ Repeating the experiment without substrate and in an inert solvent such as C_6_D_6_ resulted in a much slower rate of exchange between the two diastereomers (*k*_obs_= 9.13 × 10^–4^ mM/min; Figure S2), consistent with the notion that interconversion only occurs via hydrogen transfer passing through the achiral-at-ruthenium amido complex **2**.

### Absolute Configuration of [(Mesitylene)RuH(TsDPEN)] during Catalysis

**Scheme 6:**
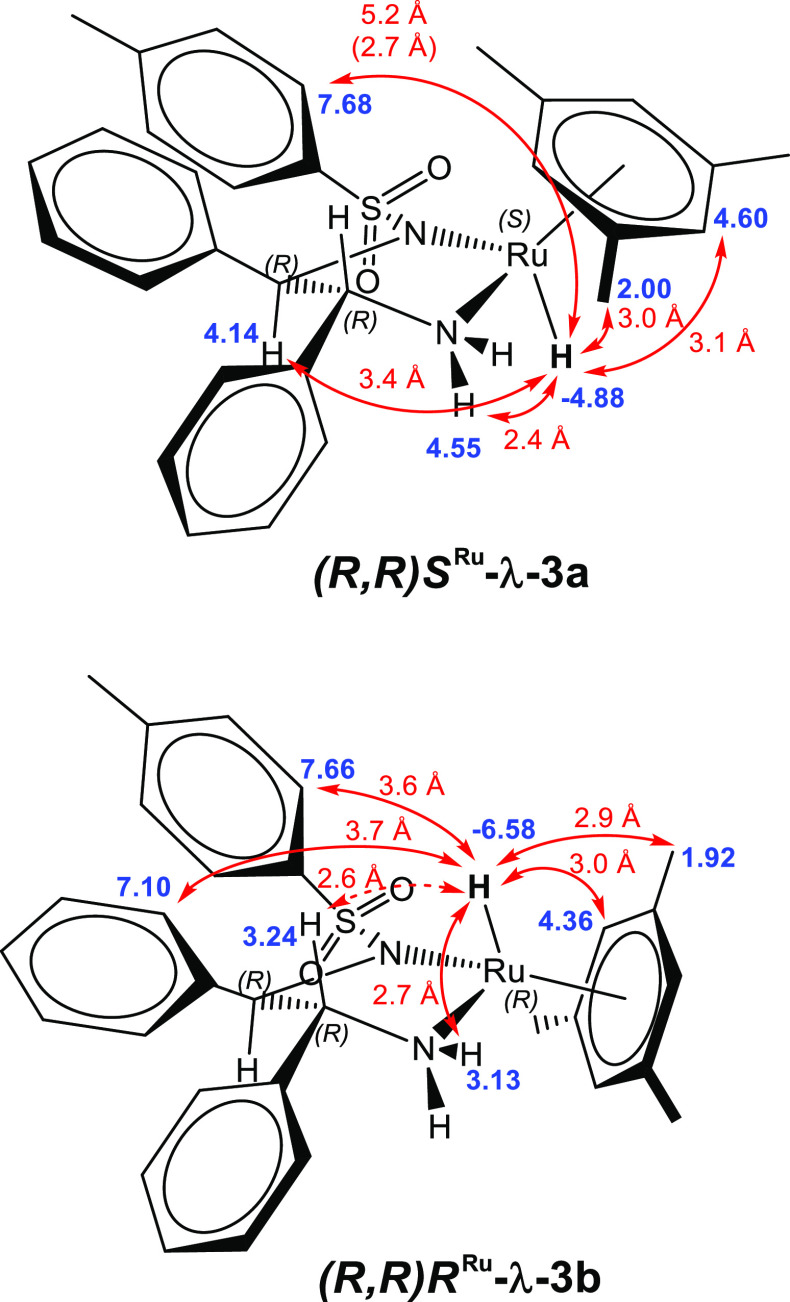
DFT Optimized Solution Structures and Experimental ^1^H NMR Chemical Shifts (C_6_D_6_) of Hydride Complexes (*R*,*R*)*S*^Ru^-**3a** and (*R*,*R*)*R*^Ru^-**3b** Showing Observed NOE Interactions and Contact Distances rωB97X-D(SMD:iPrOH)/SDD (Ru)/6-31+G(d) (Ph and Ts C,H)/6-311++G(d,p) (all other atoms).

In order to ascertain their relative configurations in solution, the configuration of the two hydride complexes was investigated using nuclear Overhauser effect (NOE) spectroscopy. Hydride **3a** (−4.88 ppm in C_6_D_6_) showed through-space correlations to the mesitylene CH_3_ and CH protons at 2.00 and 4.60 ppm, respectively, and tosyl *ortho*-CH protons at 7.68 ppm, along with the CH–NTs proton at 4.14 ppm and axial-NH proton at 4.55 ppm (Scheme 6 and Figure S7). While the Ru–H to tosyl-CH distance of 5.2 Å in the lowest energy conformation is relatively large for a NOE interaction, the rotation of the N–Ts bond would allow a closer contact distance of up to 2.7 Å. In all cases, a corresponding NOE interaction was observed at the hydride when the relevant ligand protons were excited.

The presence of a NOE interaction between the Ru–H and the CH–NTs proton indicates that the two nuclei are on the same face of the complex in **3a**. This is reinforced by the absence of any NOE interaction between the CH–NH_2_ proton at 4.02 ppm and the Ru–H, which despite being more closely connected through bonds, are on opposing faces of **3a** and therefore do not show any NOE contacts. Conversely, the mesitylene exhibited strong, mutual NOE interactions with the CH–NH_2_ and equatorial-NH protons but not with the CH-NTs proton. Collectively, this data is in excellent agreement with the assignment of **3a** resonating at −4.88 ppm to be the (*R*,*R*)*S*^Ru^-**3** diastereomer in the λ (*syn*) configuration, the same as that observed in crystal structures of isolated complex (*S*,*S*)*R*^Ru^-**3**.^5^

Hydride complex **3b** (−6.59 ppm in C_6_D_6_) showed NOE correlations to a different set of mesitylene peaks at 1.91 and 4.35 ppm, consistent with the different electronic environment of mesitylene in the (*R*,*R*)*R*^Ru^ complex. A very small NOE interaction was observed with peaks at 3.24 and 3.13 ppm, which were attributed to the CH–NH_2_ and equatorial-NH protons, respectively. A correlation was also observed to one of the phenyl CH protons, which is in agreement with the more closed structure of the (*R*,*R*)*R*^Ru^ diastereomer in the λ (*anti*) configuration, which twists the Ru–H away from the NH_2_ protons and brings it closer to the ligand phenyls (Scheme 6 and Figure S8).

Together with their similar *T*_1_ values and diffusion coefficients, these assignments clearly establish the two hydride resonances observed during hydrogen transfer catalysis with [(mesitylene)RuCl(TsDPEN)] as diastereoisomers of the key [(mesitylene)RuH(TsDPEN)] complex that differ in configuration at the metal, ruling out alternative explanations such as H-bonding, partial deligation, or conformational changes within the same stereoisomer.^35^ To confirm our NMR assignments and ascertain the absolute configuration of each structure, DFT calculations [rωB97X-D, rPBE1PBE, rB3PW91, or rM06L (SMD:iPrOH)/SDD (Ru)/6-31+G(d) (Ph and Ts C,H)/6-311++G(d,p) (all other atoms)] were used to predict the NMR chemical shifts of both diastereomers using four different functionals, calibrated against a series of reference Ru^II^-hydride complexes from the literature (Table S3). While the absolute chemical shifts computed varied, depending on the functional and solvation model used (Table S4), the (*R*,*R*)*R*^Ru^ diastereomer was consistently predicted to be more shielded than the (*R*,*R*)*S*^Ru^ diastereomer, fully in agreement with our NOE results. Thus, the identity and configuration of the two hydride species formed during transfer hydrogenation catalysis with Noyori’s catalyst have been firmly established for the first time.

### Mechanistic Implications for Catalysis

In principle, either or both of the two interconvertible hydride diastereomers may be responsible for the product formation. In the case where two isomeric reaction intermediates produce two different, noninterconverting products and their rate of exchange is fast compared to irreversible product formation, the reaction may be described using Curtin-Hammett control where the product distribution is determined by the difference in the rate-limiting transition state energies, ΔΔ*G*^‡^.^38−40^ One prominent example is Landis and Halpern’s “major–minor” double-manifold mechanism (minor catalyst intermediate forms the major product) that explains the workings of asymmetric olefin hydrogenation with chiral Rh/phosphine complexes.^36,37^ So far, no signs of major–minor behavior (such as inverse temperature effects) have been reported for Noyori’s catalyst, and structural analysis of the active site suggests that the more common “lock-and-key” mechanism (major catalyst intermediate forms the major product) prevails in these systems.

The outer-sphere mechanism originally proposed by Noyori and co-workers requires simultaneous transfer of both hydrogen atoms via a concerted 6-membered transition state.^19^ When it is assumed that both diastereomers of **3** adopt the λ-configuration (gauche; as predicted by DFT calculations and seen in the experimental crystal structure), the (*R*,*R*)*S*^Ru^-**3a** diastereomer would be expected to have a lower transition state energy barrier than (*R*,*R*)*R*^Ru^-**3b**, since both hydrogen atoms are correctly aligned for the cyclic transition state in the former (Scheme 6).^41^ Surprisingly, however, this has never been investigated in any of the numerous computational studies of this catalyst, and the relative activities and selectivities of the two diastereomeric Ru–H complexes have remained unknown. We therefore decided to investigate the role of each hydride diastereomer in catalysis using DFT calculations of the transition states.

**Scheme 7:**
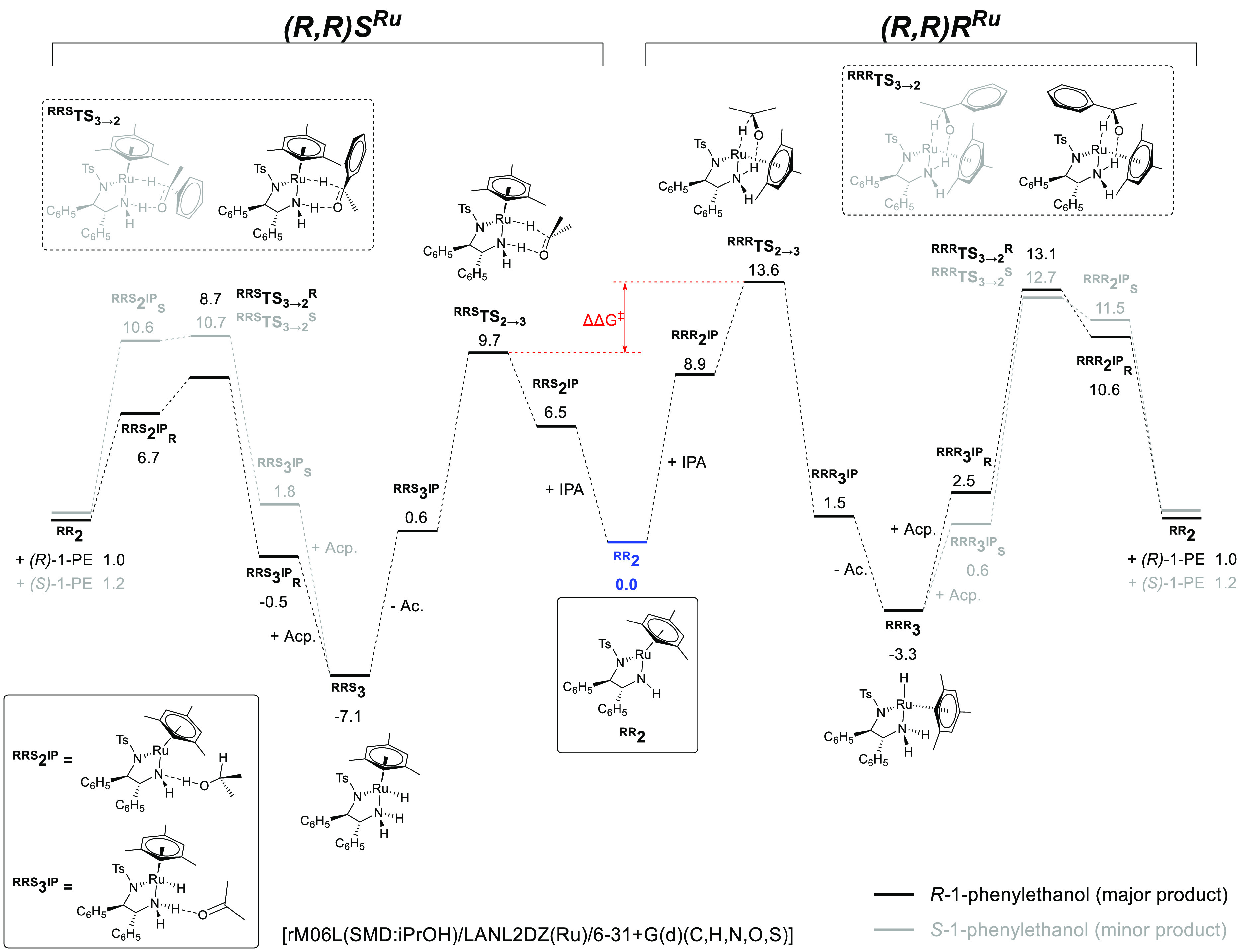
Proposed Mechanism and Calculated Ground and Transition State Energies (kcal/mol) for the Formation of *R*-1-Phenylethanol and *S*-1-Phenylethanol via Either the (*R*,*R*)*S*^Ru^ or (*R*,*R*)*R*^Ru^ Configured Catalyst See the Supporting Information for the details of the calculations. IPA = 2-propanol; Ac. = acetone; Acp. = acetophenone; 1-PE = 1-phenylethanol.

The reaction pathway for the (*R*,*R*)*S*^Ru^-λ catalyst configuration has been previously investigated by Dub and others,^19,20,29,42,43^ who identified low energy ion pairs formed between the hydride intermediate and substrate/product that act as pro-chiral resting states (labeled here as **2**^IP^ and **3**^IP^ for the alcohol and ketone ion pairs, respectively).^29^ In our own calculations using a slightly different basis set and functional than those used by Dub, the ion pairs were found to be intermediate in energy between the dissociated catalyst and transition state and are therefore unimportant when considering the reaction pathway. As shown in Scheme 7, the transition state leading to the formation of (*R*,*R*)*R*^Ru^-**3b** from the reaction of (*R*,*R*)-**2** with isopropanol was calculated to be disfavored by 3.9 kcal/mol (16.3 kJ/mol) relative to that for (*R*,*R*)*S*^Ru^-**3a**. Additionally, (*R*,*R*)*R*^Ru^-**3b** itself was predicted to be 3.8 kcal/mol (15.8 kJ/mol) higher in energy than (*R*,*R*)*S*^Ru^-**3a**, meaning that (*R*,*R*)*R*^Ru^-**3b** is both kinetically and thermodynamically disfavored, supporting a lock-and-key type mechanism. Using these values, the energy span model^44^ predicts that the rate of the reaction of (*R*,*R*)-**2** with isopropanol to produce (*R*,*R*)*S*^Ru^-**3a** should be around 790 times faster than for (*R*,*R*)*R*^Ru^-**3b**. This corresponds well with experimental results (Figure 1b) where (*R*,*R*)*S*^Ru^-**3a** was the only ruthenium species observed after activation of the catalyst in the absence of substrate. Under steady-state conditions with substrate, a reduced ratio of approximately 9:1 (*R*,*R*)*S*^Ru^-**3a** to (*R*,*R*)*R*^Ru^-**3b** was observed. The prediction of the equilibrium ratio of the two hydrides throughout many turnover events is complex and would necessitate including both forward and reverse rates of all reversible steps. Importantly, the calculations are in agreement with our experimental observations that saw the major (*R*,*R*)*S*^Ru^-**3a** hydride dominate throughout the reaction.

The transition states for the reaction of the hydride complexes with acetophenone were 4.4 and 2.0 kcal/mol (or 18.4 and 8.4 kJ/mol for *R*- and *S*-1-phenylethanol, respectively) lower for the (*R*,*R*)*S*^Ru^ pathway compared to (*R*,*R*)*R*^Ru^, indicating that the major (*R*,*R*)*S*^Ru^-**3a** catalyst intermediate is primarily responsible for the product formation.

These results strongly support a lock-and-key mechanism where the major catalyst intermediate is chiefly responsible for the product formation. Our *operando* FlowNMR data of the system aligns with this conclusion, as product formation began immediately upon addition of acetophenone to (*R*,*R*)*S*^Ru^-**3a** with no observable induction time. Major–minor control would require the establishment of a pre-equilibrium between the major and minor catalyst species prior to the rate-determining step,^36^ whereas in this case, (*R*,*R*)*R*^Ru^-**3b** was observed to only form after multiple turnovers (Figure 1b). The fact that interconversion of the diastereomeric hydrides appears to proceed exclusively through the unsaturated amido complex that is part of the catalytic product formation pathway further implies that the requirement for equilibration being significantly faster than product formation in a Curtin-Hammett type scenario is not met.

**Figure 3:**
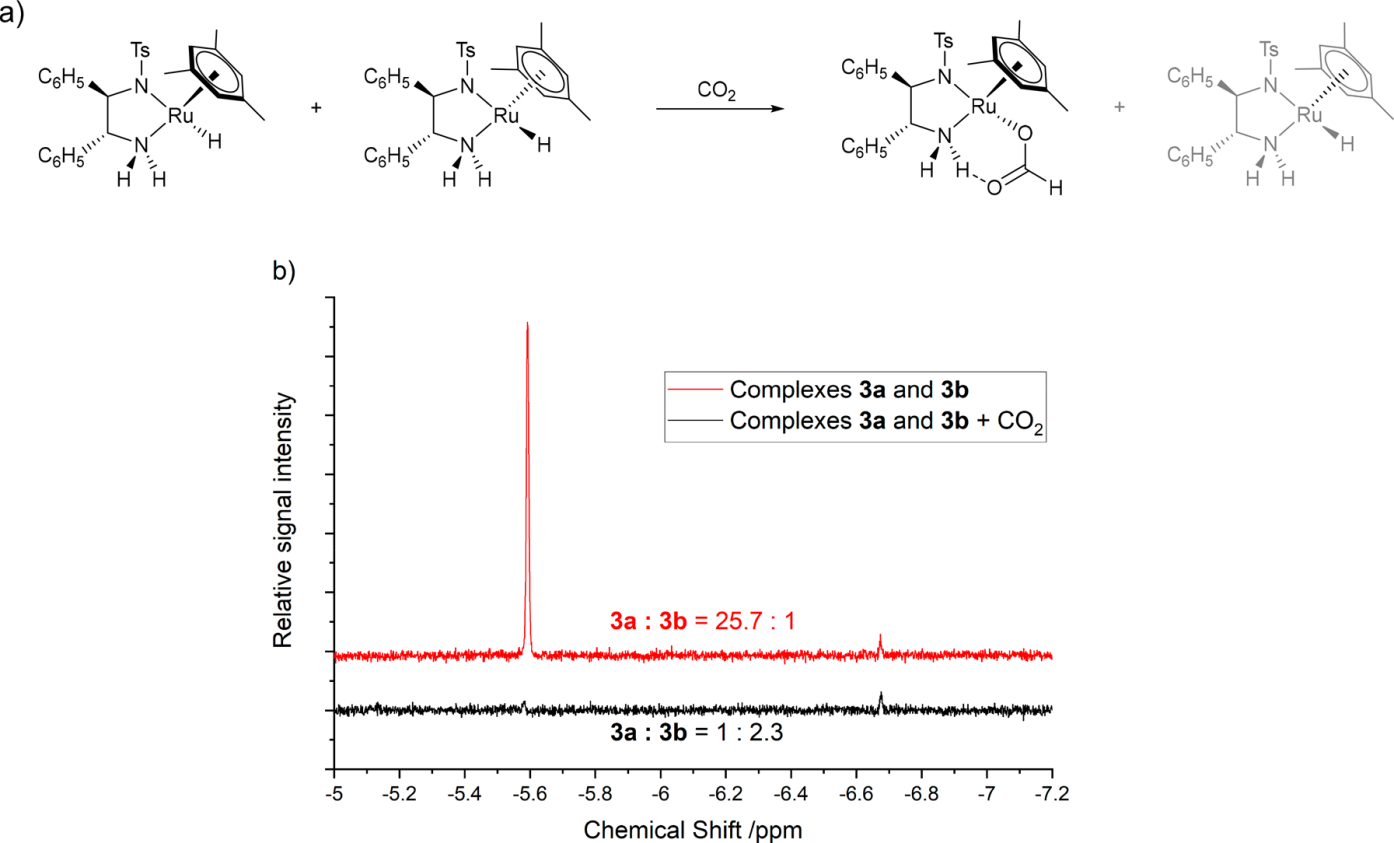
^1^H NMR spectra of (a) hydride complexes **3a** and **3b** and (b) hydride complexes **3a** and **3b** after pressurizing with 5 bar CO_2_ (THF-h_8_, 20 °C). Selective excitation using a gradient spin echo pulse sequence with a shaped 180° pulse centered at −9 ppm (12 scans, 1.33 s acquisition time, 1 s delay time, 1000 μs Gaussian shaped pulse).

Further experimental evidence for the predominant reactivity of the major hydride complex came from a competitive binding experiment with carbon dioxide. CO_2_ is known to react rapidly with hydride complex **3** to form a hydrogen-bond stabilized formate complex, which is the most abundant catalyst intermediate when transfer hydrogenation is carried out in triethylamine-formic acid solvent mixtures.^18^ The pressurization of a mixture of (*R*,*R*)*S*^Ru^-**3a** and (*R*,*R*)*R*^Ru^-**3b** in THF with 5 bar CO_2_ led to almost complete conversion of (*R*,*R*)*S*^Ru^-**3a** to its corresponding formate complex within 30 min at room temperature. (*R*,*R*)*R*^Ru^-**3b** on the other hand barely reacted at all, remaining virtually unchanged in solution (Figure 3). While not directly comparable to the reaction with an aryl-ketone in basic isopropanol, this observation nevertheless shows the higher reactivity of the major (*R*,*R*)*S*^Ru^ diastereomer of **3** toward C=O functionalities.

### Enantioselectivity Aspects

**Scheme 8:**
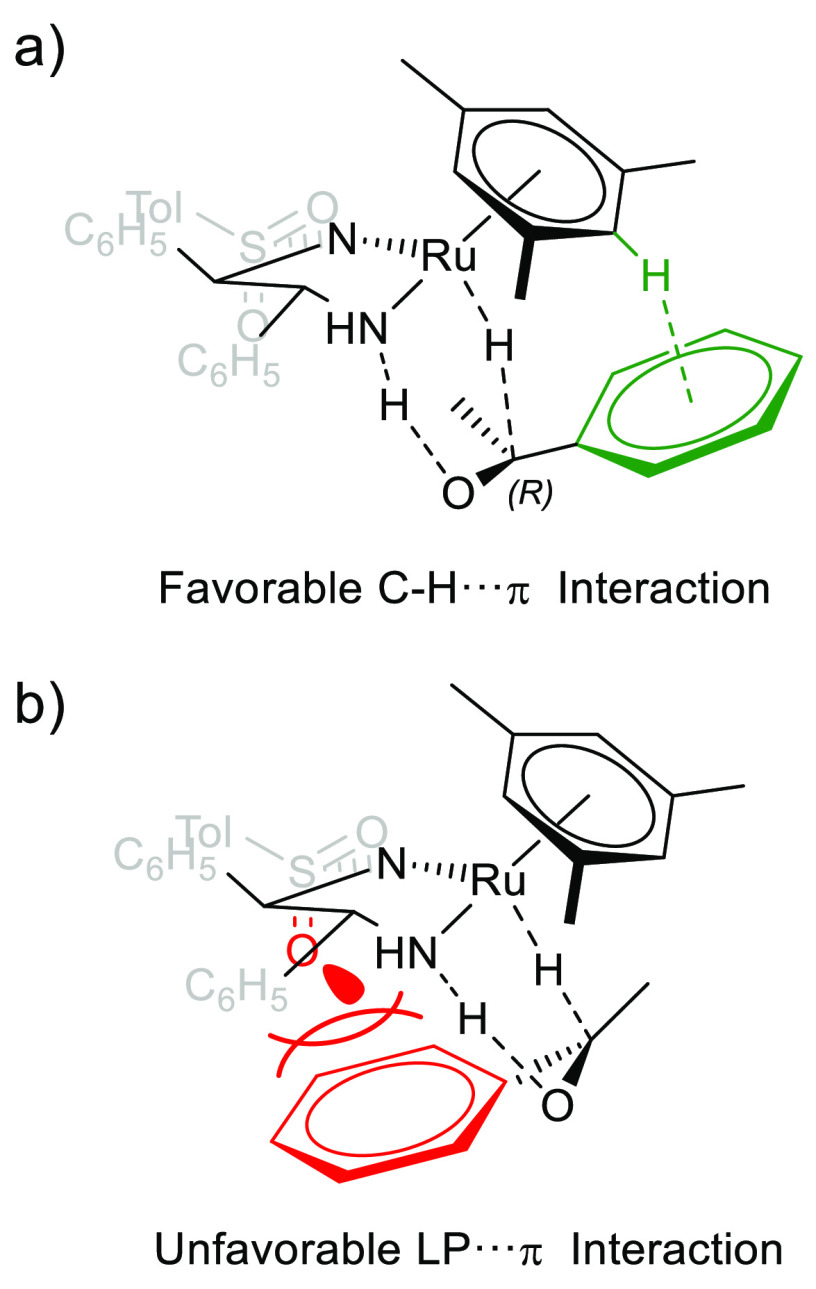
Schematic of (a) Favorable C–H···π Attractions between the Mesitylene and Acetophenone Arene Rings and (b) Unfavorable Oxygen Lone Pair-π Repulsion between the SO_2_ Group and Acetophenone in the Transition State of (*R*,*R*)*S*^Ru^-**3a** Reducing Acetophenone

Numerous experimental and computational studies of (*R*,*R*)-**3** and its derivatives have indicated that the stereochemistry of the product is driven by a C–H···π interaction between mesitylene and the acetophenone phenyl ring, which helps to stabilize the transition state for the formation of *R*-1-phenylethanol (Scheme 8a).^17,20,29,30,45−48^ Reverse binding to give the *S*-1-phenylethanol product was found to be disfavored by a repulsive interaction between the lone pair on the sulfone and the substrate arene ring, further reinforcing the product enantioselectivity (Scheme 8b).

Previous computational studies have focused on the (*R*,*R*)*S*^Ru^ catalyst pathway; however, in one instance, it has been speculated that inverting the configuration at ruthenium should impart the opposite product selectivity.^20^ In our own DFT calculations (Scheme 7), an energy difference of 2.0 kcal/mol (8.4 kJ/mol) was calculated for the transition states leading to *R*- and *S*-1-phenylethanol in the (*R*,*R*)*S*^Ru^ pathway. This is in excellent agreement with the value calculated by Dub and Gordon^20^ and predicts a 93% enantiomeric excess of *R*-1-phenylethanol (see the Supporting Information) just as observed experimentally. For the two product enantiomers in the (*R*,*R*)*R*^Ru^ pathway, our calculations gave a smaller energy difference of just 0.4 kcal/mol (1.7 kJ/mol), resulting in a predicted 36% ee toward *S*-1-phenylethanol. Combined with the overall higher transition state barrier for the (*R*,*R*)*R*^Ru^ pathway, it therefore seems likely that the predominant *R*-1-phenylethanol product is formed from the major (*R*,*R*)*S*^Ru^-**3a** catalyst diastereomer and that the minor *S*-1-phenylethanol product is formed predominantly from the less favorable ^***RRS***^**TS**_**3→2**_^**S**^ transition state with limited involvement from the less reactive (*R*,*R*)*R*^Ru^-**3b** catalyst diastereomer.

### Extension to Tethered Catalyst Derivatives

**Scheme 9:**
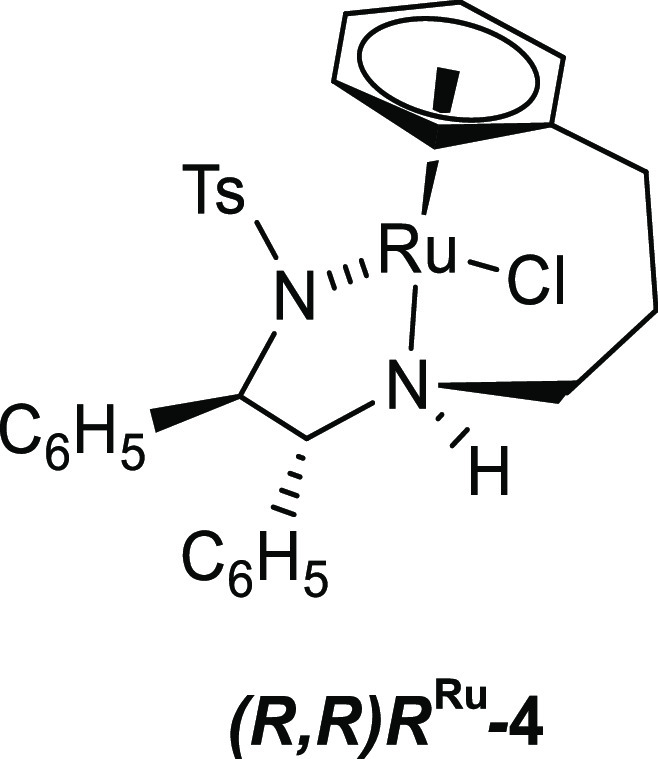
Structure of Wills’ C3-Tethered Complex **4**

As shown in Figure 2, both hydride complexes (*R*,*R*)*S*^Ru^-**3a** and (*R*,*R*)*R*^Ru^-**3b** partially deactivated over the course of the reaction, limiting the ability to analyze product formation rates at different distributions of both species over longer time periods. Tethered versions of the original Noyori–Ikariya catalysts with ether or alkyl linkages between the π-bound arene and the chiral diamine ligand have been developed to increase catalyst stability by limiting deactivation through arene decoordination.^21,22,25,49−52^ Due to the linker attachment on the nitrogen, the more strongly σ-donating secondary amine stabilizes the unsaturated amido complex in these systems so that it dominates catalyst speciation, contrary to the nontethered catalyst where hydride intermediate **3** comprises >90% of the catalyst speciation prior to entering turnover and about 55% during turnover in the steady state. One example of such a catalyst is Wills’ C3-tethered complex **4** (Scheme 9).^21^

**Scheme 10:**
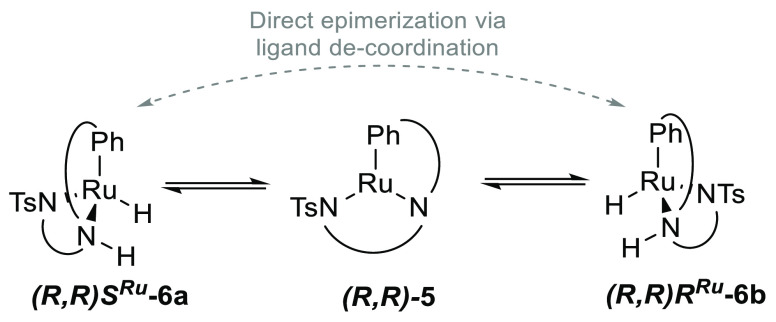
Interconversion of Hydride Complexes (*R*,*R*)*S*^Ru^-**6a** and (*R*,*R*)*R*^Ru^-**6b** via the Achiral-at-Metal Intermediate (*R*,*R*)-**5**

As in the nontethered version, two diastereomeric hydride complexes are possible, and as the ^*n*^propyl tether is long enough to allow the formation of the amido complex **5**, it should not prevent configurational interconversion at the ruthenium center during catalysis (Scheme 10). Indeed, two hydride resonances have been observed for this complex with the major hydride peak at −5.26 ppm and the minor hydride at −5.51 ppm in isopropanol. In benzene-*d*_6_, the population changed to a 1:5 ratio of the two hydrides at −4.50 and −5.10 ppm as *“two diastereoisomers, the relative configurations of which have not yet been fully assigned”*.^21^

NOE experiments with (*R*,*R*)-**6** under the same conditions as for (*R*,*R*)-**3** confirmed that the tethered catalyst exhibits the same configuration as the nontethered hydrides with the major hydride at −5.26 ppm corresponding to (*R*,*R*)*S*^Ru^-**6a** in the λ (*syn*) configuration (see the Supporting Information). DFT calculation [rωB97X-D, rPBE1PBE, rB3PW91, rM06L, rB3LYP, rM06, or rMPW1PW91 (SMD:iPrOH)/SDD (Ru)/6-31+G(d) (Ph and Ts C,H)/6-311++G(d,p) (all other atoms)] of the ^1^H NMR chemical shifts of (*R*,*R*)*S*^Ru^-**6a** and (*R*,*R*)*R*^Ru^-**6b** produced mixed results with most of the functionals tested predicting a reversal of the peak order compared to that observed experimentally (Table S5).

**Figure 4:**
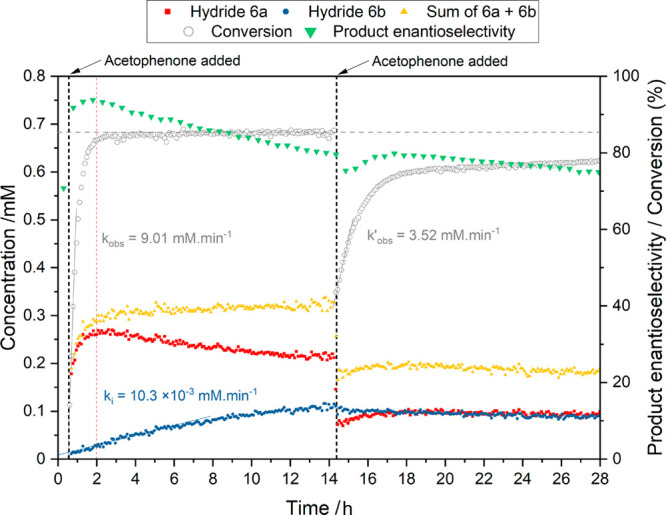
Concentration of hydride complexes (*R*,*R*)*S*^Ru^-**6a** (−5.26 ppm) and (*R*,*R*)*R*^Ru^-**6b** (−5.51 ppm), conversion, and product enantioselectivity data for the asymmetric transfer hydrogenation of acetophenone to (*S*)-1-phenylethanol (4 mM (*R*,*R*)-**4**, 20 mM KOH, 400 mM acetophenone (additional 400 mM acetophenone added after 14.5 h), 38 mL of isopropanol, 20 °C). Selective excitation using a gradient spin echo pulse sequence with a shaped 180° pulse centered at −5.5 ppm (96 scans, 2 s acquisition time, 1 s delay time, 1600 μs Gaussian shaped pulse). Note: The minor increase in conversion after 20 h is due to evaporation of acetone (formed as a byproduct of the reaction), leading to a shift in the equilibrium position.

Following hydrogen transfer, catalysis with (*R*,*R*)-**4** under the same conditions as for (*R*,*R*)-**1** (Figure 1, 4 mM cat., 20 mM KOH, 400 mM acetophenone, 10 mL of isopropanol, 20 °C) with *operando* FlowNMR spectroscopy showed a similar reaction profile (Figure 4). Due to the catalyst speciation being dominated by (*R*,*R*)-**5** as a result of the tether, lower reaction rates than for the nontethered catalyst were observed (*k*_obs_ = 9.01 mM/min, compared to 18.7 mM/min for **1**). Two distinct regimes were found for the amount of (*R*,*R*)-**6** present: immediately after adding substrate to the preactivated catalyst (>99% (*R*,*R*)*S*^Ru^-**6a**), the amount of hydride fell to zero and then gradually recovered again to ∼12% alongside buildup of the product. This behavior is consistent with the two-step kinetic model proposed by Wills and co-workers^22^ in which the reaction of (*R*,*R*)-**6** with ketone is faster than the generation of (*R*,*R*)-**6** from (*R*,*R*)-**5**, predicting that the amount of hydride will increase as the substrate is depleted in the presence of excess reductant (isopropanol solvent in this case). Unlike with the nontethered catalyst where hydride **3** suffers irreversible deactivation due to gradual arene loss (with a *k*_d_ = 6.35 × 10^–3^ mM/min),^12^ (*R*,*R*)*S*^Ru^-**6a** was more stable but continued to progressively interconvert to (*R*,*R*)*R*^Ru^-**6b** over at least 14 h with a rate of *k*_*i*_ = 10.3 × 10^–3^ mM/min.

When a second aliquot of acetophenone was added after the ratio of (*R*,*R*)*S*^Ru^-**6a** to (*R*,*R*)*R*^Ru^-**6b** had reached about 2:1, product formation resumed with a 2.5 times reduced rate of *k*_obs_^′^ = 3.52 mM/min. Product enantioselectivity in the mixture continued the slowly decreasing trend of the initial reaction due to continued catalyst cycling between alcohol and ketone. Crucially, however, when the second aliquot of acetophenone was added, only the major hydride (*R*,*R*)*S*^Ru^-**6a** was observed to change in concentration with (*R*,*R*)*R*^Ru^-**6b** remaining constant, demonstrating that the minor hydride complex (*R*,*R*)*R*^Ru^-**6b** was not involved in the catalytic product formation.

## Conclusions

We have demonstrated how selective excitation NMR techniques combined with online FlowNMR spectroscopy can be used to detect low concentration hydride species during an asymmetric transfer hydrogenation reaction using Noyori’s TsDPEN catalyst. To the best of our knowledge, this is the first example where both major and minor hydride species involved in this widely used chemistry have been observed and their kinetics quantified under reaction conditions. Using nuclear Overhauser effect spectroscopy and DFT calculations, we have shown the major species observed in the NMR spectra of the reaction mixture with [(mesitylene)RuCl((*R*,*R*)TsDPEN)] to be the *S*^Ru^-configured diastereomer with the *R*^Ru^ diastereomer representing a minor species. Interconversion of the two hydride complexes mainly occurs through the unsaturated 16-electron amido complex that is part of the catalytic cycle, explaining why isolated samples of [(mesitylene)RuH((*R*,*R*)TsDPEN)] show only one hydride resonance in the ^1^H NMR spectrum. The major hydride species (*R*,*R*)*S*^Ru^-**3a** has the most favorable geometry with a *syn*-coplanar NH–RuH configuration for efficient hydrogen transfer to aryl ketones and also demonstrated a markedly higher reactivity with CO_2_ than (*R*,*R*)*S*^Ru^-**3b** with the opposite configuration at ruthenium. DFT calculations support the spectroscopic assignments and showed the mechanism of asymmetric transfer hydrogenation catalysis to be dominated by the major hydride complex in a lock-and-key fashion.

The same conclusions hold true for a tethered version of the original Noyori–Ikariya catalyst, which is often preferred in industrial applications due to its increased robustness. Although the tether successfully prevents deactivation by way of ligand loss, progressive interconversion into an essentially inactive diastereomer still occurs in these systems, a process that emerges as the primary reason for reduced performance upon prolonged catalyst use and recycling.

Although the minor hydride complexes have been shown to be orders of magnitude less reactive with acetophenone than the major hydride with opposite configuration at ruthenium (notably with the same ligand), the fact that they are predicted to favor the opposite product enantiomer shows that knowledge of and control over chirality at the metal are important factors in asymmetric homogeneous catalysis with enantiopure ligands. Although stereochemical lability in chiral-at-metal complexes has been well studied in organometallic chemistry,^53−56^ this phenomenon has received less attention in homogeneous catalysis where certain reaction intermediates may be chiral-at-metal (for example, in metathesis^57^ or epoxidation,^58^ in addition to chiral-at-metal-only systems^59−62^). The lower enantioselectivities observed with some derivatives of the Noyori–Ikariya catalyst^49,63^ and/or the use of substrates other than simple aryl-alkyl ketones^23,45^ may in part be the result of less effective discrimination between the major and minor hydride complexes transforming the prochiral substrate with different degrees or even senses of stereoselectivity. As both rate and enantioselectivity in these bifunctional NH–RuH catalysts are strongly influenced by the relative orientation of key functionalities in the chiral pocket, we believe our findings may also extend to analogous arene complexes with TsDPEN-like ligands based on rhodium and iridium and hopefully offer useful insights for the development of new catalyst systems based on similar principles.

## Supporting Information Available

Full experimental details, additional figures, full NMR assignment of hydride complexes, and DFT calculated minimum energy geometries (PDF)

## Notes

For primary NMR data files and DFT calculation outputs, see 10.6084/m9.figshare.14473035.
