# Supplementary material for: Does the Configuration at the Metal Matter in Noyori–Ikariya Type Asymmetric Transfer Hydrogenation Catalysts?
Source: ACS Catal. 2021 Oct 26;11(21):13649–59. doi: 10.1021/acscatal.1c03636 (PMC8576814; doi:10.1021/acscatal.1c03636)
Supplement: Supplementary file 1 — cs1c03636_si_001.pdf [file cs1c03636_si_001.pdf]

Does the configuration at metal matter in Noyori-Ikariya type asymmetric transfer hydrogenation catalysts?

Andrew M. R. Hall[a,b], Daniel B. G. Berry[b,c], Jaime N. Crossley[c], Anna Codina[d], Ian Clegg[d], John P. Lowe[b,c], Antoine Buchard[a], Ulrich Hintermair*[a,b]

[a] *Centre for Sustainable & Circular Technologies, University of Bath, Claverton Down, Bath BA2 7AY, UK.*

[b] *Dynamic Reaction Monitoring Facility, University of Bath, Claverton Down, Bath BA2 7AY, UK.*

[c] *Department of Chemistry, University of Bath, Claverton Down, Bath BA2 7AY, UK.*

[d] *Bruker UK Ltd, Banner Lane, Coventry CV4 9GH, UK.*

* *u.hintermair@bath.ac.uk*

Contents

Additional figures 3

Experimental methods 6

- Catalyst synthesis 6
  - Ts(ethylenediamine) 6
  - $[RuCl_2(mesitylene)]_2$ 6
  - RuCl(Ts(ethylenediamine))(mesitylene). 6
  - Unsaturated intermediate, **2** 7
  - Hydride intermediate, **3** 7
- FlowNMR experiments 8
  - General procedure 8
  - Receiver Gain calibration 10
  - Observation of hydrides under reaction conditions (Figure 1) 11
  - Reaction with achiral Ts(ethylenediamine) ligand (Figure S1) 11
  - Deactivation under reaction conditions (Figure 2) 11
  - Tethered catalyst (Figure 4) 11
- Online monitoring with HPLC (Figure 4) 12
- Conventional NMR experiments 13
  - Deactivation in absence of substrate (Figure S2) 13
  - Reaction with CO_2 (Figure 3) 13
  - Diffusion measurements 14
  - T_1 measurements 16
  - Nuclear Overhauser Effect (NOE) spectra 17

NMR assignment and NOE experiments 18

- Hydrides **3a** and **3b** 18
- Hydrides **6a** and **6b** 34

Density functional theory calculations 44

- Prediction of ^1H NMR shifts 44
- Reaction pathway 47
- Energy span calculations 49
  - Catalyst stereochemistry 49
  - Product enantioselectivity 50

References 51

Additional figures

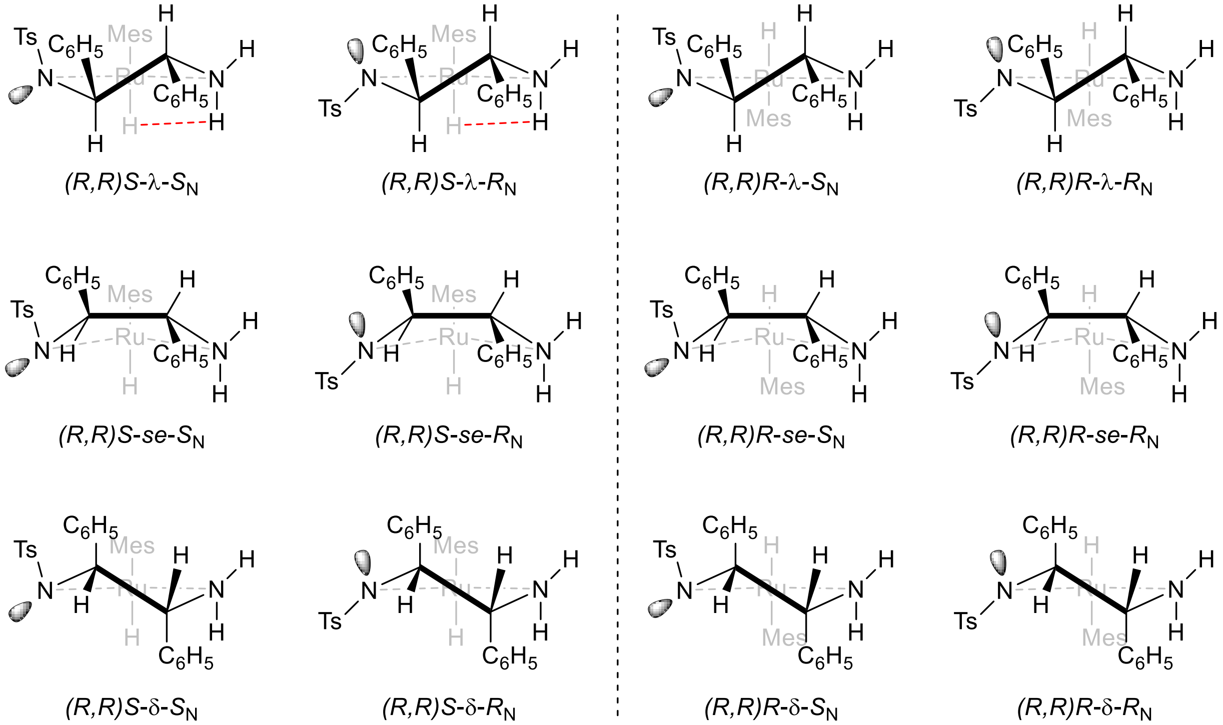

Scheme S1: Possible conformations for the (R,R)R and (R,R)S diastereomers of RuH(TsDPEN)(mesitylene).

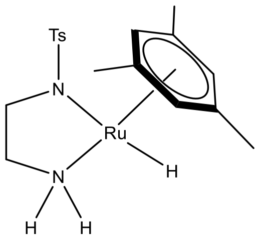

Scheme S2: Structure of [(mesitylene)RuH(Ts(ethylenediamine))].

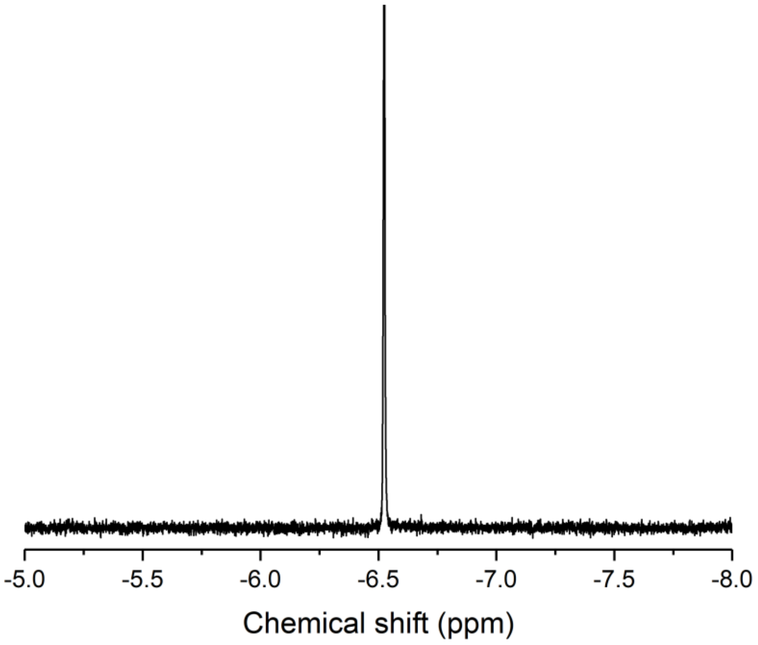

Figure S1: ^{1}H NMR spectrum of [(mesitylene)RuH(Ts(ethylenediamine))] during the course of catalytic transfer hydrogenation of acetophenone to (R)-1-phenylethanol in flow at 4 mL/min (400 mM acetophenone, 10 mM KOH, 2 mM RuCl(Ts(ethylenediamine))(mesitylene), 9.5 mL dry isopropanol, 20°C). Selective excitation using a gradient spin echo pulse sequence with a shaped 180° pulse centred at -5.5 ppm (8 scans, 2 s acquisition time, 1 s relaxation delay time, 1600 µs Gaussian shaped pulse; spectra processed with 0.1 Hz exponential line broadening).

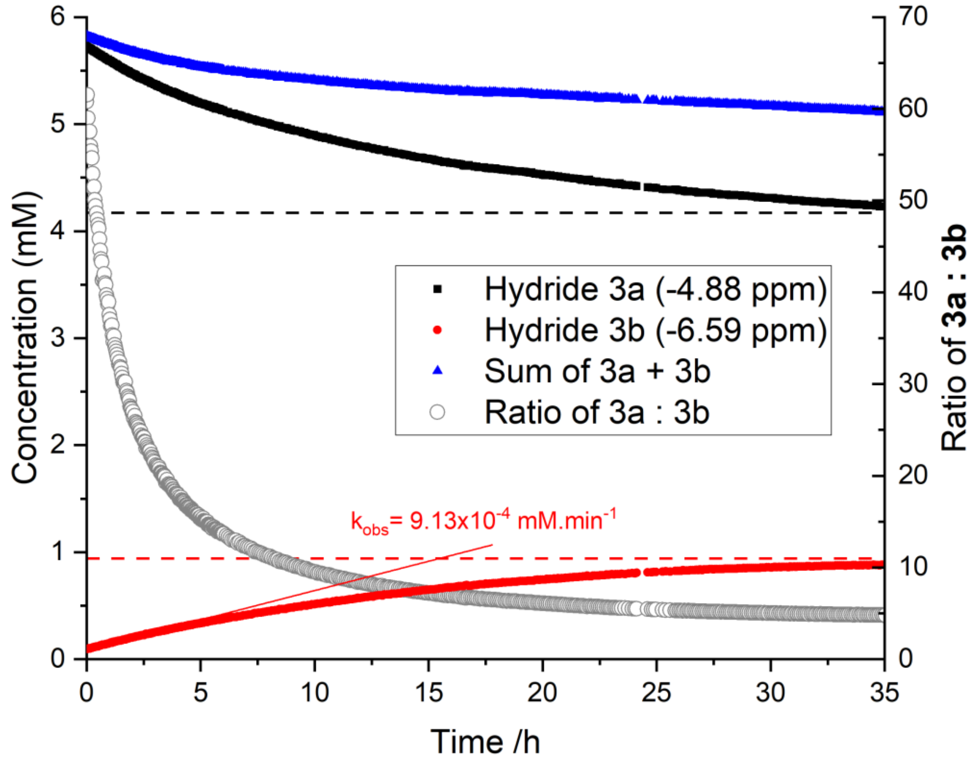

*Figure S2: Concentration profiles of hydride peaks **3a** (-4.88 ppm) and **3b** (-6.59 ppm), during the interconversion between diastereomers **3a** and **3b** in the absence of product or substrate. (6 mM (**1**), 12 mM NaO^{i}Pr, 0.5 mL C_6D_6, 20 µL isopropanol, 25 °C, Ar atmosphere, Young's tap NMR tube). Selective excitation using a gradient spin echo pulse sequence with a shaped 180° pulse centred at -5.5 ppm (8 scans, 2 s acquisition time, 1 s relaxation delay time, 1600 µs Gaussian shaped pulse; spectra processed with 0.1 Hz exponential line broadening).*

Experimental methods

Catalyst synthesis

Ruthenium(III) chloride and complex **1** were purchased from Alfa Aesar. Tethered catalyst **4** was supplied by Johnson Matthey. $[RuCl_2(mesitylene)]_2$ was synthesised according to literature procedure.[1] All other compounds were purchased from Sigma Aldrich and used without further purification unless specified. Unless otherwise specified, all reactions were carried out under argon atmosphere with dry and degassed solvents. THF, triethylamine and acetophenone were all freshly distilled under inert conditions before use.

NMR spectra were collected on a Bruker AVANCE III 500 MHz NMR spectrometer equipped with a 5 mm broadband observed (BBO) Prodigy CryoProbe.

Ts(ethylenediamine)

Synthesis was modified from a literature procedure.[2] Ethylene diamine (0.27 mL, 4.8 mmol) and triethylamine (2 mL, 14.3 mmol) were dissolved in dry THF (40 mL) and cooled to 0 °C in an ice bath. A solution of *para*-toluene sulfonyl chloride (0.9 g, 4.8 mmol) in THF (10 mL) was added dropwise to the reaction over 30 min. The mixture was warmed to room temperature before stirring overnight. The mixture was washed with saturated sodium hydrogen carbonate solution (80 mL) and dichloromethane (80 mL). The organic phase was washed with brine and evaporated to give a white powder. 0.85 g, 83%, m.p. 116 – 117 °C [lit. 118– 120 °C][3]. ^{1}H NMR (500 MHz, $CDCl_3$) δ: 7.74 (d, 2H, J_{HH}= 7.3 Hz, Ts arom.), 7.28 (d, 2H, J_{HH}= 7.5 Hz, Ts arom.), 3.67 (br, 3H, N<u>H</u>), 2.96 (t, 2H, J_{HH}= 5.2 Hz, NC<u>H</u>), 2.83 (t, 2H, J_{HH}= 5.0 Hz, NC<u>H</u>), 2.40 (s, 3H, TsC<u>H</u>$_3$).

$[RuCl_2(mesitylene)]_2$

Synthesis was performed according to a literature procedure.[1] Ruthenium trichloride hydrate (0.5 g, 1.9 mmol) and 1,3,5-trimethylcyclohexyl-1,4-diene (1.4 mL, 9.4 mmol) were dissolved in absolute ethanol (20 mL) and heated at reflux for 16 h. The mixture was allowed to cool, before filtering to isolate an orange-brown powder which was washed with ethanol (2 x 5 mL) and dried under high vacuum. 0.38 g, 68%. ^{1}H NMR (400 MHz, DMSO-d_6) δ: 5.46 (s, 1H), 2.15 (s, 3H).

RuCl(Ts(ethylenediamine))(mesitylene).

Synthesis was modified from a literature procedure.[2] Mono-tosyl ethylene diamine (0.2 g, 0.93 mmol), $[RuCl_2(mesitylene)]_2$ (0.28 g, 0.47 mmol) and triethylamine (0.5 mL, 3.58 mmol) were dissolved in isopropanol (20 mL) and heated to 80 °C for 2 h. The volume of the mixture was reduced to approx. 10 mL under vacuum and filtered to remove a fine black solid. The bright orange filtrate was

evaporated to give an orange crystalline solid, which was washed with water (approx. 2 mL) and dried under high vacuum. 0.39 g, 89%. ^{1}H NMR (500 MHz, $CDCl_3$) δ: 7.66 (d, 2H, J_{HH}= 8.0 Hz, Ts arom.), 7.06 (d, 2H, J_{HH}= 7.8 Hz, Ts arom.), 5.28 (s, 3H, mesitylene CH), 2.85 (br, 2H, CH_2), 2.63 (br, 2H, CH_2), 2.25 (s, 3H, Ts CH_3), 2.20 (s, 9H, mesitylene CH_3).

Unsaturated intermediate, 2

Unsaturated intermediate **2** was prepared according to a modified literature procedure.[4] Complex **1** (80 mg, 0.13 mmol) was dissolved in dry DCM (5 mL) to give a bright orange solution. A solution of potassium tert-butoxide (0.13 mL, 1M in THF, 0.13 mmol) was added, causing the solution to immediately turn deep purple in colour. The mixture was stirred for 5 mins at room temperature before filtering. The purple filtrate was evaporated under vacuum and the resulting purple solid dried overnight under vacuum to give an air-sensitive purple powder: Isolated yield: 0.029 g, 36%. λ_{max} = 565 nm, ϵ_{565} = 1377 mol^{-1}dm^{3}cm^{-1}. ^{1}H NMR (500 MHz, C_6D_6) δ: 8.11 (d, 2H, J_{HH}= 8.2 Hz, Ts (*meta*)), 7.06 (m, 4H, phenyl), 6.88 (d, 2H, J_{HH}= 7.8 Hz, Ts (*ortho*)), 6.76 (t, 2H, J_{HH}= 7.6 Hz, phenyl), 6.59 (d, 2H, J_{HH}= 7.7 Hz, phenyl), 4.83 (s, 3H, mesitylene CH), 4.11 (d (br), 1H, J_{HH}= 10.1 Hz, NH), 3.91 (d, 1H, J_{HH}= 10.6 Hz, C<u>H</u>-NTs), 3.63 (ddd, 1H, J_{HH}= 13.0, 10.2, 2.4 Hz, C<u>H</u>-NH), 2.09 (s, 9H, mesitylene CH_3), 1.94 (s, 3H, tosyl CH_3). ^{13}C NMR (125 MHz, C_6D_6) δ: 141.7, 140.6, 139.7, 139.5, 130.0, 129.1, 128.4, 127.8, 127.1, 126.9, 126.7, 99.6, 78.4, 75.1, 69.7, 20.8, 18.7.

Hydride intermediate, 3

Hydride intermediate **3** was prepared according to a modified literature procedure.[4] Unsaturated intermediate **2** was dissolved in dry isopropanol (5 mL) to give a yellow-brown solution. Solvent was evaporated under vacuum to give a brown powder. (See below for NMR assignment).

FlowNMR experiments

Reactions were carried out at room temperature (approximately 20°C) in a standard glass round-bottomed flask, with a peristaltic pump (Vapourtec SF-10) used to circulate the mixture around the system to an InsightMR flow tube (Bruker) located within the spectrometer (Bruker 500 MHz Avance III Ultrashield equipped with a nitrogen cooled Prodigy 5 mm broadband observed (BBO) Prodigy CryoProbe). The flow tube was connected to the reaction flask using narrow diameter polyetheretherketone (PEEK) tubing (0.762 mm i.d., Upchurch Scientific) using standard rubber seals to connect the tubing to the flask. All other connections were made using standard HPLC-type PEEK connectors (Upchurch Scientific), allowing the apparatus to be purged with inert or reactive gases as required. For further details, see reference [5].

The total volume of the flow apparatus was 3.7 mL, and the volume of the NMR flow cell was approximately 0.5 mL, corresponding to a mean residence time within the detection region of 8 s at a flow rate of 4 $mLmin^{-1}$.[5]

Data acquisition was performed without lock and with shimming performed using automated ^{1}H shimming routines, followed by manual fine tuning. Data processing was performed using commercially available software.

General procedure

The FlowNMR apparatus was purged with dry argon for 30 min to remove any traces or air or moisture prior to use. The apparatus was filled with 7.53 mL of a stock solution of potassium hydroxide (anhydrous, 0.112 g, 2 mmol) and 1,3,5-trimethoxybenzene (3.364 g, 0.02 mol) in dry, degassed isopropanol (200 mL).

The flow tube was then inserted into the spectrometer and automated shimming and tuning routines were performed. Frequency lock was switched off when using non-deuterated solvents, and shimming performed on proton peaks.

With the sample flowing, data acquisition was started using an automated kinetic routine or dedicated reaction monitoring software, with spectra recorded at specified time intervals. A concentrated solution of the catalyst in 2 mL of the stock solution was added. Spectra were acquired for 15 min to allow initial catalyst speciation to be observed. To start the reaction, acetophenone (0.47 mL, 4 mmol) was added.

Unless specified, the following parameters were used for the acquisition of all NMR kinetic data:

^{1}H selective excitation and ^{1}H (non-selective) spectra acquisition were interleaved with selective excitation spectra acquired every 30 s and non-selective spectra acquired every 60 s.

^{1}H (without selective excitation)

^{1}H spectra for the determination 1-phenylethanol, acetophenone, acetone and 1,3,5-trimethoxybenzene concentration were acquired using a standard 30° pulse sequence, with a 1.64 s acquisition time and 1 s relaxation delay time, using a single transient.

^{1}H selective excitation

^{1}H selective excitation spectra for the determination of the concentration of catalyst species **3** were acquired using a 1D double spin echo pulse sequence with gradient refocusing. A Q3 gaussian 180° pulse with a pulse length of 2272 µs (approximately 3 ppm width), centred on -5.5 ppm was used for selective refocusing with a 200 µs gradient recovery time. 8 transients were acquired with a 2 s acquisition time and 1 s relaxation delay time.

At the end of the reaction, additional spectra were recorded with and without flow, and correction factors were calculated for the intermediate or product peaks, which were applied to each spectrum to give the final peak areas for calculation of species concentration and plotting of kinetic data. (I = peak integral, CF = correction factor).[5]

$$I_{Corrected} = CF \times I$$

$$CF = \frac{I_{Static}}{I_{Flow}}$$

Concentrations of species were determined by peak integrals and referenced to 1,3,5-trimethoxybenzene internal standard.

Receiver Gain calibration

Since selective excitation experiments are recorded at significantly higher receiver gain than standard proton spectra, it is necessary to determine a compensation factor to allow quantitative comparison between peaks on the two different spectra.[6]

Relative integral areas of the methyl peak of 1,3,5-trimethoxybenzene (0.1 M) in a standard reaction mixture sample in isopropanol (see General Procedure above) were recorded at a range of receiver gains, resulting in a calibration factor of 0.9896 to transform integral values recorded at different receiver gains.

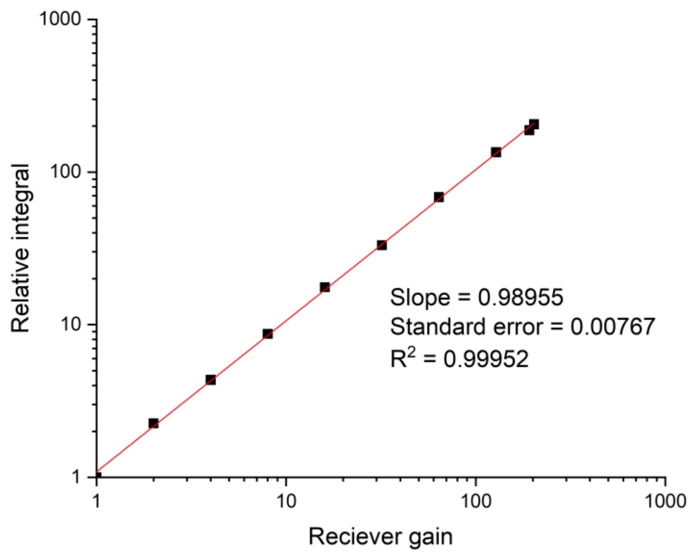

Figure S3: Receiver gain calibration for Bruker 500 MHz Avance III Ultrashield spectrometer equipped with a 5 mm broadband observed (BBO) Prodigy CryoProbe. 0.1 M 1,3,5-trimethoxybenzene in isopropanol reaction sample. Selective excitation using spin echo shaped pulse sequence (8 scans, 2 s acquisition time, 1 s relaxation delay time, 1600 µs Gaussian excitation peak, 20°C).

In order to allow comparison between the integral of the hydride peak recorded using selective excitation and the 1,3,5-trimethoxybenzene concentration reference, the integral of the hydride peak is reduced to give a Reduced Integral Value (RIV) according to the following equation:

$$RIV = \frac{I_{hydride} \times RG_{normal} \times 0.9896}{RG_{hydride}}$$

Where $I_{hydride}$ = Integral area of hydride peak, RG_{normal} = receiver gain for selective excitation experiment of internal standard (e.g. TMB), $RG_{hydride}$ = receiver gain for selective excitation experiment of hydride.

The concentration of the hydride species can then be calculated by comparison to a selective excitation experiment of the internal reference (such as 1,3,5-trimethoxybenzene (TMB)) of known concentration:

$$[Hydride] = RIV \times \frac{[TMB]}{I_{TMB}/Number\ of\ protons\ associated\ with\ TMB\ peak}$$

This calculation process was tested on a standard sample containing known concentrations of acetophenone and 1,3,5-trimethoxybenzene in isopropanol solvent and the accuracy of component concentrations calculated with this method compared to actual concentrations of components was found to have an error 3.6%.

Observation of hydrides under reaction conditions (Figure 1)

The reaction was carried out as described above, using 400 mM acetophenone with a 2 mM concentration of complex **1**.

Reaction with achiral Ts(ethylenediamine) ligand (Figure S1)

The reaction was carried out as described above, using 400 mM acetophenone with a 2 mM concentration of RuCl(Ts(ethylenediamine))(mesitylene). A conversion of 83% of the acetophenone to *rac*-1-phenylethanol was observed after 5 h.

Deactivation under reaction conditions (Figure 2)

The reaction was carried out as described above with a 4 mM concentration of complex **1**, however a mixture of 40 mM acetophenone, 360 mM *rac*-1-phenylethanol and 360 mM acetone was added at the start of the experiment to simulate the concentration of substrate and product at the reaction equilibrium.

Tethered catalyst (Figure 4)

The reaction was carried out as described above with a 4 mM concentration of complex **1**, 400 mM acetophenone and 20 mM KOH. An additional 400 mM of acetophenone was added after 17 h. ^{1}H spectra were acquired every 300 s using the parameters described above. ^{1}H selective excitation experiments were acquired every 300 s with 96 transients. All other acquisition parameters were as described above.

Online monitoring with HPLC (Figure 4)

Online monitoring with HPLC was performed using an automated sample collection valve positioned on the flow loop after the NMR flow probe as described in (Berry 2019).Berry, et al. [7] HPLC aliquots were sampled every 30 min for the duration of the experiment. Analysis was performed using an Agilent 1260 Infinity II LC equipped with a Chiracell OD-H column (Daicell chiral, 250 mm length, 4.6 mm diameter, 5 µm particle size), with the sample eluted with a 9:1 hexane/IPA mixture at 1 mL/min. *R*-1-phenylethanol was observed to elute at 7.56 min and *S*-1-phenylethanol at 8.15 min.

Enantiomeric excess was calculated using the formula:

$$\%ee = \frac{[R] - [S]}{[R] + [S]} \times 100$$

Conventional NMR experiments

Deactivation in absence of substrate (Figure S2)

The reaction was carried out off-line using a standard valved NMR tube. A mixture of 6 mM complex **1** and 12 mM NaO^{i}Pr were dissolved in 0.5 mL C_6D_6 under argon atmosphere, forming a purple solution of active catalyst **2**. 20 µL isopropanol was added, leading to reaction to form hydride species **3**. NMR kinetic experiments were then performed as described above.

Reaction with CO_2 (Figure 3)

Sodium isopropoxide (2.0 mg, 0.025 mmol) and [(mesitylene)RuCl(R,R)-(TsDPEN)] (10 mg, 0.016 mmol) were dissolved in THF-d_8 (0.5 mL) to give a deep purple solution of **2**. The mixture was stirred for 5 min at room temperature and passed through a 0.2 µm PTFE syringe filter. The solution was charged to a pressure resistant NMR tube and iso-propanol (8 µL, 0.104 mmol) was added under inert atmosphere yielding a yellow brown solution of hydrides **3a** and **3b**. The sample was pressurised with 5 bar CO_2 and upon mixing the solution turned bright yellow indicative of the formation of the formate complex.

Diffusion measurements

Sodium isopropoxide (2.0 mg, 0.025 mmol) and [(mesitylene)RuCl(*R,R*)-(TsDPEN)] (5 mg, 0.008 mmol) were dissolved in either isopropanol-h_8 or C_6D_6 (0.5 mL) in a standard valved NMR tube. For the sample in C_6D_6, isopropanol-h_8 (approx. 10 µL) was added. The samples were left for 24 h before acquiring the spectra to allow the concentrations of hydrides **3a** and **3b** to reach equilibrium.

DOSY measurements were recorded using a *pseudo*-2D stimulated echo pulse sequence. Bipolar gradients with magnitudes between 5.9 and 53.1 G cm^{-1} and 1 ms duration (δ/2) were used for diffusion measurement, with 0.06 s diffusion time (Δ). Two spoil gradients of -10.1 and -7.8 G cm^{-1} with 0.6 ms duration were employed. All spectra were recorded as an average of 32 transients, with a relaxation delay of 3 s.

DOSY data was processed using Topspin 4.0.7 and Dynamic Center 2.5.6 with 0.3 Hz exponential line broadening. Fitting was performed using the 'NonLinearModelFit' function in Mathematica 11.3.0.0 with the function:

$$A * e^{-D(\gamma\delta)^2(\%_g*\mathrm{gmax})^2(\Delta-\delta/3)}$$

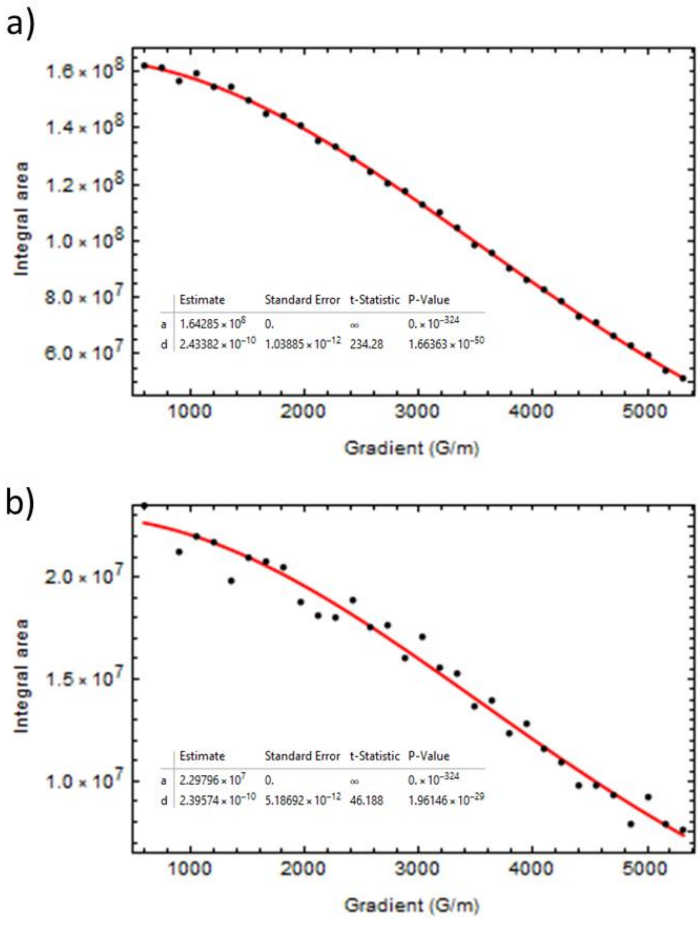

*Figure S4: DOSY fitting plots for hydride species **3a** and **3b** in isopropanol-h_8.*

a)

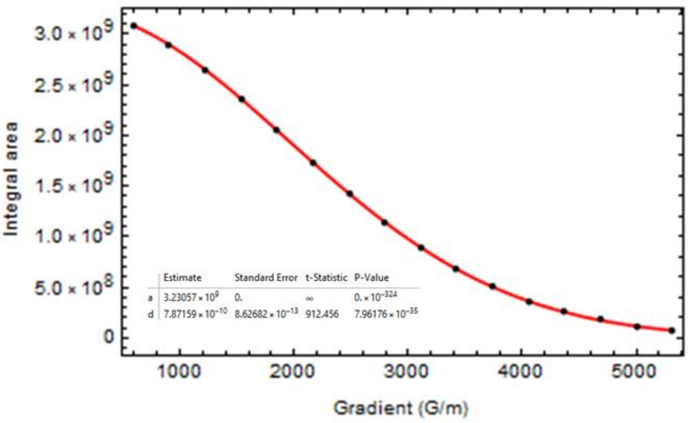

b)

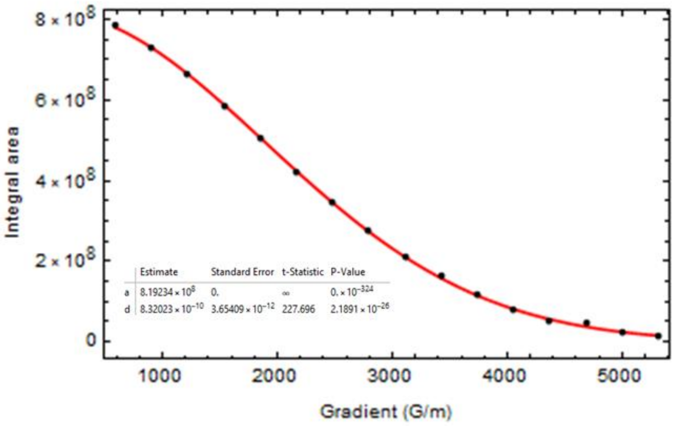

*Figure S5: DOSY fitting plots for hydride species **3a** and **3b** in C_6D_6.*

T_1 measurements

T_1 measurements were performed on the same sample of **3a** and **3b** in isopropanol-h_8 used for diffusion measurements.

Pseudo-2D spectra were recorded using an inversion recovery pulse sequence with 16 variable delays between 0.001 and 4.5 s. All spectra were acquired with 8 transients, with a relaxation delay of 15 s.

T_1 data was processed using Topspin 4.0.7 and Dynamic Center 2.5.6 with 0.3 Hz exponential line broadening. Fitting was performed using the 'NonLinearModelFit' function in Mathematica 11.3.0.0 with the function:

$$I_t = I_0\left(1 - A\, e^{-\frac{t}{T_1}}\right)$$

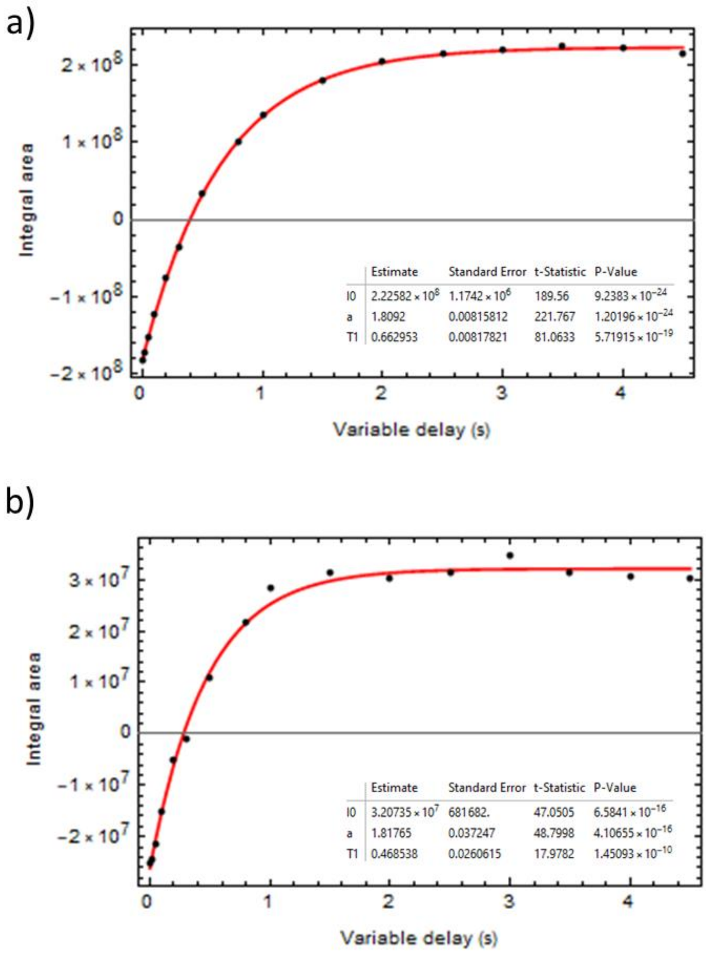

*Figure S6: T_1 fitting plots for hydride species **3a** and **3b** in isopropanol-h_8.*

Nuclear Overhauser Effect (NOE) spectra

*Non-tethered catalyst **3** (Figures S7 & S8)*

The experiment was carried out off-line using a standard valved NMR tube. A mixture of complex **1** (12 mg, 19.3 mM) and NaO^iPr (2 mg, 24.4 mM) were dissolved in 1 mL C_6D_6 under argon atmosphere, forming a purple solution of active catalyst **2**. 10 µL isopropanol was added, leading to reaction to form hydride species **3a**. The sample was filtered through a 0.2 µm syringe filter and left for 24 h before acquiring the spectra to allow the concentrations of hydrides **3a** and **3b** to reach equilibrium.

^1H spectra were acquired with 16 transients, 2.18 s acquisition time and 1 s relaxation delay time. Spectra were processed with 0.3 Hz exponential line broadening. Selective NOE spectra were acquired using a gradient spin echo pulse sequence with a shaped 180° pulse centred at either -4.88 or -6.59 ppm. 2048 transients were acquired with 2.18 s acquisition time, 2 s relaxation delay time and using a 1600 µs Gaussian shaped pulse. Spectra were processed with 0.1 Hz exponential line broadening.

*Tethered catalyst **6** (Figures S23 & S24)*

Complex **4** (24.8 mg, 66.7 mM) and NaO^iPr (4.8 mg, 97.5 mM) were dissolved in dissolved in 35 mL propan-2-ol under argon atmosphere, forming a pale purple mixture. The solvent was removed under vacuum to isolate a brown solid which was redissolved in 0.6 mL C_6D_6 to give a purple solution containing a mixture of **5** and **6**. The sample was filtered through a 0.2 µm syringe filter. ^1H NMR spectra were acquired immediately after preparation and again after 24 h after equilibrium of hydrides **6a** and **6b** was reached.

^1H spectra were acquired with 64 transients, 1.64 s acquisition time and 1 s relaxation delay time. Spectra were processed with 0.3 Hz exponential line broadening. Selective NOE spectra were acquired using a gradient spin echo pulse sequence with a shaped 180° pulse centred at either -4.539 or -5.26 ppm. 10240 transients were acquired with 2.18 s acquisition time, 2 s relaxation delay time and using a 80 ms Gaussian shaped pulse. Spectra were processed with 2.0 Hz exponential line broadening.

NMR assignment and NOE experiments

Primary NMR data files are available to download from DOI: 10.6084/m9.figshare.14473035.

Hydrides 3a and 3b

The ^{1}H and ^{13}C chemical shifts, integrals and J-couplings for all major peaks and those minor peaks which could be assigned confidently from NOE, homonuclear- and heteronuclear-correlation experiments are detailed in Table S1 below. Due to overlapping peaks within the aromatic region, the phenyl environments were not explicitly assigned. A number of small peaks (<5% intensity of major catalyst species) were observed in the ^{1}H NMR spectrum but were not assigned and may correspond to decomposition products or other minor species. The presence of 16 e^- complex **2** is expected due to the reversibility of the reaction with *iso*-propanol.

Accurate assignment of the backbone CH protons is crucial for the correct assignment of stereochemistry by NOE interactions. ^{1}H-^{1}H COSY and ^{1}H-^{13}C HMBC spectra showed that the peak at 4.02 ppm had correlations to the amine protons that were not present for the peak at 4.14 ppm, indicating that this is the backbone CH closest to the amine group. This was confirmed by a correlation from the peak at 4.14 ppm to the tosyl C-SO_2 carbon which was not present for the backbone CH at 4.02 ppm. The $^{3}J_{CH\text{-}NH}$ coupling was also seen in the ^{1}H spectra of the backbone CH peak at 4.02 ppm and the NH peak at 4.55 ppm (Figure S9).

The low intensity and broad line widths of the ligand backbone peaks for complex **3b** makes assignment challenging, however $^{2}J_{NH\text{-}NH}$ coupling is clearly observed in the COSY spectrum between the peaks at 1.76 and 3.13 ppm along with $^{3}J_{CH\text{-}NH}$ coupling between the peaks at 3.13 and 3.24 ppm. The absence of $^{1}J_{C\text{-}H}$ coupling in the HSQC spectrum for the peaks at 1.76 and 3.13 ppm indicates the assignment of these peaks as NHs. The peak at 3.24 ppm must therefore be the backbone CH-NH_2. A weak $^{3}J_{CH\text{-}CH}$ coupling is observed between the peaks at 3.24 and 5.33 ppm, indicating the later peak to be the backbone CH-NTs.

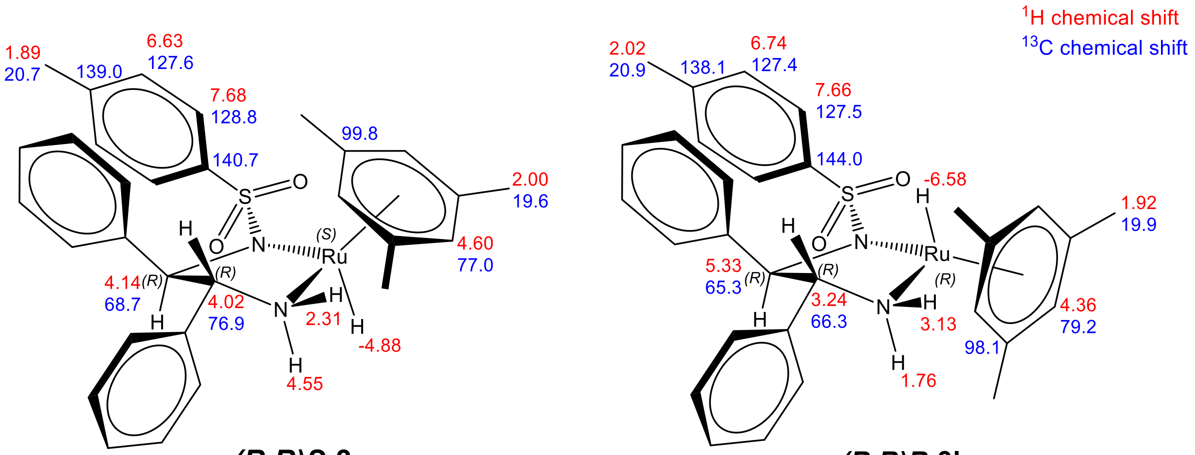

(R,R)S-3a **(R,R)R-3b**

*Table S1: Summary of chemical shifts, assignments, multiplicity, J-coupling and 2D correlations for a mixture of hydrides **3a** and **3b**. The presence of complex **2** is due to the equilibrium between **2** and **3** resulting in incomplete formation of the hydride species. Notes: [a] Overlaps with C_6D_5H peak, [b] Weak, [c] Estimated integral due to overlapping peaks, [d] No ^1H-^{13}C correlation observed in HSQC.*

#	Environment	Species	δ ^{1}H (ppm)	Multi-plicity	J_{HH} (Hz)	Integral	Number of ^{1}H	Integral/ # Protons	δ ^{13}C (ppm)	^{1}H-^{13}C HMBC correlations	^{1}H-^{1}H COSY correlations	Diffusion constant (x10^{-10} m^2/s)
1	Ru-H	***(R,R)R*-3b**	-6.59	s	-	0.22	1	0.22	-	-	-	
2	Ru-H	***(R,R)S*-3a**	-4.88	s	-	1	1	1	-	-	-	
3	*Silicone grease*	*Imp.*	*0.29*	*s*	*-*	*1.4*	*-*		*1.0*	*-*	*-*	*1.41*
4	*Isopropanol-CH_3*	*IPA*	*0.99*	*d*	*6.1*	*195.44*	*6*	*32.57*	*25.1*	*#20*	*#20*	*25.7*
5	*Isopropanol-OH*	*IPA*	*1.21*	*d (br)*	*3.5*	*36.94*	*1*	*36.94*	*-*	*#4, 20*	*#20*	*25.8*
6	*Acetone-CH_3*	*Ac.*	*1.55*	*s*	*-*	*5.41*	*6*	*0.90*	*29.7*	*#P*	*-*	*28.9*
7	Mesitylene-CH_3		1.74	s	-	0.36	9	0.04	18.5	#26	#26	7.29
8	NHH (anti to Ru-H)	***(R,R)R*-3b**	1.76	d (br)	10.0	0.23	1	0.23	-	-	#17, 30	7.77
9	Tosyl-CH_3	***(R,R)S*-3a**	1.89	s	-	2.93	3	0.98	20.7	#36[a], 45[b], E, J[b]	#32, 45	7.49
10	Mesitylene-CH_3	***(R,R)R*-3b**	1.92	s	-	2.14	9	0.24	19.9	#25, A	#25	7.82

#	Environment	Species	δ ^{1}H (ppm)	Multi-plicity	J_{HH} (Hz)	Integral	Number of ^{1}H	Integral/ # Protons	δ ^{13}C (ppm)	^{1}H-^{13}C HMBC correlations	^{1}H-^{1}H COSY correlations	Diffusion constant (x10^{-10} m^{2}/s)
**11**	Tosyl-CH_3	**(R,R)-2**	1.94	s	-	1.93	3	0.64	20.8	#36[a], F, H[b]	#36, 46	7.09
**12**	Mesitylene-CH_3	***(R,R)S*-3a**	2.00	s	-	8.61	9	0.96	19.6	#28, C	#28	7.51
**13**	Tosyl-CH_3	***(R,R)R*-3b**	2.02	s	-	0.77	3	0.26	20.9	#33[a], D	#33, 44	7.79
**14**	Mesitylene-CH_3	**(R,R)-2**	2.09	s	-	5.24	9	0.58	18.7	#29, B	#29	7.10
15	*Toluene CH_3*	*Imp.*	*2.16*	*s*	*-*	*0.26*	*3*	*0.09*	*-*	*-*	*-*	*18.0*
**16**	NHH (anti to Ru-H)	***(R,R)S*-3a**	2.31	d (br)	9.6	0.92	1	0.92	-	-	#22, 27	7.48
**17**	NHH (syn to Ru-H)	***(R,R)R*-3b**	3.13	d (br)	11.0	0.23	1	0.23	-	-	#8, 18	7.75
**18**	CH-NH_2	***(R,R)R*-3b**	3.24	s (br)	-	0.23	1	0.23	66.3	-	#17, 30	7.75
**19**	CH-NH	**(R,R)-2**	3.63	ddd	13.0, 10.2, 2.4	0.77	1	0.77	75.1	-	#21, 23	7.35
20	*Isopropanol-CH*	*IPA*	*3.72*	*d, sept*	*6.0, 3.8*	*32.97*	*1*	*32.97*	*63.5*	*#4*	*#4, 5*	*25.6*
**21**	CH-NTs	**(R,R)-2**	3.91	d	10.6	0.78	1	0.78	69.7	#19, 38, G[b], L[b]	#19, 23	7.11
**22**	CH-NH_2	***(R,R)S*-3a**	4.02	ddd	12.9, 10.2, 2.3	1.15	1	1.15	76.9	#24	#16, 24, 27	7.46
**23**	NH	**(R,R)-2**	4.11	d (br)	10.1	0.50[c]	1	0.50	-	-	#19	6.99
**24**	CH-NTs	***(R,R)S*-3a**	4.12	d	10.2	1.13	1	1.13	68.7	#22, 37[a], M, K	#22	7.47
**25**	Mesitylene-CH	***(R,R)R*-3b**	4.36	s	-	0.65	3	0.22	79.2	#10, A	#10	7.82
**26**	Mesitylene-CH		4.45	s	-	0.12	3	0.04	79.4	#7	#7	7.25
**27**	NHH (syn to Ru-H)	***(R,R)S*-3a**	4.55	t (br)	11.2	0.99	1	0.99	-	-	#16, 22	7.38
**28**	Mesitylene-CH	***(R,R)S*-3a**	4.60	s	-	2.92	3	0.97	77.0	#12, C	#12	7.50
**29**	Mesitylene-CH	**(R,R)-2**	4.83	s	-	1.68	3	0.56	78.4	#14, B	#14	7.06
**30**	CH-NTs	***(R,R)R*-3b**	5.33	s	-	0.22	1	0.22	65.3	#39[a]	#8, 18	7.76
**31**	Phenyl-CH	**(R,R)-2**	6.59	d	7.7	1.27	2	0.635	126.9	#19[b], 36[a]	#34	7.02

#	Environment	Species	δ ^{1}H (ppm)	Multi-plicity	J_{HH} (Hz)	Integral	Number of ^{1}H	Integral/ # Protons	δ ^{13}C (ppm)	^{1}H-^{13}C HMBC correlations	^{1}H-^{1}H COSY correlations	Diffusion constant (x10^{-10} m^2/s)
**32**	Tosyl-CH (meta)	***(R,R)S*-3a**	6.63	d	8.0	2.10	2	1.05	127.6[a]	#9, J	#9, 45	7.45
**33**	Tosyl-CH (meta)	***(R,R)R*-3b**	6.74	d	8.0	0.36[c]	2	0.18	127.4[a]	#N	#44	7.59
**34**	Phenyl-CH	**(R,R)-2**	6.76	t	7.6	1.57	2	0.79	128.4	#31, G	#31, 39	7.02
**35**	Phenyl-CH	***(R,R)S*-3a, *(R,R)R*-3b**	6.80 to 6.86	m	-	4.26	4	1.07	125.6, 127.0	#37[a], M	#37	7.48
**36**	Tosyl-CH (meta)	**(R,R)-2**	6.88	d	7.8	1.52	2	0.76	127.8[a]	#11, H	#11, 46	7.07
**37**	Phenyl-CH	***(R,R)S*-3a**	6.90 to 6.98	m	-	7.28	6	1.21	127.1, 127.8[a], 128.3	#24, 22, 35, K	#35	7.47
**38**	Phenyl-CH	**(R,R)-2**	6.99 to 7.08	m	-	3.47	4	0.87	126.7, 127.1, 129.1	#21[b], L	-	7.07
**39**	Phenyl-CH	***(R,R)R*-3b**	7.11	d		0.67	2	0.34	127.6[a]	-	#35	7.78
**40**	Phenyl-CH	***(R,R)R*-3b**	7.12	t	7.5	0.49	2	0.25	-[d]	-	#42, 43[b]	7.97
41	*C_6D_5H*	*Sol.*	*7.16*	*s*	*-*	*17.86*	*1*	*17.86*	*127.7*	*-*	*-*	*23.1*
**42**	Phenyl-CH	***(R,R)R*-3b**	7.23	t	7.8	0.43	2	0.22	128.4	#H[b]	#40, 4	7.74
**43**	Phenyl-CH	***(R,R)R*-3b**	7.62	d	7.8	0.46	2	0.23	128.5	#37[a]	#42	7.75
**44**	Tosyl-CH (ortho)	***(R,R)R*-3b**	7.66	d	8.1	0.56	2	0.28	127.5[a]	#D	#13[a], 33	7.71
**45**	Tosyl-CH (ortho)	***(R,R)S*-3a**	7.68	d	8.1	1.88	2	0.94	128.8	#E	#9, 32	7.50
**46**	Tosyl-CH (ortho)	**(R,R)-2**	8.11	d	8.2	1.13	2	0.57	130.0	#G, H[b]	#11, 36	7.05
**A**	Mesitylene-C-CH_3	***(R,R)R*-3b**	-	-	-	-	-	-	98.1	-	-	-
**B**	Mesitylene-C-CH_3	**(R,R)-2**	-	-	-	-	-	-	99.6	-	-	-
**C**	Mesitylene-C-CH_3	***(R,R)S*-3a**	-	-	-	-	-	-	99.8	-	-	-
**D**	Tosyl-C-CH_3	***(R,R)R*-3b**	-	-	-	-	-	-	138.1	-	-	-

#	Environment	Species	δ ^{1}H (ppm)	Multi-plicity	J_{HH} (Hz)	Integral	Number of ^{1}H	Integral/ # Protons	δ ^{13}C (ppm)	^{1}H-^{13}C HMBC correlations	^{1}H-^{1}H COSY correlations	Diffusion constant ($x10^{-10}$ m^2/s)
**E**	Tosyl-C-CH_3	***(R,R)S*-3a**	-	-	-	-	-	-	139.0	-	-	-
**F**	Tosyl-C-CH_3	**(R,R)-2**	-	-	-	-	-	-	139.5	-	-	-
**G**	Phenyl-C-$(CH)_2$	**(R,R)-2**	-	-	-	-	-	-	139.7	-	-	-
**H**	Tosyl-C-SO_2	**(R,R)-2**	-	-	-	-	-	-	140.6	-	-	-
**J**	Tosyl-C-SO_2	***(R,R)S*-3a**	-	-	-	-	-	-	140.7	-	-	-
**K**	Phenyl-C-$(CH)_2$	***(R,R)S*-3a**	-	-	-	-	-	-	140.9	-	-	-
**L**	Phenyl-C-$(CH)_2$	**(R,R)-2**	-	-	-	-	-	-	141.7	-	-	-
**M**	Phenyl-C-$(CH)_2$	***(R,R)S*-3a**	-	-	-	-	-	-	143.5	-	-	-
**N**	Tosyl-C-SO_2	***(R,R)R*-3b**	-	-	-	-	-	-	144.0	-	-	-
P	*Acetone C=O*	*Ac.*	-	-	-	-	-	-	*203.8*	-	-	-

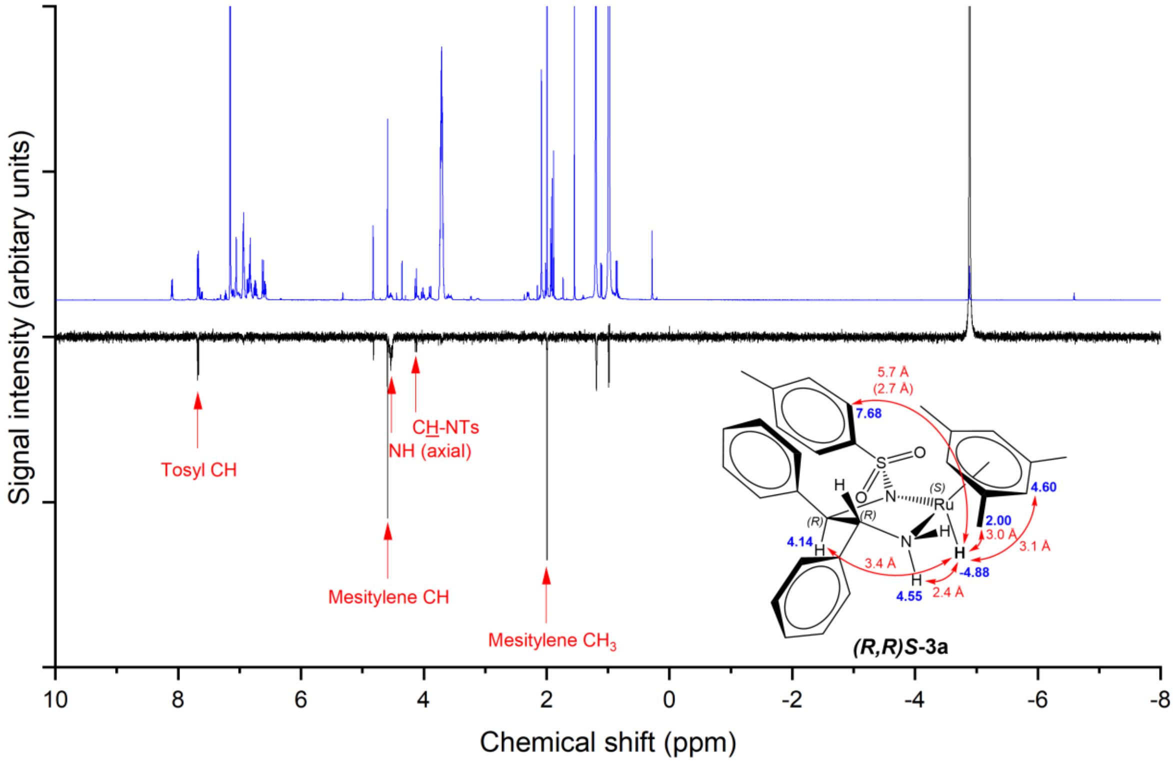

*Figure S7: ^{1}H and selective NOE NMR spectra of hydride **3a** (-4.88 ppm), showing NOE interactions of the hydride with other protons within the molecule, chemical shifts and assignment (19.3 mM (**1**), 24.4 mM NaO^{i}Pr, 0.5 mL C_6D_6, 10 µL isopropanol, 25 °C, Ar atmosphere, conventional NMR tube). ^{1}H acquisition (16 scans, 2.18 s acquisition time, 1 s relaxation delay time, spectra processed with 0.3 Hz exponential line broadening). Selective NOE using a gradient spin echo pulse sequence with a shaped 180° pulse centred at -4.88 ppm (2048 scans, 2.18 s acquisition time, 2 s relaxation delay time, 80 ms Gaussian shaped pulse; spectra processed with 0.1 Hz exponential line broadening).*

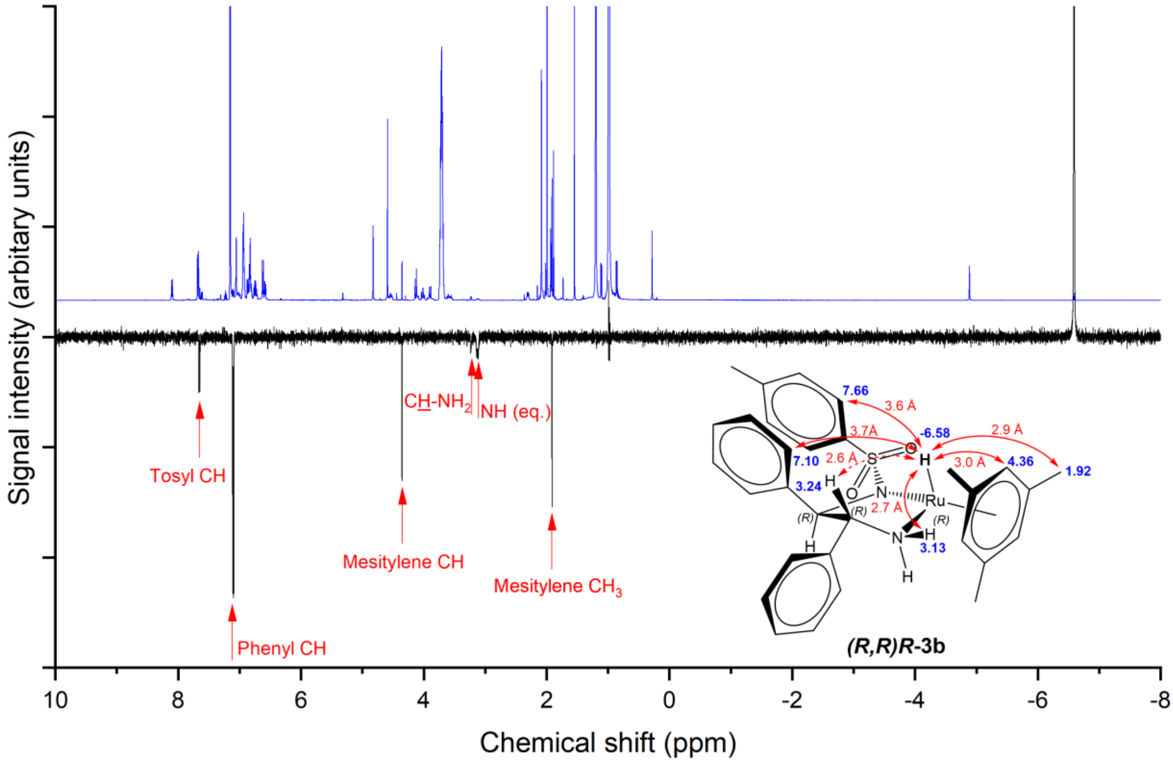

*Figure S8: ^{1}H and selective NOE NMR spectra of hydride **3b** (-6.59 ppm), showing NOE interactions of the hydride with other protons within the molecule, chemical shifts and assignment (19.3 mM (**1**), 24.4 mM NaO^{i}Pr, 0.5 mL C_6D_6, 10 µL isopropanol, 25 °C, Ar atmosphere, conventional NMR tube). ^{1}H acquisition (16 scans, 2.18 s acquisition time, 1 s relaxation delay time, spectra processed with 0.3 Hz exponential line broadening). Selective NOE using a gradient spin echo pulse sequence with a shaped 180° pulse centred at -6.59 ppm (2048 scans, 2.18 s acquisition time, 2 s relaxation delay time, 80 ms Gaussian shaped pulse; spectra processed with 0.1 Hz exponential line broadening).*

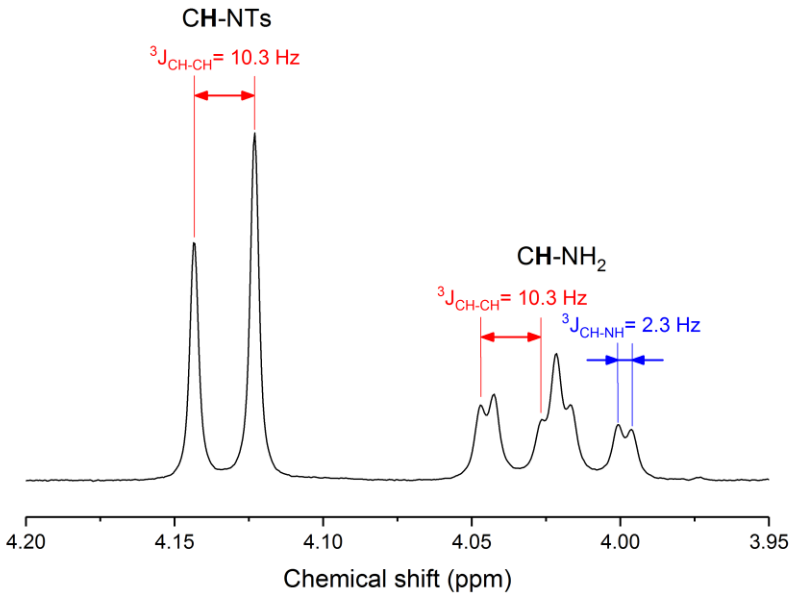

*Figure S9: ^{1}H NMR spectrum of hydride complex **3a** showing ligand backbone CH peaks with $^3J_{CH\text{-}CH}$ and $^3J_{CH\text{-}NH}$ coupling values indicated.*

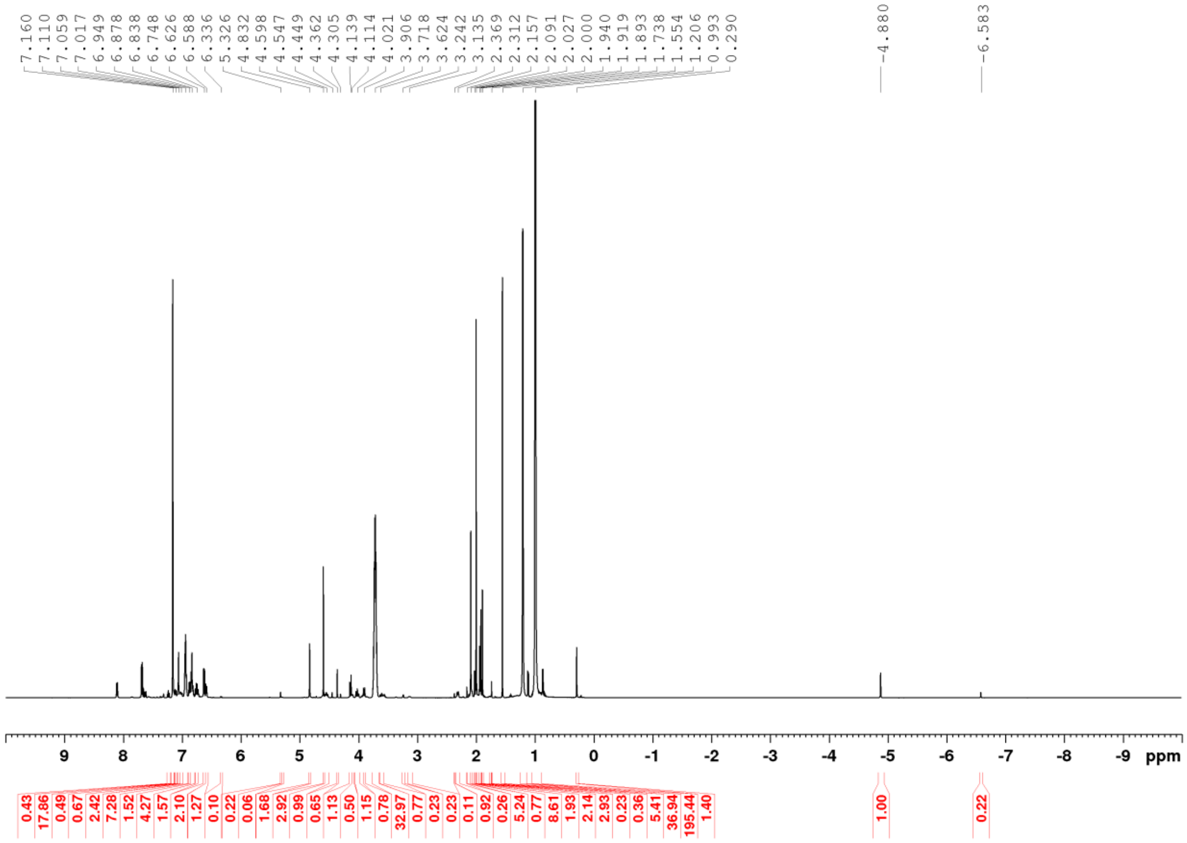

*Figure S10: ^{1}H NMR spectrum of a mixture of complexes **3a** and **3b**.*

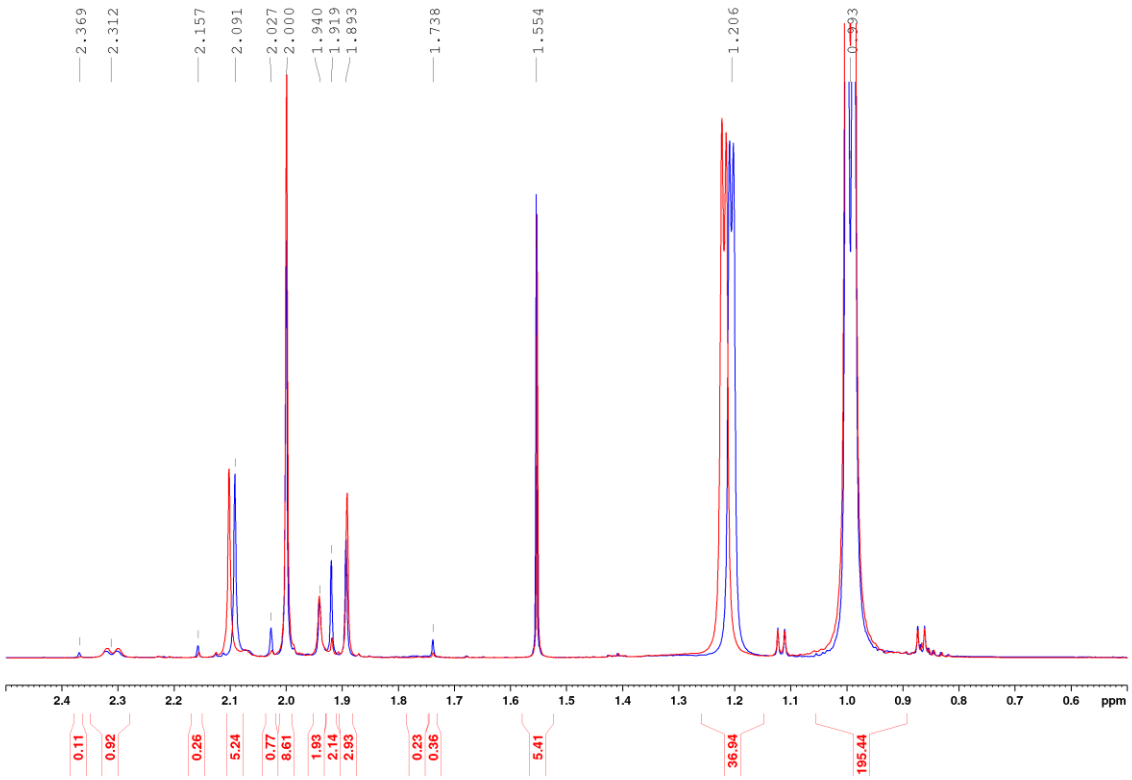

*Figure S11: Expansion of 0.5 – 2.5 ppm region of the ^{1}H NMR spectrum of a mixture of complexes **3a** and **3b**. Red = spectrum acquired immediately after adding IPA (majority **2** and **3a**), Blue = spectrum after 24h showing equilibrium mixture of **2**, **3a** and **3b**.*

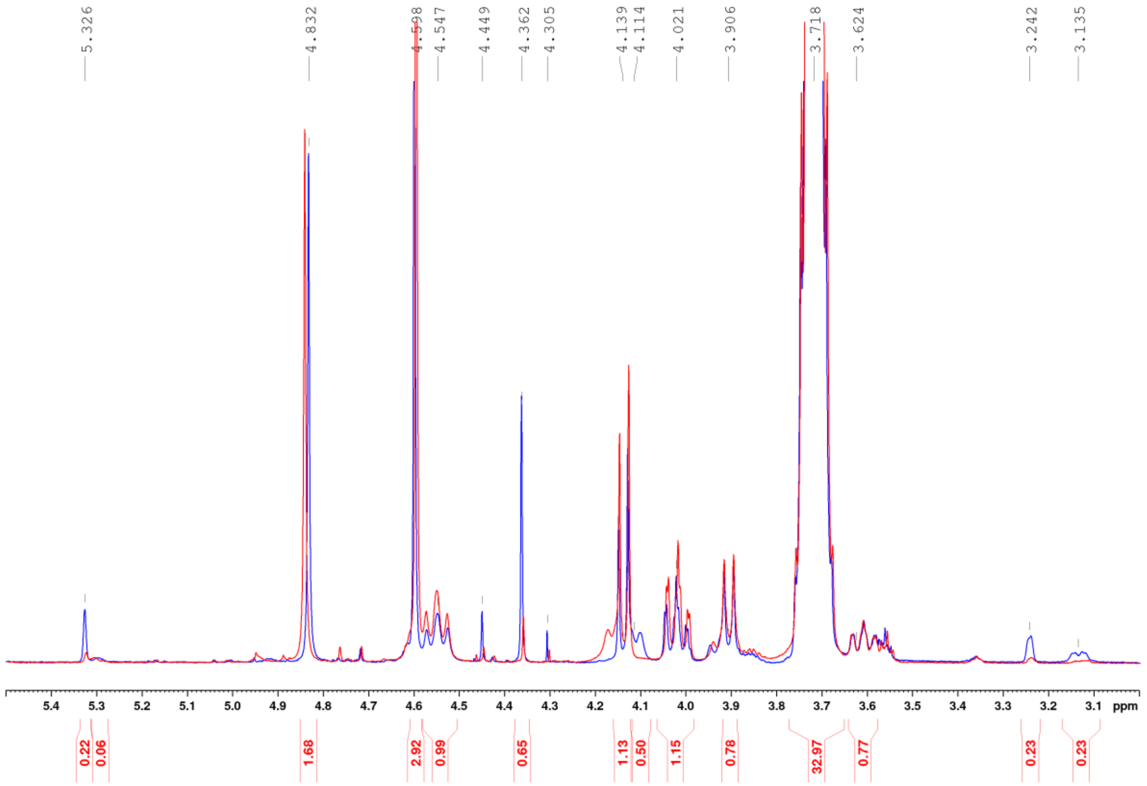

*Figure S12: Expansion of 3 – 5.5 ppm region of the ^{1}H NMR spectrum of a mixture of complexes **3a** and **3b**. Red = spectrum acquired immediately after adding IPA (majority **2** and **3a**), Blue = spectrum after 24h showing equilibrium mixture of **2**, **3a** and **3b**.*

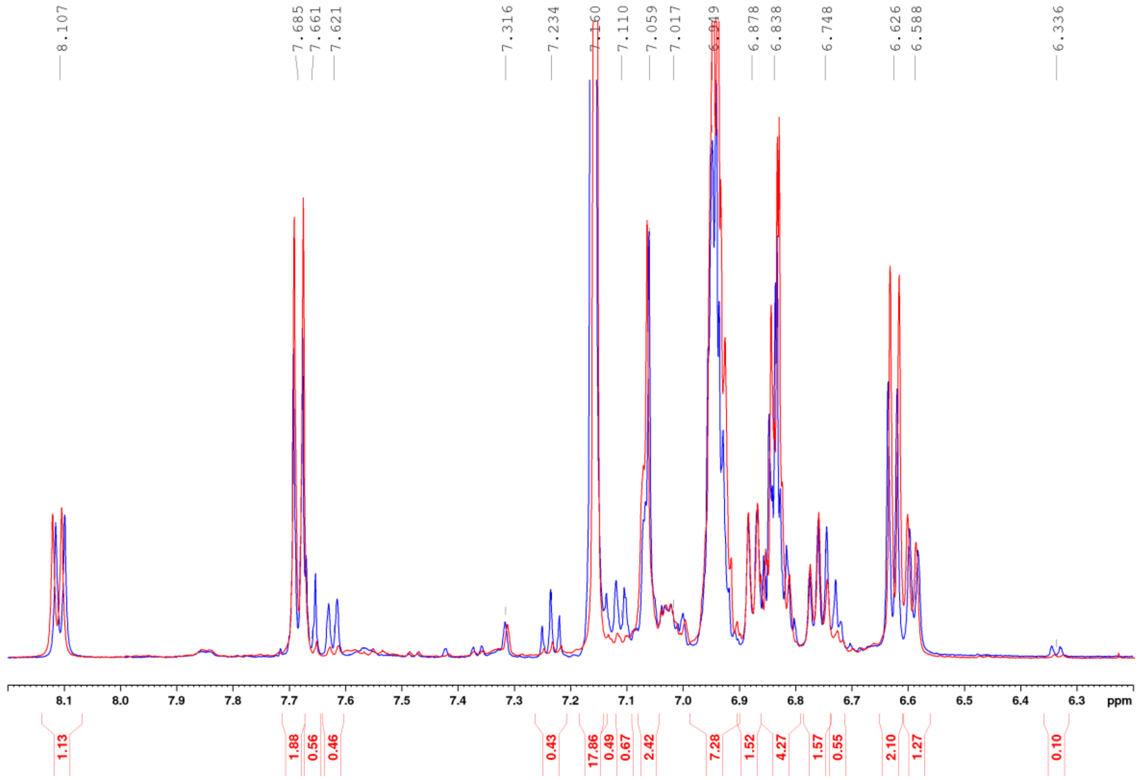

*Figure S13: Expansion of 6.2 – 8.2 ppm region of the ^{1}H NMR spectrum of a mixture of complexes **3a** and **3b**. Red = spectrum acquired immediately after adding IPA (majority **2** and **3a**), Blue = spectrum after 24h showing equilibrium mixture of **2**, **3a** and **3b**.*

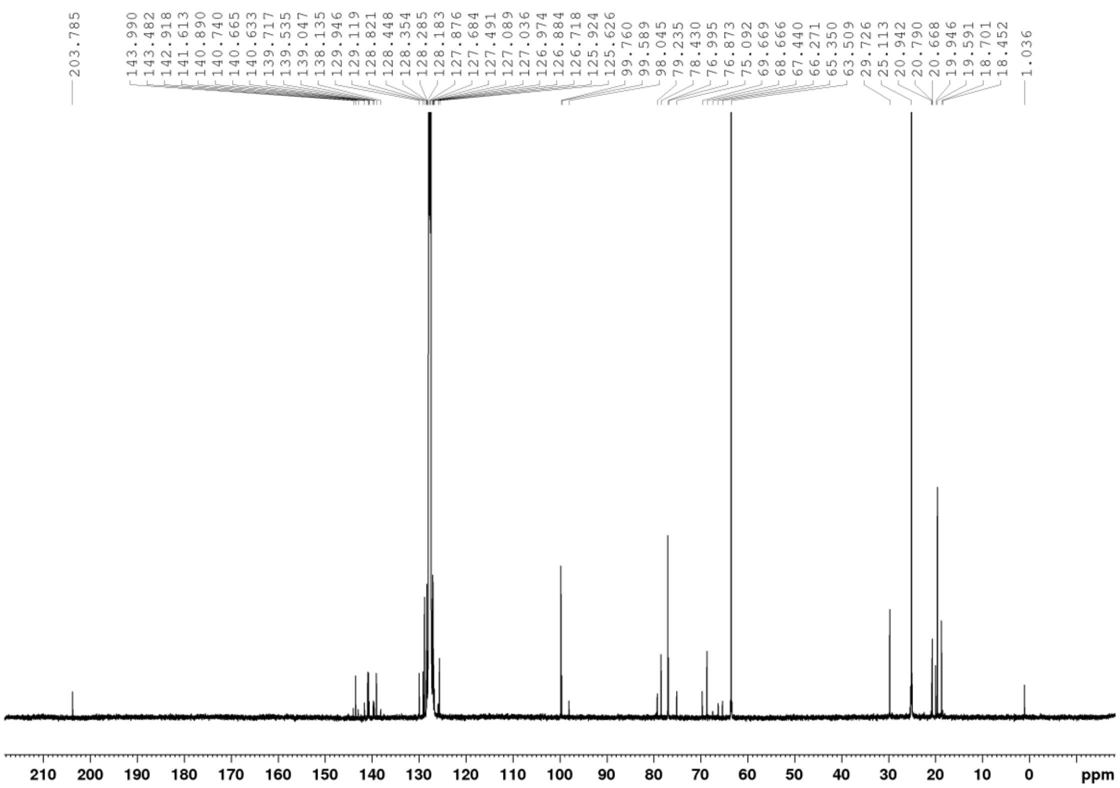

*Figure S14: ^{13}C NMR spectrum of a mixture of complexes **3a** and **3b**.*

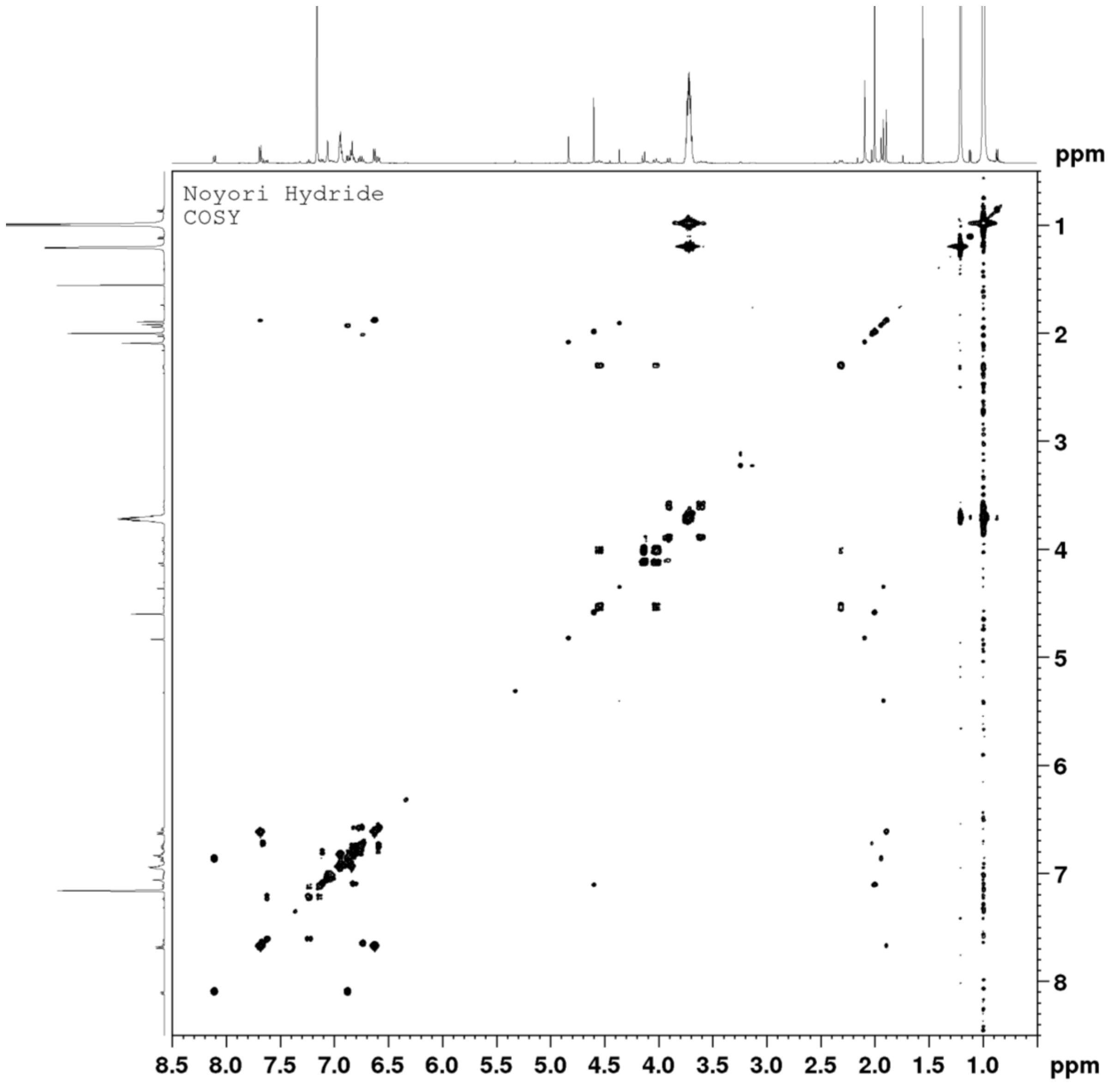

*Figure S15: ^1H-^1H COSY spectrum of a mixture of complexes **3a** and **3b**.*

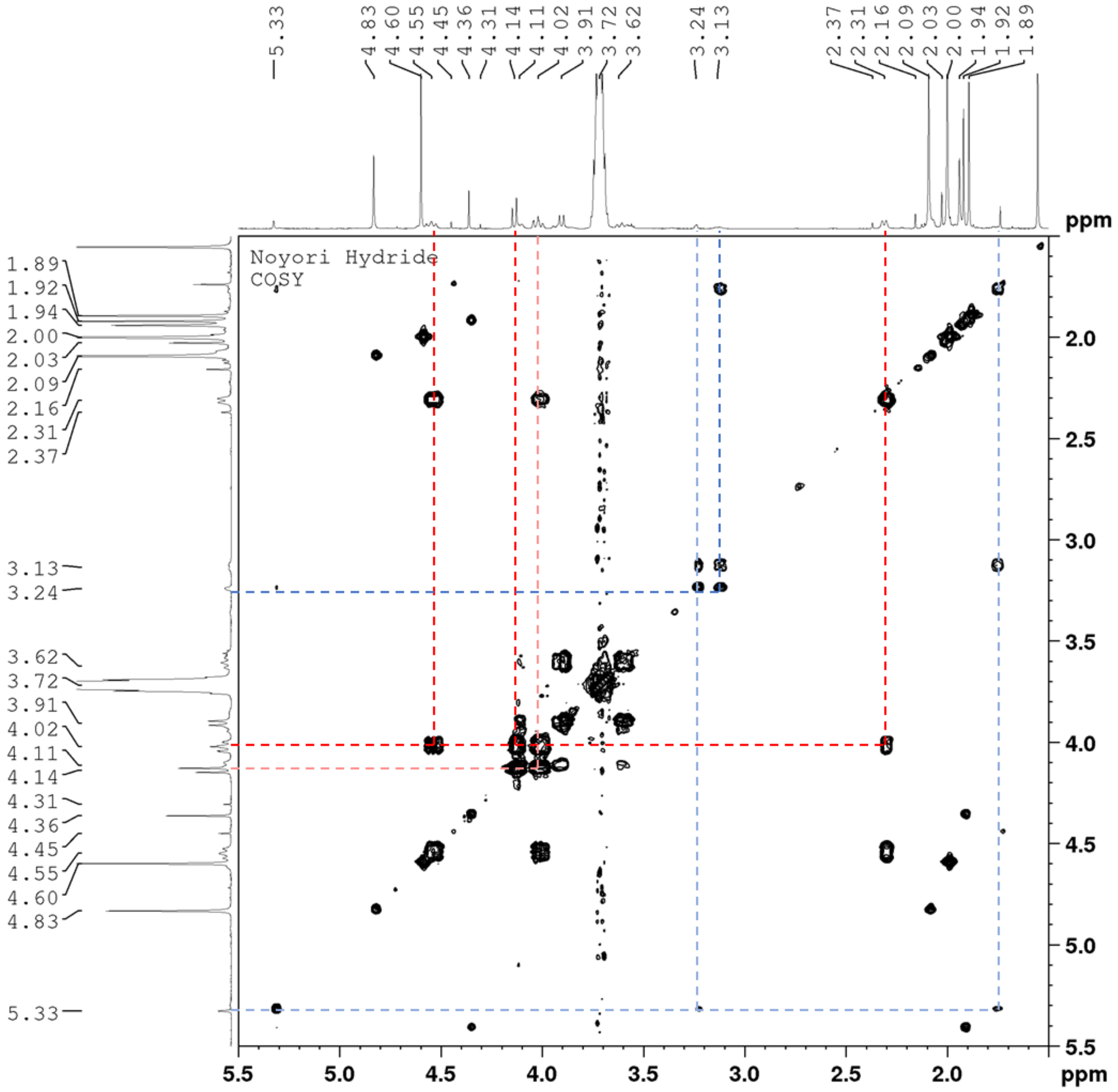

*Figure S16: Expansion of 1.5 – 5.5 ppm region of ^{1}H-^{1}H COSY spectrum of a mixture of complexes **3a** and **3b**, showing correlations between the backbone CH peaks at 4.02 and 4.14 ppm and the NH peaks at 2.31 and 4.55 ppm for **3a** (red) and backbone CH peaks at 5.33 and 3.24 ppm and the NH peaks at 3.13 and 1.76 ppm for **3b** (blue).*

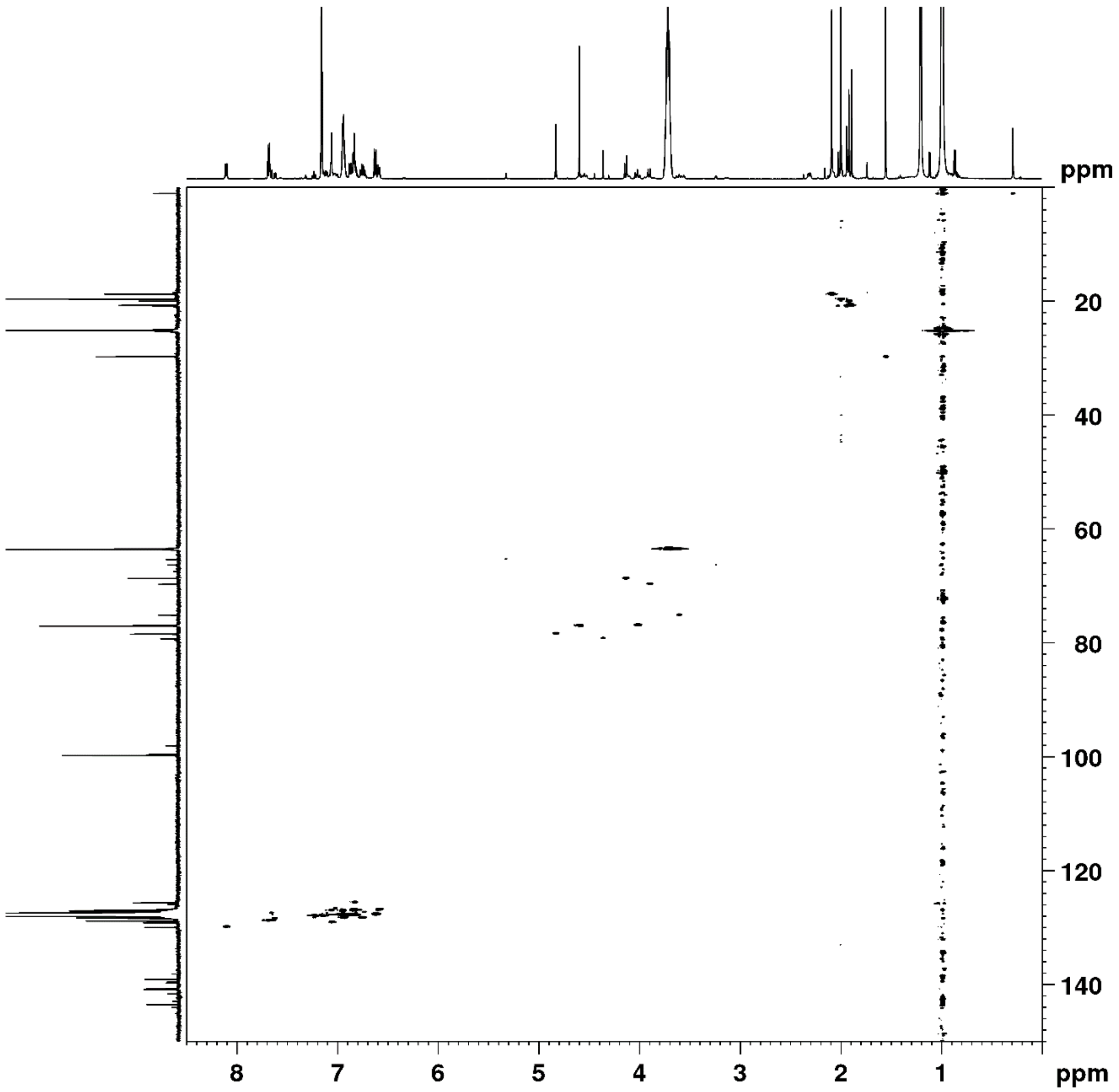

*Figure S17: ^{1}H-^{13}C HSQC spectrum of a mixture of complexes **3a** and **3b**.*

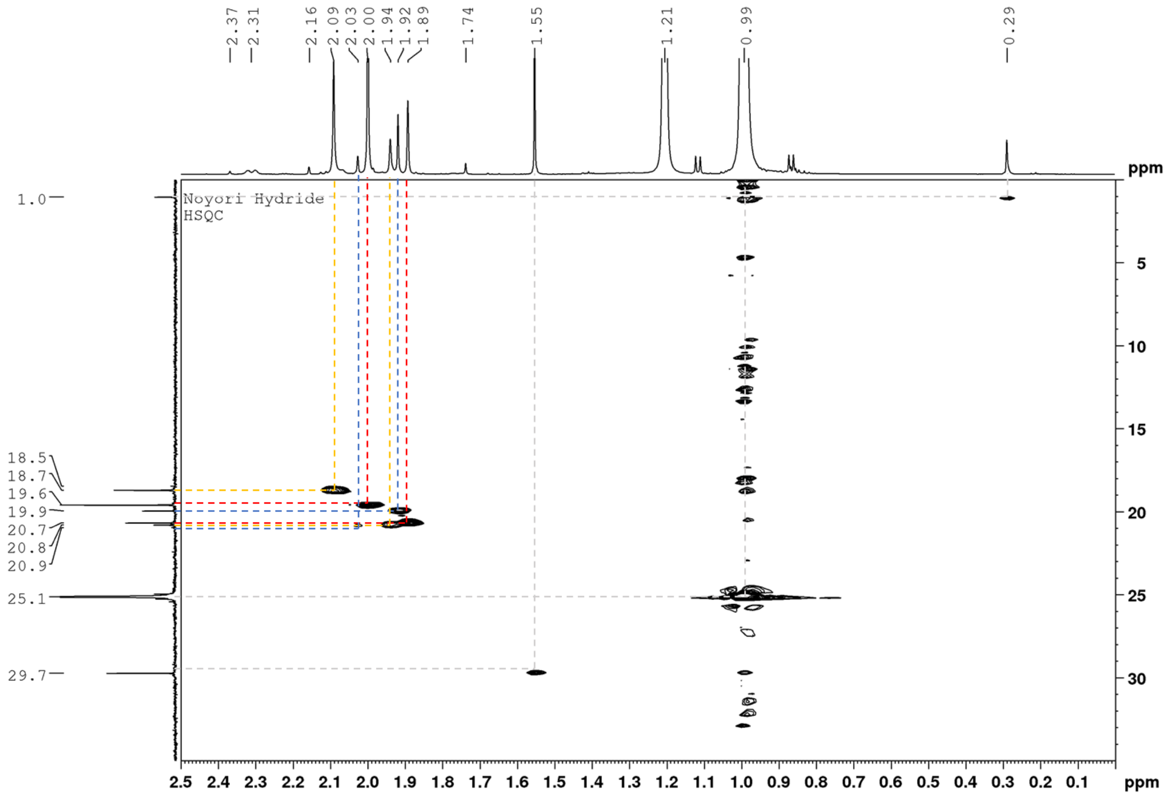

*Figure S18: Expansion of 0 – 2.5 ppm region of ^{1}H-^{13}C HSQC spectrum of a mixture of complexes **3a** and **3b**.*

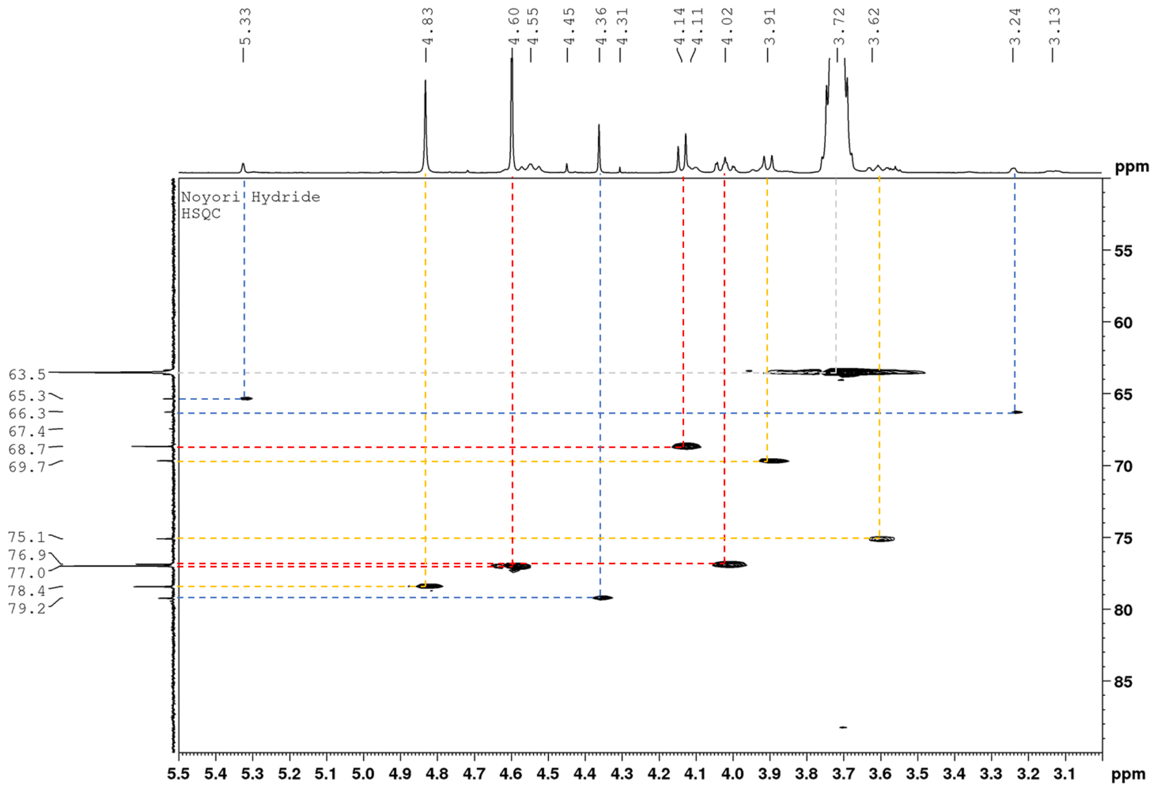

*Figure S19: Expansion of 3 – 5.5 ppm region of ^{1}H-^{13}C HSQC spectrum of a mixture of complexes **3a** and **3b**.*

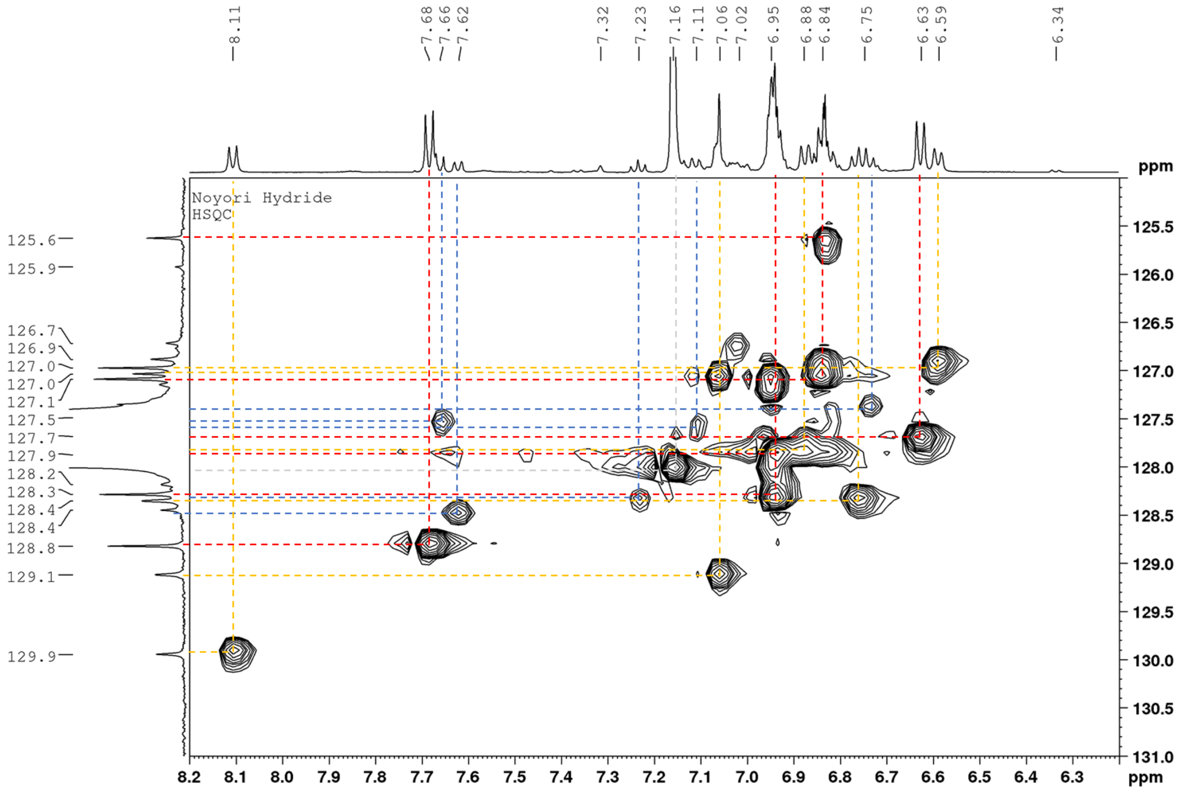

*Figure S20: Expansion of 6.2 – 8.2 ppm region of ^{1}H-^{13}C HSQC spectrum of a mixture of complexes **3a** and **3b**.*

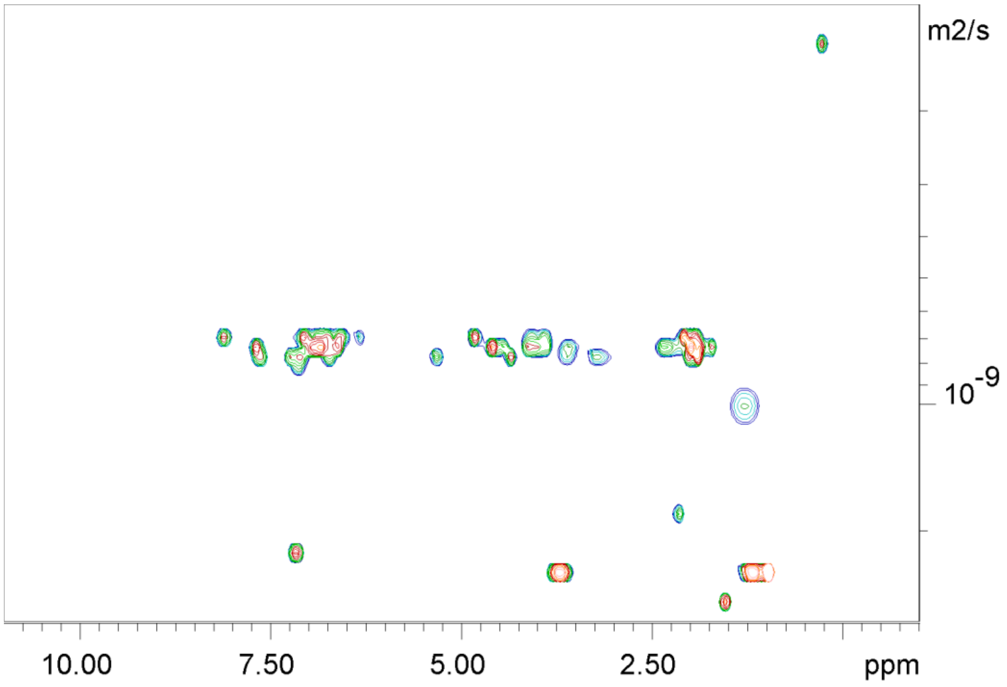

*Figure S21: DOSY spectrum of a mixture of complexes **3a** and **3b**.*

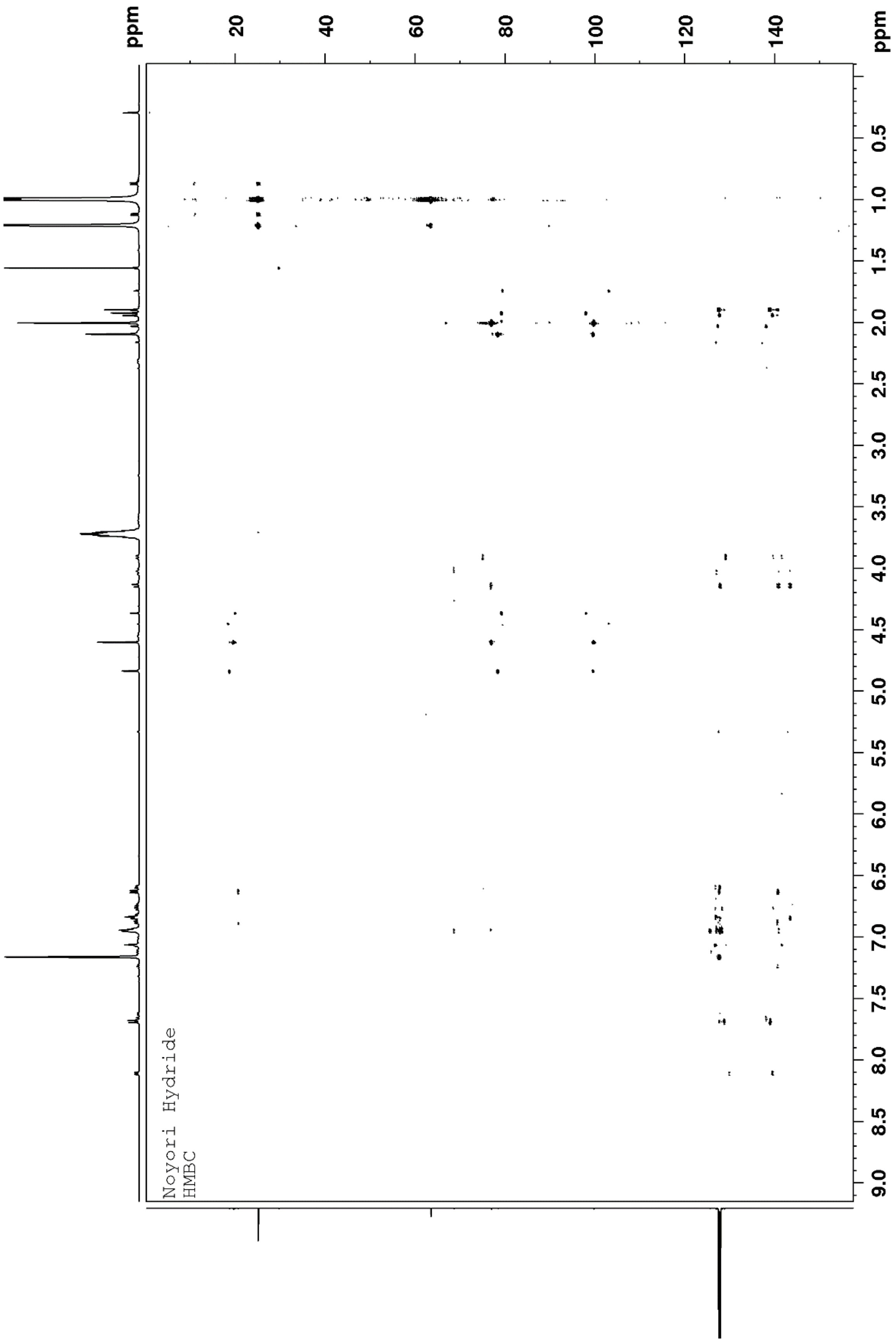

*Figure S22: ^{1}H-^{13}C HMBC spectrum of a mixture of complexes **3a** and **3b**.*

Hydrides **6a** and **6b**

The equilibrium position for the tethered catalyst resulted in a very low equilibrium concentration of hydrides **6a** and **6b**. As a result, the signal-to-noise ratio in the NMR spectra was much lower than for the non-tethered hydrides **3a** and **3b**. In C_6D_6 the equilibrium lay strongly towards hydride **6b**, with only small amounts of **6a** present in the sample. Adding an excess of propan-2-ol resulted in a shift in equilibrium position, closer to that observed in the kinetic reactions with propan-2-ol as reaction solvent.

No NOE peaks were observed for the hydride peak **6a** at -4.59 ppm (the major species during the initial stages of the reaction). This is attributed to the low concentration of this species in solution.

Hydride **6b** (-5.19 ppm) exhibited NOE interactions with the protons on the tosyl group at 7.34 ppm and to protons at 5.32 and 4.43 ppm on the Ru-arene ring. A NOE interaction was also observed between the hydride and a double-of-doublet peak at 3.88 ppm (J_{HH}= 11.4, 9.0 Hz). The COSY spectrum shows that the peak at 3.88 ppm is coupled to a doublet at 5.09 ppm (1H, J_{HH}= 9.0 Hz) and to the NH proton at 5.85 ppm (apparent triplet (broad), J_{HH}= 11.5 Hz). COSY shows that the NH peak is also coupling to a CH_2 group on the tether at 1.29 ppm (J_{HH}= 11.7 Hz). The peak at 3.88 ppm was therefore assigned to the C*H*NH proton and the peak at 5.09 ppm to the C*H*NTs proton.

The NOE interaction between hydride **6b** at -5.19 ppm and the C*H*NH proton indicates that hydride **6b** is the *(R,R)R* diastereomer. It may therefore be inferred that hydride **6a** is the *(R,R)S* diastereomer and that the order of the hydride diastereomer peaks is the same as that for the non-tethered catalyst hydrides **3a** and **3b**.

*Table S2: Summary of chemical shifts, assignments, multiplicity, J-coupling and 2D correlations for a mixture containing hydrides **6a** and **6b**. Due to the low signal-to-noise ratio and overlapping peaks, only selected key peaks which could be unambiguously assigned are included. [a] Estimated integral due to overlapping peaks, [b] Weak.*

#	Environment	Species	δ ^{1}H (ppm)	Multi-plicity	J_{HH} (Hz)	Integral	Number of ^{1}H	δ ^{13}C (ppm)	^{1}H-^{1}H COSY correlations
**1**	Ru-H	***(R,R)R*-6b**	-5.19	s	-	1.00	1	-	-
**2**	Ru-H	***(R,R)S*-6a**	-4.59	s	-	0.15	1	-	-
**3**	NH-C<u>H</u>$_2$-CH$_2$	***(R,R)R*-6b**	1.29	d	11.9	2.3[a]	2	50.2	#5, 11
**4**	Tosyl-CH$_3$	***(R,R)R*-6b**	1.91	s	-	3.30	3	20.9	-
**5**	NH-CH$_2$-C<u>H</u>$_2$	***(R,R)R*-6b**	2.59	dd	11.8, 4.5	1.33	2	50.2	#3, 11[b]
**6**	C<u>H</u>-NH	***(R,R)R*-6b**	3.88	dd	11.5, 9.0	1.08	1	82.7	#8, 11
**7**	Ru-Arene CH	***(R,R)R*-6b**	4.43	dd	4.5, 2.0	1.03	1	72.5	#9
**8**	C<u>H</u>-NTs	***(R,R)R*-6b**	5.09	d	9.0	0.97	1	70.5	#6
**9**	Ru-Arene CH	***(R,R)R*-6b**	5.32	ddd	5.7, 4.6, 1.7	1.06	1	77.1	#7
**10**	Ru-Arene CH	***(R,R)R*-6b**	5.39	t	5.4	1.11	1	76.8	[7.02 ppm]
**11**	N<u>H</u>	***(R,R)R*-6b**	5.85	t (br)	11.5	1.17	1	-	#3, 6
**12**	Tosyl-CH (ortho)	***(R,R)R*-6b**	7.33	dd	7.8, 2.6	2.16	2	128.4	[6.86 ppm]

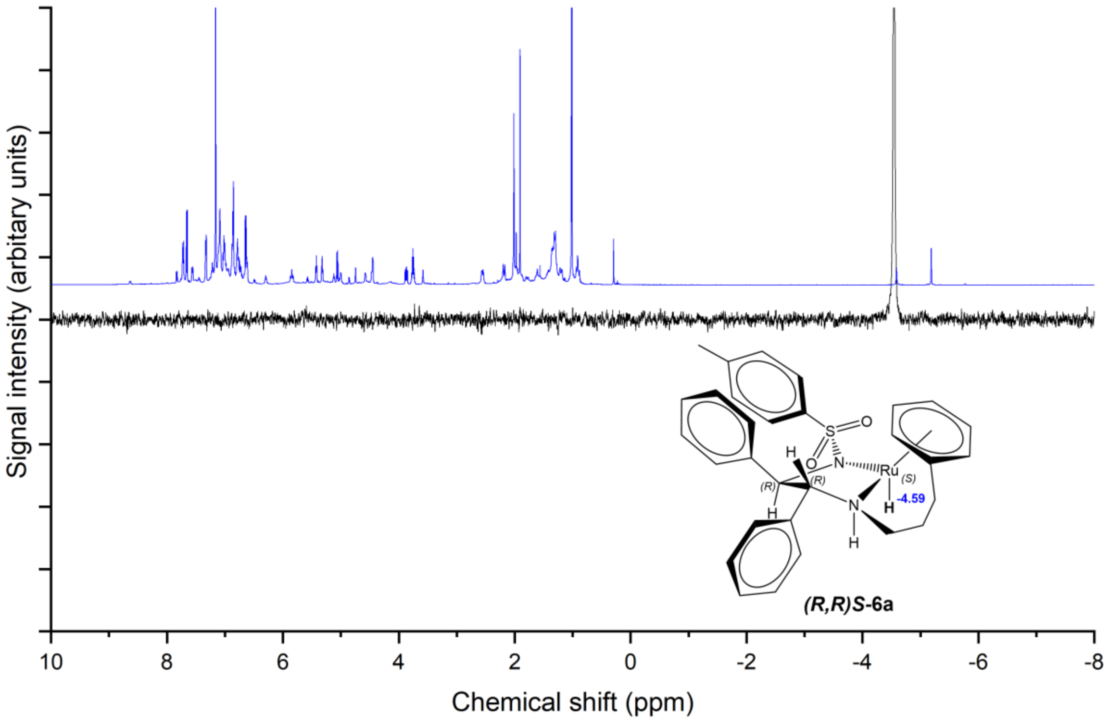

*Figure S23: ^{1}H and selective NOE NMR spectra of hydride **6a** (-4.59 ppm), showing the absence of NOE interactions of the hydride with other protons within the molecule, chemical shifts and assignment (66.7 mM (**4**), 97.5 mM NaO^{i}Pr, 0.6 mL C_6D_6, 25 °C, Ar atmosphere, conventional NMR tube). ^{1}H acquisition (64 scans, 1.64 s acquisition time, 1 s relaxation delay time, spectra processed with 0.3 Hz exponential line broadening). Selective NOE using a gradient spin echo pulse sequence with a shaped 180° pulse centred at -4.539 ppm (10240 scans, 2.18 s acquisition time, 2 s relaxation delay time, 80 ms Gaussian shaped pulse; spectra processed with 2.0 Hz exponential line broadening).*

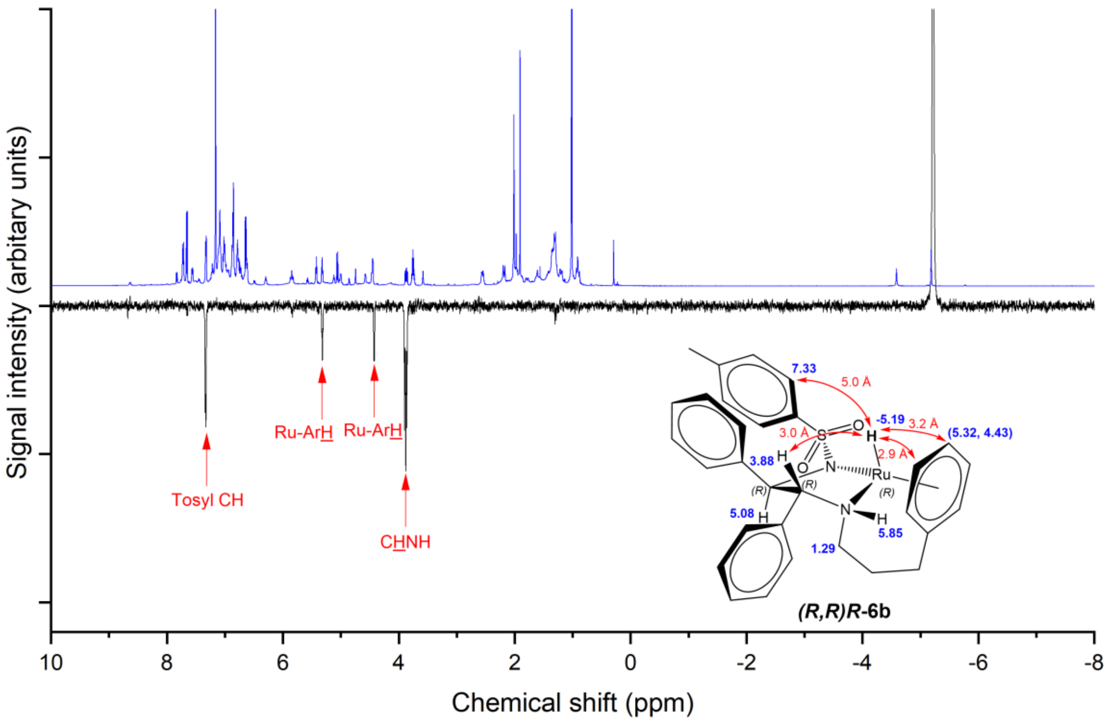

*Figure S24: ^{1}H and selective NOE NMR spectra of hydride **6b** (-5.19 ppm), showing NOE interactions of the hydride with other protons within the molecule, chemical shifts and assignment (66.7 mM (**4**), 97.5 mM NaO^{i}Pr, 0.6 mL C_6D_6, 25 °C, Ar atmosphere, conventional NMR tube). ^{1}H acquisition (64 scans, 1.64 s acquisition time, 1 s relaxation delay time, spectra processed with 0.3 Hz exponential line broadening). Selective NOE using a gradient spin echo pulse sequence with a shaped 180° pulse centred at -5.26 ppm (10240 scans, 2.18 s acquisition time, 2 s relaxation delay time, 80 ms Gaussian shaped pulse; spectra processed with 1.0 Hz exponential line broadening).*

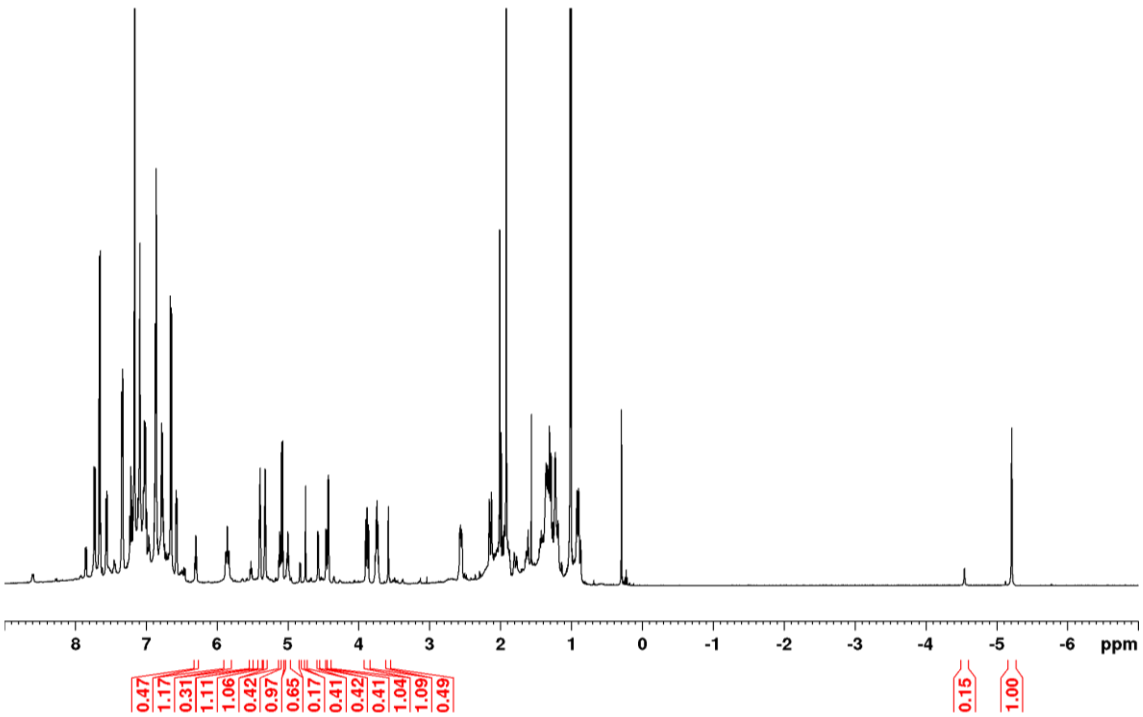

*Figure S25: ^{1}H NMR spectrum of a mixture containing complexes **6a** and **6b**.*

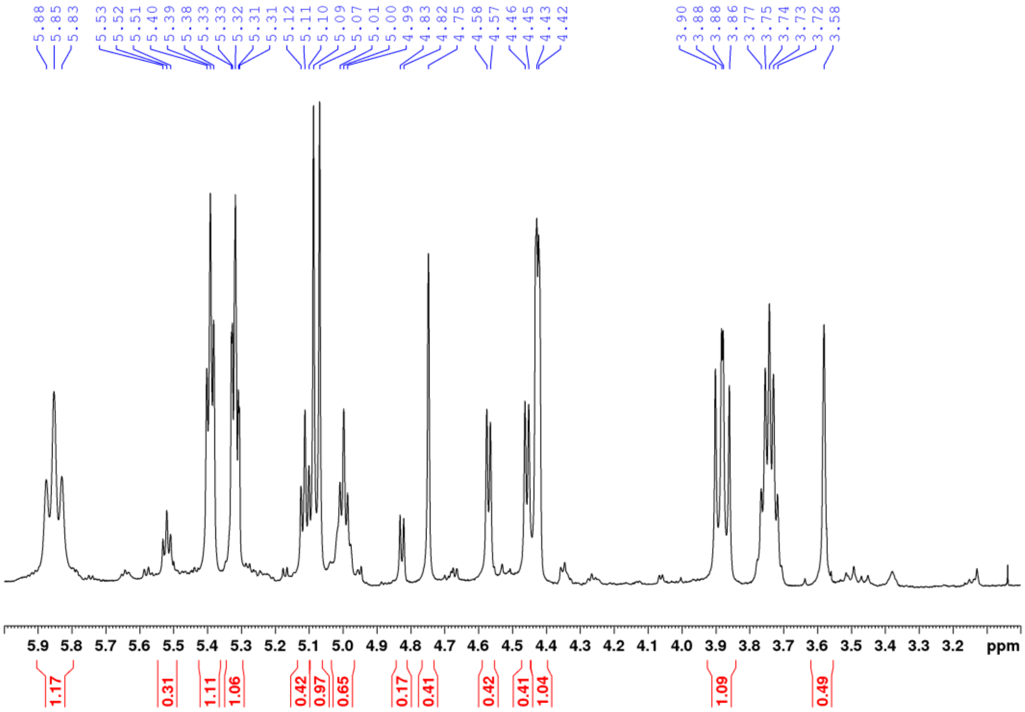

*Figure S26: Expansion of 3 – 6 ppm region of the ^{1}H NMR spectrum of a mixture containing complexes **6a** and **6b**.*

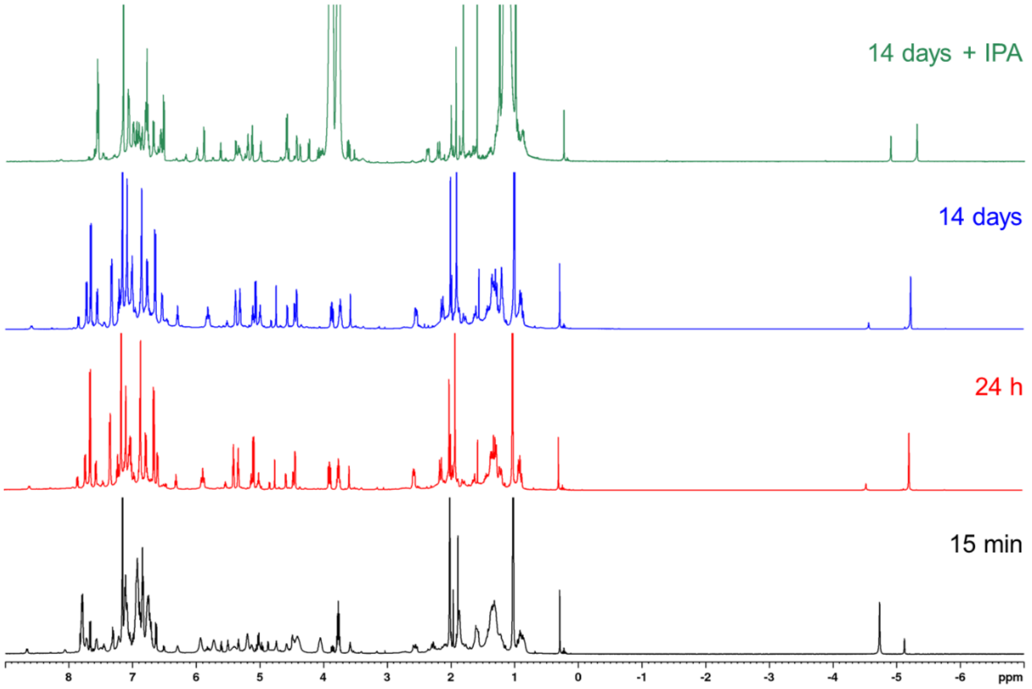

*Figure S27: ^{1}H NMR spectrum of a mixture containing complexes **6a** and **6b**: a) 15 min after preparation, b) 24 h after preparation, c) 14 days after preparation, d) after addition of 80 µL of propan-2-ol.*

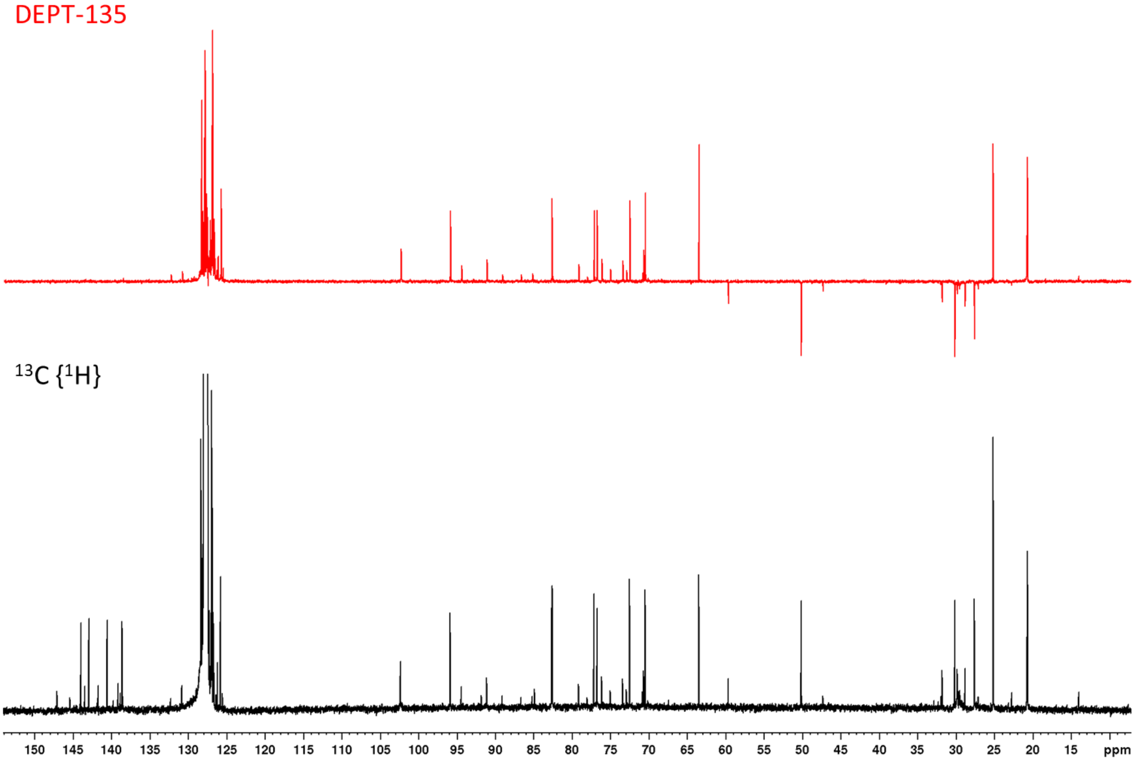

*Figure S28: a) ^{1}H decoupled ^{13}C and b) ^{13}C DEPT-135 NMR spectrum of a mixture containing complexes **6a** and **6b**.*

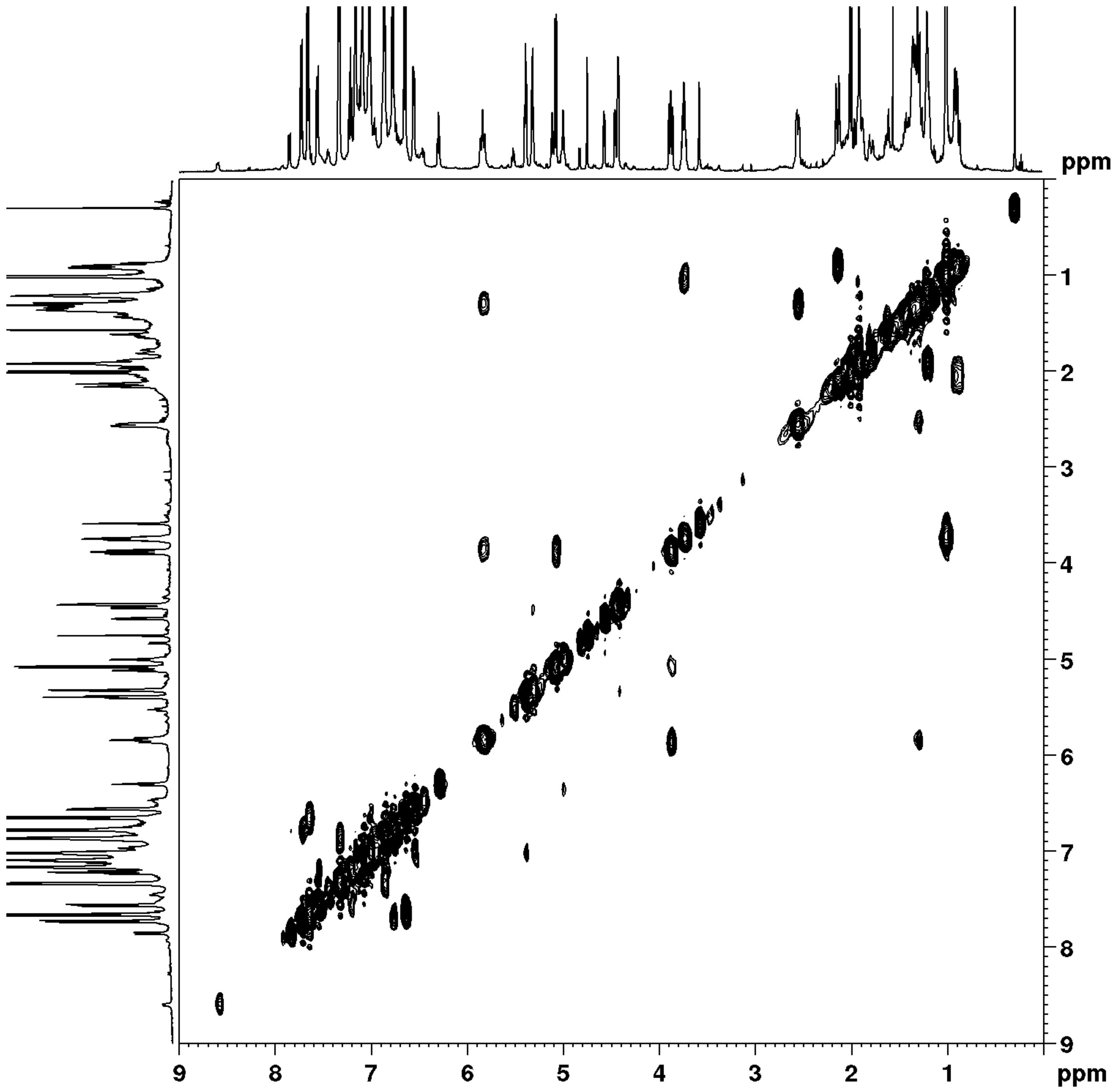

*Figure S29: ^{1}H-^{1}H COSY NMR spectrum of a mixture containing complexes **6a** and **6b**.*

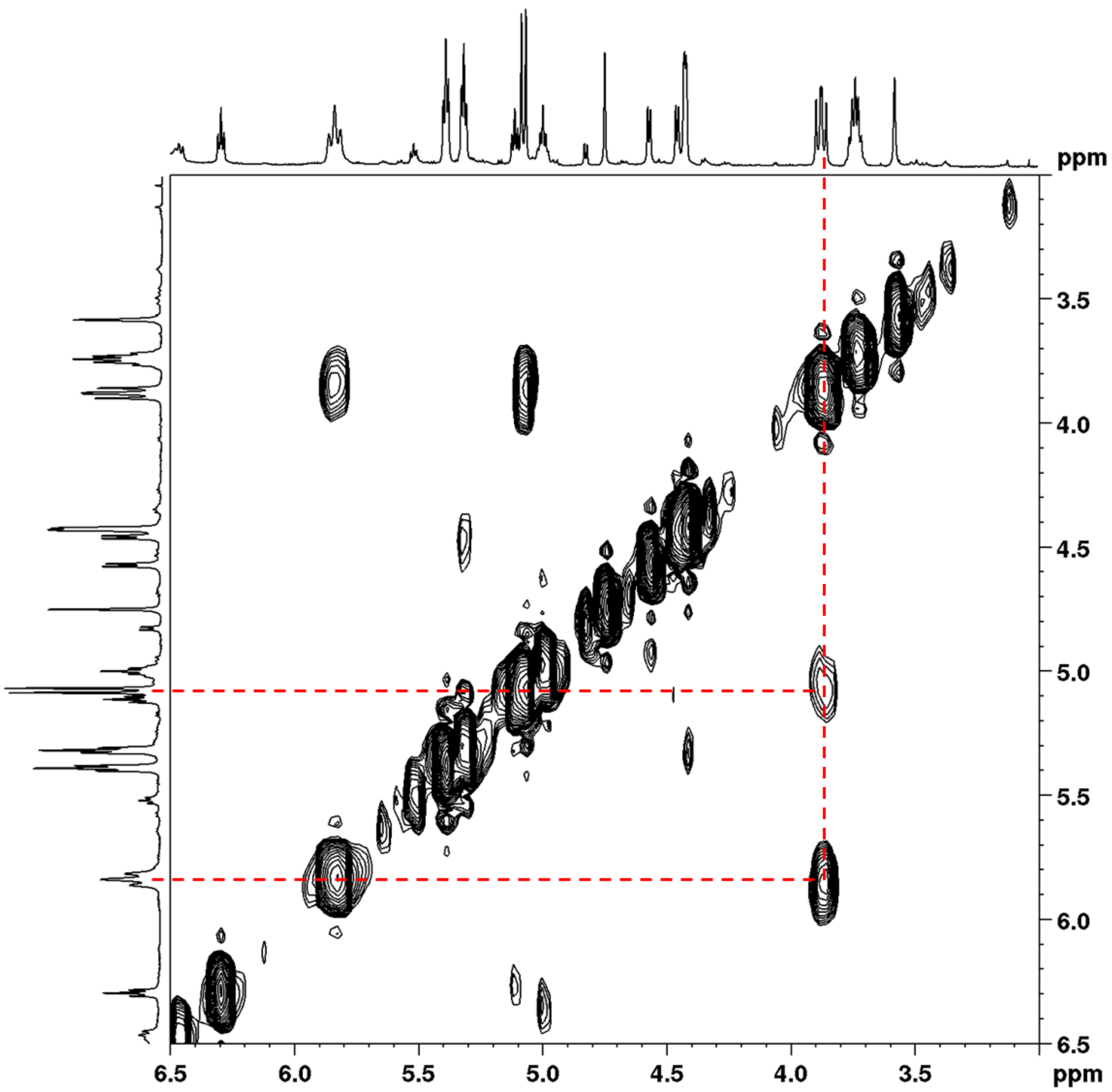

*Figure S30: Expansion of 3 – 6.5 ppm region of the ^{1}H-^{1}H COSY NMR spectrum of a mixture containing complexes **6a** and **6b**. $^{3}J_{HH}$ coupling cross peaks between the CH̲NH proton at 3.88 ppm and the NH̲ and CH̲NTs protons at 5.85 and 5.09 ppm are indicated.*

.

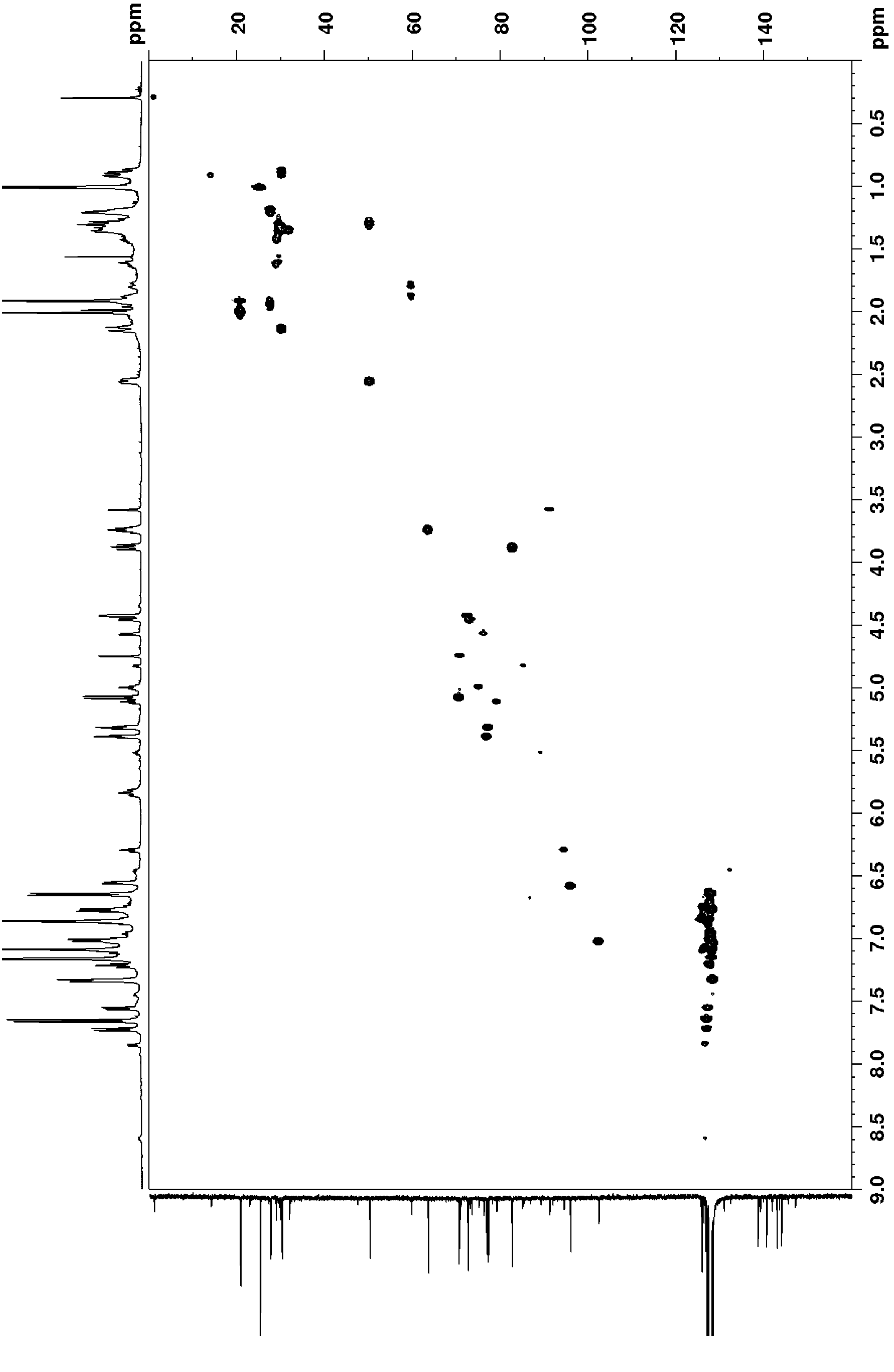

*Figure S31: ^{1}H-^{13}C HSQC NMR spectrum of a mixture containing complexes **6a** and **6b**.*

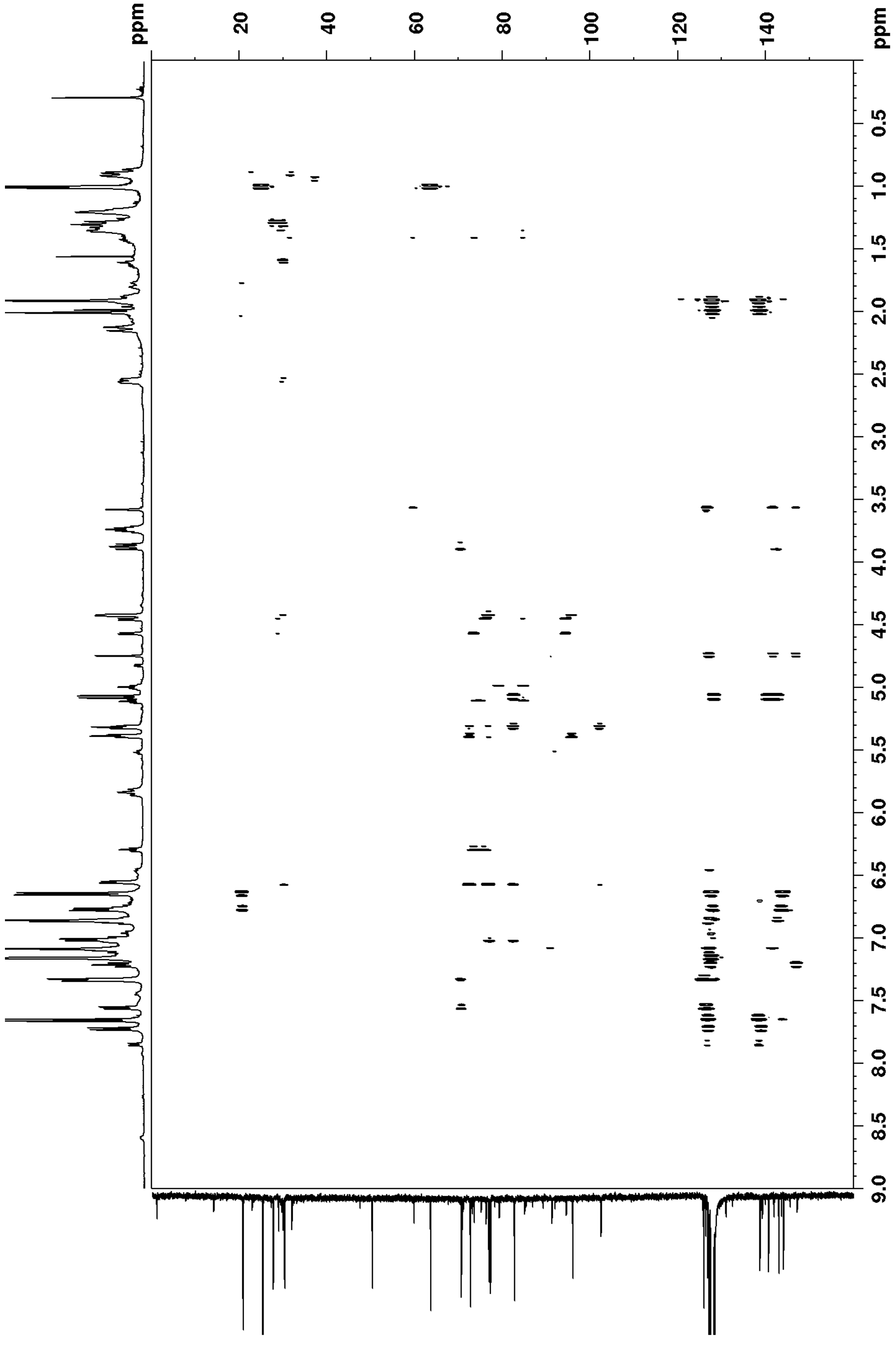

*Figure S32: ^{1}H-^{13}C HMBC NMR spectrum of a mixture containing complexes **6a** and **6b**.*

Density functional theory calculations

Density Functional Theory calculations were carried out using the Gaussian 09 (rev. D.01-v3) [8] software package. Geometry optimisation, frequency and NMR shift calculations were performed in a SMD polarizable continuum solvent model[9] of isopropanol. Restricted rωB97X-D, rB3PW91, rPBE1PBE and rM06L functionals, along with SDD or LANL2DZ (Ru) and 6-311++G(d,p) or 6-311+G(d) basis sets were used as indicated. Starting geometries were taken from published crystal structures,[10] or calculated geometries,[11-13] where available. DFT integration grids with 99 radial and 590 angular points (Ultrafine) were used for all transition state calculations. All ground state geometries were free of imaginary frequencies. Calculation output files are available to download from DOI: 10.6084/m9.figshare.14473035.

Prediction of ^{1}H NMR shifts

The calculation of isotropic NMR shifts requires fully energy minimised structures calculated at a high level of theory to gain accurate results. For this study, an SDD effective core potential basis set was used for ruthenium, with a 6-311++G(d,p) basis set on the arene, ligand backbone and heteroatoms and a 6-31+G(d) basis set for the phenyl rings. This basis set combination has previously been reported to provide good energy and frequency prediction results with ruthenium hydrides.[11, 14-15]

To convert the calculated isotropic shifts into predicted NMR chemical shifts, a calibration model is needed. A linear correction curve, constructed from a library of ruthenium hydride complexes with known NMR shifts is reported to give accurate results for a range of ruthenium hydrides.[16] For each of the four functionals used to calculate the NMR shifts, a range of ruthenium hydride complexes with known NMR shifts were also optimised and used to produce a calibration curve (Figure S32). This calibration curve was then used to adjust the calculated isotropic NMR shifts predicted by Gaussian.

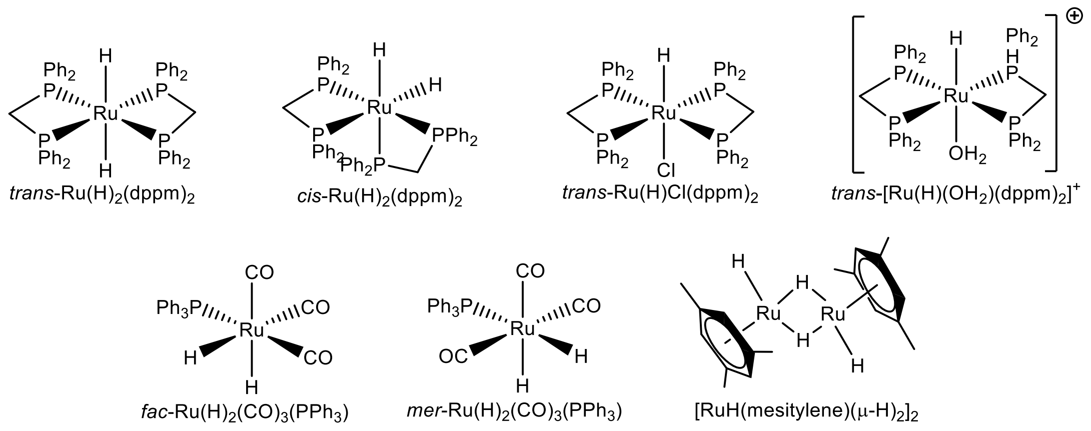

Scheme S3: Ruthenium hydride complexes used to build calibration curves for NMR shift prediction

Table S3: Literature[16-17] and calculated [SDD/6-311++G(d,p)/6-31+G(d)] chemical shift values for ruthenium hydride calibration complexes shown in Scheme S1. Values in brackets are uncorrected isotropic shifts.

Compound	Chemical shift (ppm)				
	Literature	rωB97X-D	rB3PW91	rPBE1PBE	rM06L
***trans*-Ru(H)$_2$(dppm)$_2$**	**-4.7**	-5.23 (38.83)	-2.98 (33.73)	-5.33 (35.35)	7.53 (36.53)
***cis*-Ru(H)$_2$(dppm)$_2$**	**-7.4**	-7.40 (36.75)	-	-	-
***trans*-Ru(H)Cl(dppm)$_2$**	**-14.0**	-13.88 (45.03)	-14.00 (42.12)	-14.00 (42.48)	-14.53 (43.04)
***trans*-[Ru(H)(OH$_2$)(dppm)$_2$]$^+$**	**-18.8**	-15.43 (46.52)	-	-17.17 (45.09)	-16.63 (44.99)
***fac*-Ru(H)$_2$(CO)$_3$(PPh$_3$)**	**-6.7**	-5.23 (36.75)	-6.69 (36.10)	-6.09 (36.23)	-6.20 (35.30)
	**-6.7**	-6.58 (37.09)	-6.84 (36.57)	-6.70 (36.63)	-6.70 (35.76)
***mer*-Ru(H)$_2$(CO)$_3$(PPh$_3$)**	**-6.7**	-5.51 (38.09)	-6.79 (37.65)	-6.74 (37.51)	-6.95 (35.99)
	**-7.3**	-6.65 (36.90)	-7.15 (36.60)	-8.13 (36.48)	-6.95 (35.99)
**[RuH(mesitylene)(μ-H)$_2$]$_2$**	**-1.52**	-1.03 (32.73),	-	-	-
		-1.88 (33.54)			
	**-17.18**	-18.14 (49.12),	-	-	-
		-18.24 (49.21)			

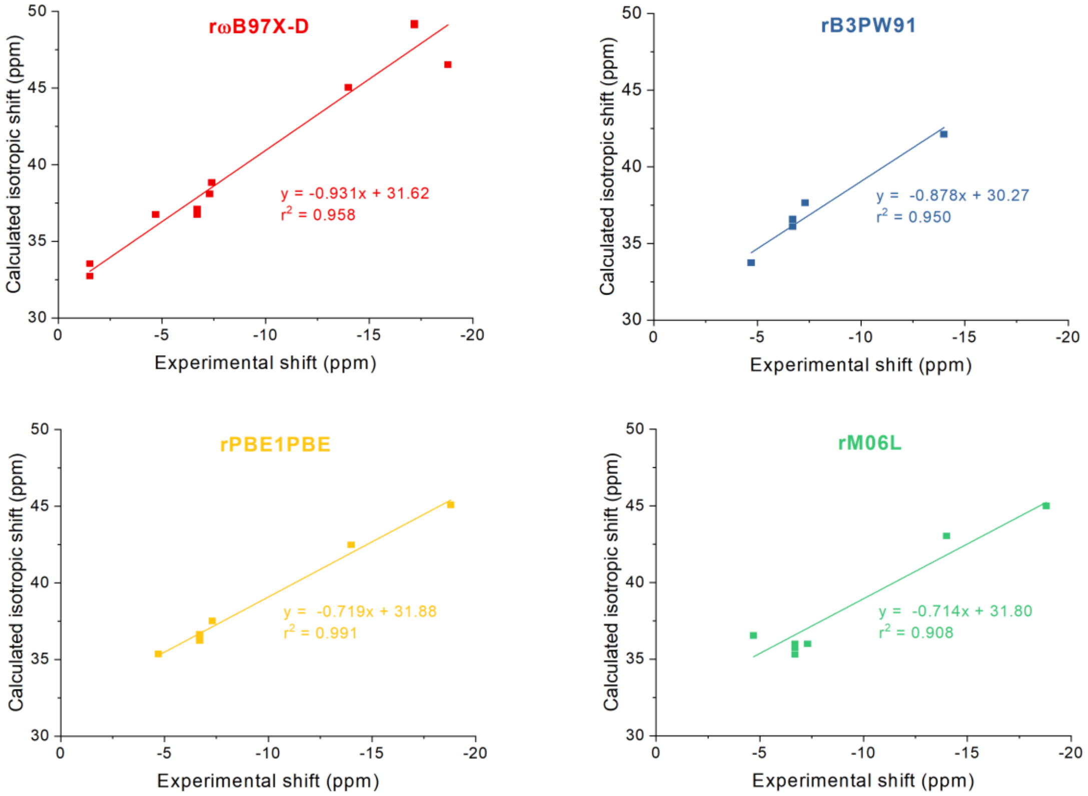

Figure S33: Calibration curves for the rωB97X-D, rB3PW91, rPBE1PBE and rM06L functionals.

*Table S4: Experimental (C_6D_6) NMR shifts, calculated isotropic shifts and difference in ground state free energies [SDD/6-311++G(d,p)/6-31+G(d)] for complexes **(R,R)S-3a** and **(R,R)R-3b**. Values in brackets are uncorrected isotropic shifts.*

Functional	Solvent	Predicted Chemical Shift (ppm)		$\Delta G_{(R,R)S-(R,R)R}$ (kcal/mol)
		(R,R)S-3a	*(R,R)R*-3b	
**Experimental**	**Isopropanol-h$_8$**	**-5.26**	**-6.54**	**-**
**rωB97X-D**	Isopropanol-h$_8$	-2.87 (34.29)	-2.92 (34.34)	-4.71
**rPBE1PBE**	Isopropanol-h$_8$	-2.32 (33.55)	-3.36 (34.29)	-4.06
**rB3PW91**	Isopropanol-h$_8$	-3.78 (33.59)	-4.73 (34.43)	-5.93
**rM06L**	Isopropanol-h$_8$	-1.43 (32.82)	-1.58 (32.93)	-4.21
**rωB97X-D**	None	(32.30)	(33.86)	-
**rωB97X-D**	C_6H_6	(32.97)	(34.57)	-

*Table S5: Experimental (C_6D_6) NMR shifts, calculated isotropic shifts and difference in ground state free energies [SDD/6-311++G(d,p)/6-31+G(d)] for complexes **(R,R)S-6a** and **(R,R)R-6b**. Values in brackets are uncorrected isotropic shifts.*

Functional	Solvent	Predicted Chemical Shift (ppm)		$\Delta G_{(R,R)S-(R,R)R}$ (kcal/mol)
		(R,R)S-6a	*(R,R)R*-6b	
**Experimental**	**Isopropanol-h$_8$**	**-5.26**	**-5.51**	**-**
**rωB97X-D**	Isopropanol-h$_8$	-2.02 (33.50)	-1.39 (32.91)	-10.35
**rPBE1PBE**	Isopropanol-h$_8$	-1.56 (33.00)	-1.21 (32.75)	-11.14
**rB3PW91**	Isopropanol-h$_8$	-3.63 (33.45)	-2.83 (32.75)	-11.48
**rM06L**	Isopropanol-h$_8$	-0.14 (31.90)	-0.57 (32.21)	-9.27
**rB3LYP**	Isopropanol-h$_8$	(33.98)	(33.05)	-12.51
**rM06**	Isopropanol-h$_8$	(32.41)	(32.15)	-8.74
**rMPW1PW91**	Isopropanol-h$_8$	(33.58)	(32.87)	-11.62

Reaction pathway

Geometry optimisation, energy and frequency calculations were performed using the restricted rM06L functional the LANL2DZ basis set used for ruthenium and 6-31+G(d) basis set for all other atoms. The tosyl CH_3 group was excluded from all calculations.

*Table S6: Calculated free energies for ground, ion pair and transition state geometries in the (R,R)S and (R,R)R reaction pathways for catalyst **1** [rM06L, LANL2DZ/6-31+G(d)].*

Shared		G (Har)
Acetophenone	Acp	-384.759171
S-1-phenylethanol	S-PE	-385.925696
R-1-phenylethanol	R-PE	-385.926055
Acetone	Ac	-193.084831
Isopropanol	IPA	-194.253292
(R,R)-Noyori-Unsat (no tosyl methyl)	(RR)-2	-1874.60779

(R,R)S **pathway**		G (Har)
(R,R)-Noyori-(S)-Hydride	(RRS)-3	-1875.78758
(R,R)-Noyori-(pro-S)-Unsat+ IPA Ion pair	(RRS)-2'	-2068.85077
(R,R)-Noyori-(pro-S)-Unsat+ IPA TS	(RRS)-TS(2,3)	-2068.84566
(R,R)-Noyori-(S)-Hydride + Acetone Ion pair	(RRS)-3'	-2068.8602
(R,R)-Noyori-(S)-Hydride + Acetophenone-(pro-S) Ion pair	(RRS)(S)-3'	-2260.5326
(R,R)-Noyori-(S)-Hydride + Acetophenone-(pro-S) TS	(RRS)(S)-TS(3,2)	-2260.51839
(R,R)-Noyori-(pro-S)-Unsat + (S)-1-PE Ion pair	(RRS)(S)-2'	-2260.51851
(R,R)-Noyori-(S)-Hydride + Acetophenone-(pro-R) Ion pair	(RRS)(R)-3'	-2260.53622
(R,R)-Noyori-(S)-Hydride + Acetophenone-(pro-R) TS	(RRS)(R)-TS(3,2)	-2260.52155
(R,R)-Noyori-(pro-S)-Unsat + (R)-1-PE Ion pair	(RRS)(R)-2'	-2260.52479

(R,R)R **pathway**		G (Har)
(R,R)-Noyori-(R)-Hydride	(RRR)-3	-1875.78158
(R,R)-Noyori-(pro-R)-Unsat+ IPA Ion pair	(RRR)-2'	-2068.84692
(R,R)-Noyori-(pro-R)-Unsat+ IPA TS	(RRR)-TS(2,3)	-2068.83937
(R,R)-Noyori-(R)-Hydride + Acetone Ion pair	(RRR)-3'	-2068.85867
(R,R)-Noyori-(R)-Hydride + Acetophenone-(pro-S) Ion pair	(RRR)(S)-3'	-2260.53445
(R,R)-Noyori-(R)-Hydride + Acetophenone-(pro-S) TS	(RRR)(S)-TS(3,2)	-2260.51523
(R,R)-Noyori-(pro-R)-Unsat + (S)-1-PE Ion pair	(RRR)(S)-2'	-2260.51707
(R,R)-Noyori-(R)-Hydride + Acetophenone-(pro-R) Ion pair	(RRR)(R)-3'	-2260.53143
(R,R)-Noyori-(R)-Hydride + Acetophenone-(pro-R) TS	(RRR)(R)-TS(3,2)	-2260.51451
(R,R)-Noyori-(pro-R)-Unsat + (R)-1-PE Ion pair	(RRR)(R)-2'	-2260.51858

*Table S7: Calculated relative free energy differences for ground, ion pair and transition state geometries in the (R,R)S and (R,R)R reaction pathways for catalyst **1** [rM06L, LANL2DZ/6-31+G(d)].*

	ΔG (kJ/mol)	ΔG (kcal/mol)	Species	ΔG (kcal/mol)	ΔG (kJ/mol)
	S-1-phenylethanol			R-1-phenylethanol	
(R,R)S catalyst	5.08	1.21	(RR)-2 + Ac + PE	0.99	4.14
	44.38	10.61	(RRS)-2' + Ac	6.67	27.90
	44.72	10.69	(RRS)-TS(3,2) + Ac	8.71	36.42
	7.41	1.77	(RRS)-3' + Ac	-0.50	-2.10
	-29.75	-7.11	(RRS)-3 + Ac + Acp	-7.11	-29.75
	2.31	0.55	(RRS)-3' + Acp	0.55	2.31
	40.48	9.67	(RRS)-TS(2,3) + Acp	9.67	40.48
	27.07	6.47	(RRS)-2' + Acp	6.47	27.07
	0.00	0.00	**(RR)-2 + IPA + Acp**	0.00	0.00
(R,R)R catalyst	37.16	8.88	(RRR)-2' + Acp	8.88	37.16
	56.99	13.62	(RRR)-TS(2,3) + Acp	13.62	56.99
	6.33	1.51	(RRR)-3' + Acp	1.51	6.33
	-13.99	-3.34	(RRR)-3 + Ac + Acp	-3.34	-13.99
	2.53	0.61	(RRR)-3' + Ac	2.50	10.47
	53.02	12.67	(RRR)-TS(3,2) + Ac	13.12	54.88
	48.17	11.51	(RRR)-2' + Ac	10.56	44.20
	5.08	1.21	(RR)-2 + Ac + PE	0.99	4.14

*Table S8: Comparison between free energy barriers for (RRR)(S)-TS(3,2) and (RRR)(R)-TS(3,2) transition states for catalyst **1** calculated using different functionals [Basis set: LANL2DZ/6-31+G(d)].*

Functional	Predicted Transition state energy (Har)		$\Delta G_{(RRR)(S)} - \Delta G_{(RRR)(R)}$ (kcal/mol)
	(RRR)(S)-TS(3,2)	(RRR)(R)-TS(3,2)	
**rM06L**	-2260.52	-2260.51	-0.44616
**rB3PW91**	-2260.03	-2260.03	-0.58107
**PBE0**	-2258.48	-2258.48	-1.05359
**rωB97X-D**	-2260.14	-2260.14	-1.1201

Energy span calculations

Catalyst stereochemistry

$$TOF = \frac{k_B T}{h} e^{\Delta G/RT}$$

(R,R)S **pathway:**

ΔG = 9.67 kcal/mol = 40459 J/mol

$$TOF = \frac{1.381 \times 10^{-23} \ \times 298}{6.626 \times 10^{-34}} e^{\frac{-40459}{8.314 \times 298}} = 5.02 \times 10^{5}\ s^{-1}$$

(R,R)R **pathway:**

ΔG = 13.62 kcal/mol = 56986 J/mol

$$TOF = \frac{1.381 \times 10^{-23} \ \times 298}{6.626 \times 10^{-34}} e^{\frac{-56986}{8.314 \times 298}} = 6.37 \times 10^{2}\ s^{-1}$$

Ratio between two different pathways:

$$\frac{5.02 \times 10^{5}\ s^{-1}}{6.37 \times 10^{2}\ s^{-1}} = 789.3$$

Product enantioselectivity

(R,R)S pathway

***S*-1-phenylethanol:**

ΔG = 10.69 kcal/mol = 44727 J/mol

$$TOF = \frac{1.381 \times 10^{-23} \times 298}{6.626 \times 10^{-34}} e^{\frac{-44727}{8.314 \times 298}} = 8.97 \times 10^{4}\ s^{-1}$$

***R*-1-phenylethanol:**

ΔG = 8.71 kcal/mol = 36442 J/mol

$$TOF = \frac{1.381 \times 10^{-23} \times 298}{6.626 \times 10^{-34}} e^{\frac{-36442}{8.314 \times 298}} = 2.54 \times 10^{6}\ s^{-1}$$

Enantioselectivity:

$$ee = \frac{2.54 \times 10^{6}\ s^{-1} - 8.97 \times 10^{4}\ s^{-1}}{8.97 \times 10^{4}\ s^{-1} + 2.54 \times 10^{6}\ s^{-1}} \times 100 = 93.2\%$$

(R,R)R pathway

***S*-1-phenylethanol:**

ΔG = 12.67 kcal/mol = 53011 J/mol

$$TOF = \frac{1.381 \times 10^{-23} \times 298}{6.626 \times 10^{-34}} e^{\frac{-53011}{8.314 \times 298}} = 3.17 \times 10^{3}\ s^{-1}$$

***R*-1-phenylethanol:**

ΔG = 13.12 kcal/mol = 54894 J/mol

$$TOF = \frac{1.381 \times 10^{-23} \times 298}{6.626 \times 10^{-34}} e^{\frac{-54894}{8.314 \times 298}} = 1.48 \times 10^{3}\ s^{-1}$$

Enantioselectivity:

$$ee = \frac{1.48 \times 10^{3}\ s^{-1} - 3.17 \times 10^{3}\ s^{-1}}{3.17 \times 10^{3}\ s^{-1} + 1.48 \times 10^{3}\ s^{-1}} \times 100 = -36.3\%$$

References

1. Bennett, M. A.; Smith, A. K., Arene ruthenium(II) complexes formed by dehydrogenation of cyclohexadienes with ruthenium(III) trichloride. *J. Chem. Soc., Dalton Trans.* **1974**, 233.

2. Tietze, Lutz F.; Zhou, Y.; Töpken, E., Synthesis of Simple Enantiopure Tetrahydro-β-carbolines and Tetrahydroisoquinolines. *Eur. J. Org. Chem.* **2000,** *2000*, 2247-2252.

3. Andna, L.; Miesch, L., Trapping of N-Acyliminium Ions with Enamides: An Approach to Medium-Sized Diaza-Heterocycles. *Org. Lett.* **2018,** *20*, 3430-3433.

4. Haack, K.-J.; Hashiguchi, S.; Fujii, A.; Ikariya, T.; Noyori, R., The Catalyst Precursor, Catalyst and Intermediate in the Ru(II)-Promoted Asymmetric Hydrogen Transfer between Alcohols and Ketones. *Angew. Chem. Int. Ed.* **1997,** *36*, 285-288.

5. Hall, A. M. R.; Chouler, J. C.; Codina, A.; Gierth, P. T.; Lowe, J. P.; Hintermair, U., Practical Aspects of Real-time Reaction Monitoring using Multi-nuclear High Resolution FlowNMR Spectroscopy. *Cat. Sci. Tech.* **2016,** *6*, 8406-8417.

6. Mo, H.; Harwood, J. S.; Raftery, D., Receiver gain function: the actual NMR receiver gain. *Magn. Reson. Chem.* **2010,** *48*, 235-8.

7. Berry, D. B. G.; Codina, A.; Clegg, I.; Lyall, C. L.; Lowe, J. P.; Hintermair, U., Insight into catalyst speciation and hydrogen co-evolution during enantioselective formic acid-driven transfer hydrogenation with bifunctional ruthenium complexes from multi-technique operando reaction monitoring. *Faraday Discuss.* **2019,** *220*, 45-57.

8. Frisch, M. J.; Trucks, G. W.; Schlegel, H. B.; Scuseria, G. E.; Robb, M. A.; Cheeseman, J. R.; Scalmani, G.; Barone, V.; Mennucci, B.; Petersson, G. A. H. N., M. Caricato, X. Li, H. P. Hratchian, A. F. Izmaylov, J. Bloino, G. Zheng, J. L. Sonnenberg, M. Hada, M. Ehara, K. Toyota, R. Fukuda, J. Hasegawa, M. Ishida, T. Nakajima, Y. Honda, O. Kitao, H. Nakai, T. Vreven, J. A. Montgomery, Jr., J. E. Peralta, F. Ogliaro, M. Bearpark, J. J. Heyd, E. Brothers, K. N. Kudin, V. N. Staroverov, T. Keith, R. Kobayashi, J. Normand, K. Raghavachari, A. Rendell, J. C. Burant, S. S. Iyengar, J. Tomasi, M. Cossi, N. Rega, J. M. Millam, M. Klene, J. E. Knox, J. B. Cross, V. Bakken, C. Adamo, J. Jaramillo, R. Gomperts, R. E. Stratmann, O. Yazyev, A. J. Austin, R. Cammi, C. Pomelli, J. W. Ochterski, R. L. Martin, K. Morokuma, V. G. Zakrzewski, G. A. Voth, P. Salvador, J. J. Dannenberg, S. Dapprich, A. D. Daniels, O. Farkas, J. B. Foresman, J. V. Ortiz, J. Cioslowski, and D. J. Fox *Gaussian 09, Revision D.01*, Gaussian Inc: Wallingford CT, 2009.

9. Marenich, A. V.; Cramer, C. J.; Truhlar, D. G., Universal solvation model based on solute electron density and on a continuum model of the solvent defined by the bulk dielectric constant and atomic surface tensions. *J. Phys. Chem. B* **2009,** *113*, 6378-6396.

10. Noyori, R.; Hashiguchi, S., Asymmetric Transfer Hydrogenation Catalyzed by Chiral Ruthenium Complexes. *Acc. Chem. Res.* **1997,** *30*, 97-102.

11. Dub, P. A.; Gordon, J. C., The mechanism of enantioselective ketone reduction with Noyori and Noyori-Ikariya bifunctional catalysts. *Dalton Transactions* **2016,** *45*, 6756-6781.

12. Dub, P. A.; Ikariya, T., Quantum chemical calculations with the inclusion of nonspecific and specific solvation: asymmetric transfer hydrogenation with bifunctional ruthenium catalysts. *J. Am. Chem. Soc.* **2013,** *135*, 2604-2619.

13. Yamakawa, M.; Ito, H.; Noyori, R., The Metal−Ligand Bifunctional Catalysis: A Theoretical Study on the Ruthenium(II)-Catalyzed Hydrogen Transfer between Alcohols and Carbonyl Compounds. *J. Am. Chem. Soc.* **2000,** *122*, 1466-1478.

14. Dub, P. A.; Henson, N. J.; Martin, R. L.; Gordon, J. C., Unravelling the mechanism of the asymmetric hydrogenation of acetophenone by [RuX_2(diphosphine)(1,2-diamine)] catalysts. *J. Am. Chem. Soc.* **2014,** *136*, 3505-3521.

15. Dub, P. A.; Scott, B. L.; Gordon, J. C., Why Does Alkylation of the N-H Functionality within M/NH Bifunctional Noyori-Type Catalysts Lead to Turnover? *J. Am. Chem. Soc.* **2017,** *139*, 1245-1260.

16. del Rosal, I.; Maron, L.; Poteau, R.; Jolibois, F., DFT calculations of ^{1}H and ^{13}C NMR chemical shifts in transition metal hydrides. *Dalton Trans* **2008**, 3959-3970.

17. Bennett, M. A.; Ennett, J. P.; Gell, K. I., Dinuclear μ-hydrido arene complexes of ruthenium(II). *J. Organomet. Chem.* **1982,** *233*, C17-C20.
